# Developing Researchers’ Competencies through CARE–KNOW–DO and upSkill.Map, aligned with EU and UNESCO Priorities

**DOI:** 10.12688/openreseurope.21408.1

**Published:** 2025-10-31

**Authors:** Alexandra Okada, Kieron Sheehy, Klaus - Rossade, Arosha K. Bandara

**Affiliations:** 1 Faculty of Wellbeing, Education and Language Studies (WELS), The Open University, Milton Keynes, MK 76AA, UK; 2 Faculty of Science, Technology, Engineering and Mathematics (STEM), The Open University, Milton Keynes, England, UK

**Keywords:** Researchers’ Competencies, Doctoral Education, CARE-KNOW-DO framework, EU Agenda, UNESCO SDGs

## Abstract

**Background:**

In an era of unprecedented global challenges—climate breakdown, widening inequalities, geopolitical instability, and technological disruption—the research community faces mounting pressure to deliver not only disciplinary excellence but also sustainable and socially relevant solutions. Traditional competency frameworks, including the European Commission's ResearchComp, Vitae's Researcher Development Framework, and the OECD's Core Competency Framework, have made significant strides in recognizing transversal skills. However, inadequate training for real world problems and limited academic opportunities are deepening global skills mismatches, while these frameworks rarely position sustainability, equity, and justice as central pillars, creating a critical gap in preparing researchers for contemporary challenges.

**Methods:**

This mixed-method study addresses this limitation by introducing upSkill.Map, a validated, interactive self-assessment instrument designed to foster responsible, innovative, and "eco-outwards" research careers. The novel tool uniquely integrates personal, professional, and planetary dimensions, operationalizing the CARE–KNOW–DO model through eight competency clusters encompassing 28 transversal skills. This framework guides researchers to reflect on values and purpose (CARE), deepen knowledge and understanding (KNOW), and apply skills through practice and societal engagement (DO). Rigorous validation combined psychometric analysis (exploratory factor analysis and reliability testing, Cronbach’s α = 0.963), expert review, and comprehensive user feedback, confirming a stable structure, strong validity, and conceptual coherence.

**Results:**

Five key competency factors emerged: 1. Responsible and Policy-Engaged Research; 2. Collaborative and Inclusive Research Leadership; 3. Interdisciplinary Networking for Research Innovation; 4. Methodological Breadth with Societal Impact; and 5. Resilient Capacity Development. Findings from 40 researchers revealed consistent prioritisation of resilience, collaboration, and capacity building, underscoring their strong commitment to these competencies. However, the results also exposed limited institutional support for responsible research, interdisciplinary engagement, and impact translation, highlighting urgent developmental needs and the necessity for structured, context-sensitive training embedded in doctoral and early-career development programs.

**Conclusion:**

By embedding care, sustainability, and justice at its core, upSkill.Map transcends traditional competency frameworks to offer both evaluative and developmental capabilities. It empowers researchers to prioritize competencies that build rigorous, ethically grounded, and socially responsive research cultures while advancing capacity aligned with UN Sustainable Development Goals. Beyond individual reflection, the instrument provides institutions and policymakers with a scalable mechanism to strengthen researcher development, implement context-sensitive training, and accelerate systemic transformation toward sustainable, equitable, and impactful research ecosystems.

## 1. Introduction

We live in an era of escalating global challenges that includes climate breakdown, widening social inequalities, geopolitical instability, and technological disruption. Today's researchers are increasingly expected to contribute not only disciplinary knowledge but also solutions that are relevant, sustainable, and transformational for the future. This expanded role demands transversal competencies ( ESCO, 2025) such as systems thinking, intersectoral communication, transdisciplinary collaboration, ethical reasoning, and a deep commitment to both planetary and public good.

Several research competencies frameworks have emerged recently. Among them, the European Commission's ResearchComp Framework ( European Commission, 2025) presents a structured matrix of transversal competences applicable across research careers. It addresses areas such as communication, collaboration, and self-organisation, mapping them to learning outcomes and proficiency levels. Similarly, Vitae (2025)'s Researcher Development Framework (RDF) and the OECD (2019)'s Core Competency Framework prioritise education for employability and generic skills development.

However, these frameworks reveal a fundamental limitation in how researcher competence is conceptualized. Despite their strengths, ResearchComp lacks clear orientation toward responsible, mission-driven research that addresses societal and global priorities and generates long-term value for the public and the planet. While it mentions "sustainable"—primarily in relation to plan self-organisation (personal time management and environmental awareness)—it omits substantive references to equity, justice, and the broader societal role of research. The RDF and OECD frameworks similarly embed sustainability, equity, or social transformation only peripherally, if at all.

A Paradigmatic Shift in Researcher Development. These limitations reflect a deeper paradigmatic issue: existing competency frameworks treat ethics, sustainability, and justice as supplementary skills to be 'added on' to technical capabilities. This approach assumes that research competence is primarily technical, with values-based considerations as secondary. upSkill.Map challenges this paradigm by positioning care, responsibility, and planetary consciousness as foundational to research identity—not optional extras. This represents a fundamental reorientation from competency-as-performance toward competency-as-worldview, where researchers' technical skills are inseparable from their ethical commitments and societal purpose. The CARE-KNOW-DO model operationalizes this integration by requiring researchers to ground their knowing and doing in explicit caring—a radical departure from frameworks that separate technical proficiency from moral engagement.

As Smith (2018) notes, existing assessment tools often lack the nuance to support critical reflection on values, purpose, and researchers' societal impact. Taylor and Clark (2023) add that while awareness of transversal skills is growing, their curricular integration remains uneven due to institutional inertia and disciplinary silos.

"Transversal knowledge, skills and competences are relevant to a broad range of occupations and economic sectors. They are often referred to as core skills, basic skills or soft skills, the cornerstone for the personal development of a person. Transversal knowledge, skills and competences are the building blocks for the development of the 'hard' skills and competences required to succeed on the labour market." ( ESCO, 2025)

Lee *et al.* (2022) show that competencies such as leadership and project management improve researchers' career satisfaction and cross-sector success. Garcia *et al.* (2017) and Patel *et al.* (2020) demonstrate the power of project-based and experiential learning in fostering ethical engagement, collaboration, and resilience. Yet they also underscore the need for validated, context-sensitive instruments that not only support skill acquisition but also encourage critical, future-facing reflection.

There is a widely held belief that research should be ethically sound, critically rigorous, and relevant to the common good. Yet what is assumed to be obvious often becomes invisible—and therefore ignored or undervalued. Future-oriented researchers should become deeply aware that time, resources, effort, and momentum must be allocated to what most matters for them and the globe at early stages in their careers. The more researchers are connected and committed to the issues they choose to care for in their research—especially those that affect their communities and the planet—the more deeply they will engage with meaningful fields of knowledge, oriented toward innovative solutions and transformative actions. To care, in this context, is not just a starting point, but a continuous attitude that nurtures knowing and guides doing. Research grounded in care awakens a genuine sense of purpose, becoming truly valuable in shaping agents of transformation capable of driving meaningful change.

## 2. Phase 1 – Development of the upSkill.Map instrument

### 2.1 Theoretical foundations

The upSkill.Map instrument is underpinned by six complementary theoretical principles from diverse theories that together provide a robust and values-led foundation for researcher education and development ( Table 1). These principles are distinct but mutually reinforcing, addressing metacognition, competence, transformation, responsibility, sustainability, and equity.

**Table 1.** Theoretical Frameworks and Their Contribution to *upSkill.Map*.

Theoretical Principles	Core Concepts	How It Underpins *upSkill.Map*
1. Self-Assessment & SRL	Self-efficacy, goal setting, metacognition	Scaffolds reflective, self-directed growth through prioritisation and rating tools
2. Competency-Based & Developmental Models	Holistic capability, researcher identity, progression	Structures the 8Cs and CARE–KNOW–DO model as developmental pathways
3. Transformative Learning	Critical reflection, perspective shift, identity	Supports values clarification and societal role development
4. RRI	Inclusion, reflexivity, public accountability	Anchors Catalyse and CARE domains in anticipatory, ethical engagement
5. Sustainability Science	Systems thinking, action competence	Aligns development with SDGs and future- focused interdisciplinary research
6. Equity-Oriented Development	Capabilities, justice, wellbeing	Promotes inclusion, supports marginalised researchers, challenges deficit models

***1. Self-Assessment and Self-Regulated Learning***

Self-Assessment Theory ( Hattie & Timperley, 2007) and Self-Regulated Learning ( Zimmerman, 2002; Zimmerman & Schunk, 2011) position learners as active agents in their development. upSkill.Map builds on these by enabling researchers to evaluate their skills, prioritise areas for growth, and reflect on progress over time. This scaffolds self-efficacy, feedback use, and goal-directed monitoring, turning reflection into actionable strategies for professional growth.

***2. Competency-Based and Developmental Models***

Competency-Based Education ( Eraut, 1994; Mulder, 2014) views competence as the integration of knowledge, skills, values, and attitudes in real contexts. Developmental frameworks such as Vitae RDF, UniWiND, DIONE, and the Dreyfus model (1986) highlight progression from novice to expert. upSkill.Map incorporates both, combining staged ratings with the CARE–KNOW–DO model:

- CARE: values and engagement (Communicate, Collaborate, Cultivate)
- KNOW: knowledge and analysis (Comprehend, Construct, Coordinate)
- DO: application and impact (Create, Catalyse).

This design integrates motivation, understanding, and practice into a coherent researcher identity.

***3. Transformative Learning***

Transformative Learning Theory ( Chen *et al.*, 2020; Cranton, 2006; Mezirow, 1997) emphasises reflection and perspective change. upSkill.Map incorporates this by embedding indicators of justice, equity, and sustainability, encouraging researchers to question assumptions and align professional identities with wider societal commitments.

***4. Responsible Research and Innovation (RRI)***

RRI ( Stilgoe *et al.*, 2013; von Schomberg, 2013) reframes research as anticipatory, reflexive, inclusive, and responsive. upSkill.Map operationalises these dimensions by encouraging open science, stakeholder engagement, and ethically informed decision-making. RRI thus provides both an ethical compass and a practical framework for aligning innovation with societal needs.

***5. Sustainability Science***

Sustainability Science ( Langston, 2021; UNESCO, 2017; Wiek *et al.*, 2011) identifies competencies such as systems thinking, action competence, and future literacy. These inform the instrument’s emphasis on interdisciplinarity, scenario planning, and readiness to act for sustainable change, equipping researchers to address complex, interconnected global challenges.

***6. Equity-Oriented Models of Development***

Equity-focused perspectives ( McAlpine & Amundsen, 2018; Raffaghelli *et al.*, 2020; Walker & Boni, 2006) emphasise epistemic justice, wellbeing, and the inclusion of diverse voices. upSkill.Map incorporates these by valuing plural knowledge systems, supporting underrepresented groups, and challenging deficit models of researcher development.

Together, these six principles balance individual agency with collective responsibility, technical capability with ethical purpose, and developmental progression with transformative change ( Abdelghaffar & Eid, 2025; Brown *et al.*, 2024).

### 2.2 Instrument constructs

upSkill.Map translates these theoretical principles into a practical developmental self-assessment instrument. It is organised around the 8Cs Framework ( Table 2), aligned with the CARE–KNOW–DO model. The framework specifies eight domains of capability, each defined by four indicators (32 in total). Respondents rate each indicator on a four-point scale (very low to high), then prioritise development needs. This design produces both individual profiles and collective patterns of researcher capability.

**Table 2.** Mapping the 8Cs to Theoretical Concepts ( *Superscript numbers refer to Sections 2.1 to 2.6*).

8C Domain	Core Skill Constructs	Key Concepts
**Collaborate** *Support interdisciplinary research* * partnerships*	Interdisciplinary teamwork Team contribution Conflict resolution Inclusive diversity	Metacognition ^1^, Interdisciplinarity ^2^, Inclusion ^6^, Agency ^6^
**Communicate** *Share accessible and intercultural * *knowledge*	Science communication Network building Policy engagement Intercultural dialogue	Feedback use ^1^, Reflexivity ^4^, Perspective shift ^3^
**Cultivate** *Nurture lasting, inclusive professional * *growth*	Mentoring support Community participation Event organisation Reflective Practice	Self-efficacy ^1^, Capabilities approach ^6^, Wellbeing ^6^
**Construct** Build evidence-based, participatory solution	Research design Literature synthesis Methodological innovation Ethical practice	Knowledge integration ^2^, Systems thinking ^5^, Ethics ^5^
**Comprehend** *Critically understand diverse perspectives*	Systems analysis Evidence evaluation Information synthesis Creative problem-solving	Systems thinking ^5^, Critical reflection ^3^, Cognitive regulation ^1^
**Coordinate** *Lead inclusive, engaging, and committed* * teams*	Resource management Priority balancing Risk management Team leadership	Developmental progression ^2^, Leadership ^2^, Anticipation ^4^
**Create** *Develop responsible research and* * innovation*	Interdisciplinary links Innovative thinking Knowledge cocreation Adaptive research	Creativity ^3^, Boundary-crossing ^5^, Innovation competence ^2^
**Catalyse** Drive sustainable change for the common good	Open research Policy innovation Community empowerment Impact evaluation	Public value ^4^, Ethics ^5^, Societal responsibility ^6^

This mapping illustrates how the instrument’s design is not arbitrary but systematically integrates metacognition, progression, transformation, responsibility, sustainability, and equity.

### 2.3 Instrument overview

The instrument integrates qualitative and quantitative data to capture a holistic picture of researcher development.

- **Part 1: Qualitative Experience (Research Impact Involvement)**
  Participants describe their engagement with research impact, aligned with sustainability and global challenges (e.g. SDGs). They select and elaborate on areas such as major grants, contributions to global challenges, policy influence, or other impactful work. This narrative establishes a baseline of lived experience.
- **Part 2: Quantitative (8Cs Skills Self-Assessment)**
  Using the 8Cs Framework, participants rate 32 indicators on relevance to their role and rank their top development priorities. This produces structured self-assessment data, highlighting both individual pathways and collective needs.
- **Part 3: Qualitative Reflection (“Think Piece”)**
  Participants then write a reflective narrative addressing themes such as:
  ○ How training has shaped their skills and purpose, especially for sustainability and public good.
  ○ Preparedness for real-world challenges beyond academia.
  ○ Institutional enablers and constraints.
  ○ Careers and recognition in researcher training.
  ○ Defining successful research leadership today.

Their reflection seeks to enrich the quantitative findings with critical and creative insights.

By integrating these three elements, upSkill.Map links experiential accounts, structured self-assessment, and reflective narratives, offering a comprehensive and developmental resource for researcher education.

## 3. Phase 2 – upSkill.Map methodological application and validation

### 3.1 Research design

This study adopted a design-based research (DBR) approach ( Design-Based Research Collective, 2003) to iteratively develop, implement, and validate the *upSkill.Map* self-assessment tool. DBR was chosen for its capacity to integrate theory and practice in authentic educational contexts, allowing continuous refinement through empirical feedback and stakeholder engagement. Information about the project and the study was embedded within the *upSkill* instrument, and written informed consent for participation was obtained directly through the instrument activities. Ethical approval for the study was granted by The Open University Human Research Ethics Committee.

**Figure 1.**
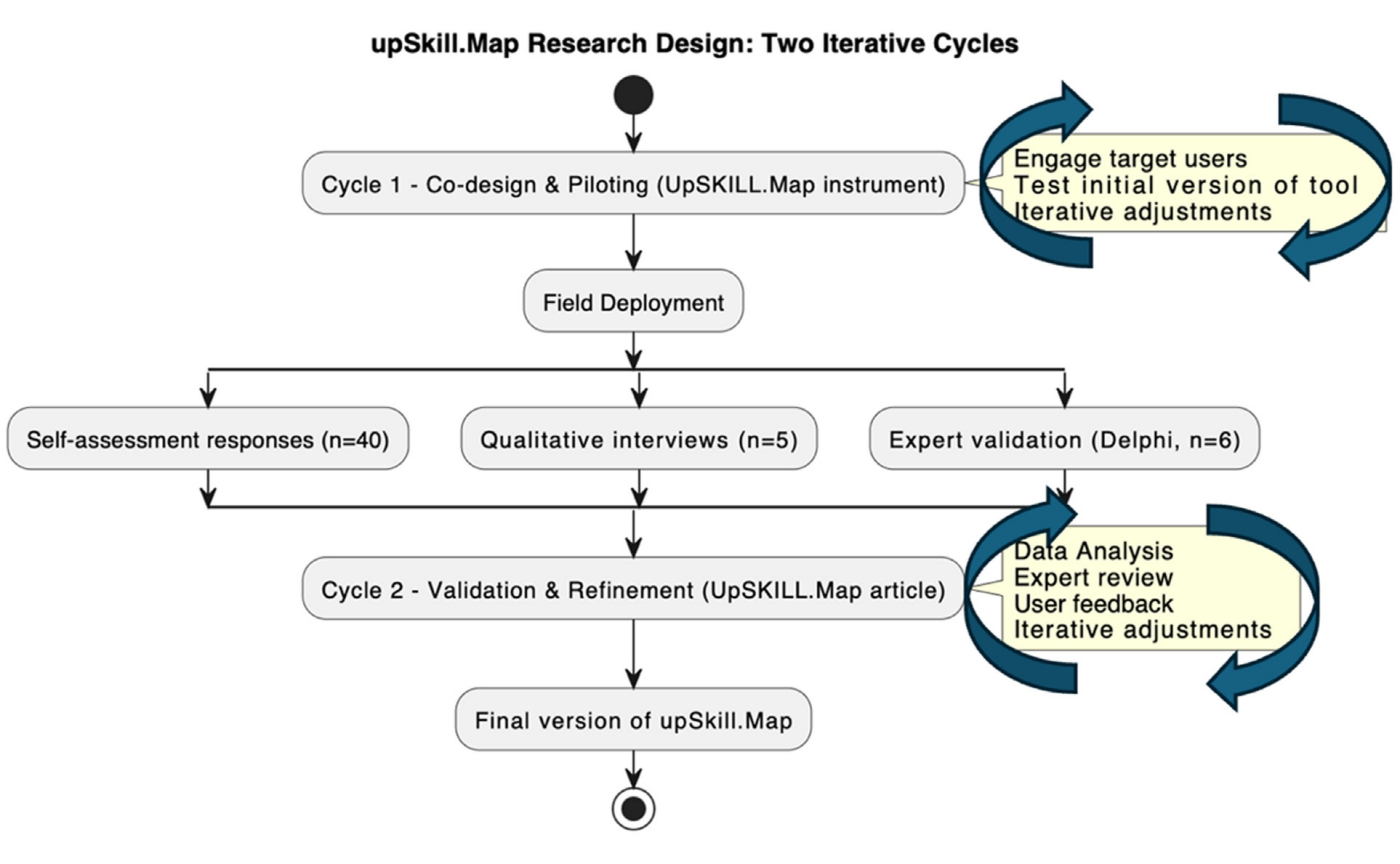
UpSkill.Map Research Design.

The research unfolded in two iterative cycles ( Figure 1):

1. **Piloting (Co-design)** with target users.
2. **Validation (Refinement)** through expert review, and user feedback.

Field Deployment occurred through three complementary methods for data production:

1. **Self-assessment responses** to the *upSkill.Map* tool (n = 40)
2. **Qualitative interviews** with selected participants (n = 5) focusing on usability, relevance, and perceived impact
3. **Expert validation** via a Delphi-style consultation with six experts in researcher development, sustainability, and higher education policy.

The instrument underwent iterative technical adjustments during the pilot phase (prior to field deployment), informed by participant and expert feedback. The final version was then used consistently for all participants during the main data collection (8 May – 8 July 2025). After data collection, additional analytic refinements were applied, based on statistical checks and peer-review feedback.

### 3.2 Participants and sampling

Recruitment began via the Open University’s doctoral programme, with invitations and reminders sent to nearly 500 doctoral students and 100 supervisors, yielding 10 participants (<2%). Additional 30 participants were then recruited voluntarily through purposive and snowball sampling across four doctoral programmes in the OU, supported by our research group and supervisor nominations from the OU’s Open Societal Challenges programme ( Table 3). Demographic indicators were collected to examine potential patterns of inclusivity and diversity in doctoral education. While the main analysis focused on career stage (given its centrality to training pathways), other variables such as ethnicity, gender, disability status, and language background were collected to enable intersectional checks and to situate the findings within broader equity considerations. Not all categories are discussed in depth, but summary distributions are reported for transparency and rigour.

**Table 3.** Participant Demographics *(n = 40)*.

Category	Subcategory	n	%
**Current Career Stage/Role**	Research Student	19	47.5
	Research Associate	3	7.5
	Research Fellow / Lecturer	8	20.0
	Senior Researcher / Senior Lecturer	5	12.5
	Professor / Director	3	7.5
	Other academic role	1	2.5
	Director	1	2.5
**Nationality**	Europe	22	55.0
	Africa	11	27.5
	Other continents (combined)	7	17.5
**Gender**	Female	26	65.0
	Male	13	32.5
	Non-binary	1	2.5
**Age Group**	40–49	13	32.5
	50–59	10	25.0
	30–39	7	17.5
	Other / not stated	10	25.0
**Ethnicity**	White / Caucasian	22	55.0
	African / Black	9	22.5
	Asian	4	10.0
	Mixed / multiple ethnicities	3	7.5
	Other / not stated	2	5.0
**Disability / Accessibility Needs**	Yes	7	17.5
**Disciplinary Area**	Wellbeing, education, language	15	37.5
	STEM	8	20.0
	Arts / social sciences	7	17.5
	Business / marketing	6	15.0
	Other / not stated	4	10.0
**Career Aspirations**	Academic progression	17	43.0
	Professional / industry	12	30.0
	Senior research management	7	17.5
	Other	4	10.0

The study drew on a relatively small, primarily volunteer sample (n = 40) recruited from specific doctoral programmes, which may not fully represent the broader population of doctoral researchers. Participation was likely influenced by motivation, digital literacy, or engagement with eco-outwards practices, introducing potential self-selection bias. These characteristics should be considered when interpreting both the findings and the broader applicability of the upSkill.Map instrument, which, while designed for wider use, would benefit from further testing with more diverse and larger samples.

### 3.3 Data analysis

An *explanatory sequential mixed-methods* design (quantitative → qualitative) was used.

The quantitative stage employed descriptive statistics to summarise participant characteristics and exploratory factor analysis (EFA) to identify latent clusters of research skills. EFA was selected as an inductive approach to uncover coherent domains without imposing a priori structures, providing an empirically grounded view of the skill landscape.

The qualitative stage centred on thematic analysis of open-ended “think-piece” reflections and linked prompts. These invited participants to articulate personal experiences and perspectives on how research training supports skill development, readiness for real-world challenges, and alignment with sustainability, equity, and the public good. Additional reflections explored institutional enablers and constraints, the recognition of researcher careers, and evolving definitions of research leadership. Closing questions encouraged participants to propose changes that could enhance the inclusivity, relevance, and transformative impact of research training.

To contextualise these insights, participants also indicated whether they were engaged in research impact activities (e.g., contributing to the Sustainable Development Goals, advancing knowledge on global challenges, influencing policy or regulation, or pursuing other significant pathways). These accounts provided depth on the applicability of the skill domains and their translation into practice. The analysis was further enriched through triangulation with expert review and written user feedback, which informed refinements to construct validity and usability of the upSkill.Map.

Finally, quantitative and qualitative findings were integrated through joint displays, visually aligning statistical trends with participants’ lived experiences. From this integration, meta-inferences were drawn to generate practical recommendations for curriculum development and research policy, guided by a commitment to sustainability, fair societies, and the common good.

### 3.4 Trustworthiness and limitations

Rigour was ensured through three strategies. First, triangulation combined participant reflections, expert review, and the theoretical foundations mapped in the initial study phase, helping to cross-validate findings and reduce single-source bias. Second, iterative design-based research (DBR) cycles of piloting and refinement enhanced credibility, dependability, and confirmability ( Lincoln & Guba, 1985). Third, grounding the tool in international frameworks—the EU Research Competence Framework (RMComp; EC, 2025) and the United Nations Sustainable Development Goals—strengthened both conceptual validity and applicability across diverse research contexts.

Several limitations should be noted; alongside opportunities they open. The sample size was modest, which restricts the generalisability of findings, yet it included participants from across the university spectrum, bringing disciplinary diversity and a breadth of perspectives. The study relied on self-reported data, which carries a risk of social desirability bias. However, this was mitigated by the voluntary and open-ended design of the think-piece questions, which allowed participants to express in-depth views in their own terms. Finally, while the study captured a range of nationalities, the focus on one institutional setting limited the cross-cultural scope. At the same time, the diversity of experiences and interests reported provides a strong foundation for extending the work to other contexts.

Future research should therefore prioritise longitudinal validation to track developmental trajectories of competencies, broaden participation across institutions and regions to strengthen cultural relevance, and explore integration of the upSkill.Map into doctoral training policy and institutional evaluation frameworks. Such steps would enhance its potential to drive systemic change towards more sustainable, equitable, and socially responsive research cultures.

## 4. Findings

### Research on global challenges

Participants were asked to indicate whether they were currently engaged in research impact activities, such as contributing to the Sustainable Development Goals (SDGs), advancing knowledge to address global challenges, influencing policy or regulation, or pursuing other significant pathways (see questionnaire in Data Availability section).

Engagement with impact was reported across a range of contexts. While all participants were based within a single UK university, the settings in which they carried out their research were global, spanning Sub-Saharan Africa, Europe, Latin America, and Asia. Examples included inclusive literacy strategies in Africa, participatory climate heritage projects in Europe, assistive AI tools for migrants in Latin America, and genomic data-sharing for public health in Asia.

In total, 40 participants contributed responses. This represents a small but diverse cohort, reflecting the range of disciplinary areas within the institution. Participants identified six main mechanisms of research impact ( Table 4): advancing knowledge to address global challenges (53.7%), creating social innovation products (51.2%), shaping policy and regulation (41.5%), contributing to major grant-funded initiatives (36.6%), and, to a lesser extent, other areas (17.1%) or none of the above (19.5%). More than half described innovating beyond traditional research outputs, and over a third were engaged in externally funded projects.

**Table 4.** Mechanisms of research impact.

Impact Mechanism	% (n=40)	Example Projects/Themes	No. Research Students (out of 23)
(1) Advancing knowledge to address global challenges	22/40 = 55.0%	Disaster risk reduction; decolonising death & grief studies; OpenSTEM Africa; genetics; climate change education; sustainability reporting	13
(2) Social innovation products	21/40 = 52.5%	AI apps for migrants; dialogic teaching; digital/accessible platforms; teacher development; inclusive STEM	10
(3) Shaping policy or regulation	17/40 = 42.5%	Workplace reproductive rights; DfE/Welsh policy; democracy; transport accessibility; AI/education regulation	7
(4) Major grant-funded initiatives	15/40 = 37.5%	CONNECT 2030 (EU); GCRF/British Academy; UKRI Future Leaders; Fleming Fund; Horizon Europe	8
(5) Other	7/40 = 17.5%	Ethics in digital design; green entrepreneurship; curriculum coding/ computational thinking; teaching professional development	3
(6) None of the above	8/40 = 20.0%	(No current involvement in above mechanisms)	7

Although situated within one institution, the diversity of participants’ fields, regions of research practice, and thematic commitments provides insight into how researchers navigate impact at the intersection of academic rigour and social relevance. Cross-cutting themes included digital transformation, inclusive development, co-creation with communities, and open innovation for sustainable change. Most projects were aligned with the SDGs—particularly SDG 4 (Quality Education), SDG 10 (Reduced Inequalities), and SDG 17 (Partnerships for the Goals)—underscoring the strong outward-facing orientation of the sample.

These findings highlight both the strengths and limitations of the study. On the one hand, the modest, single-institution sample size restricts generalisability. On the other, the breadth of disciplines, nationalities, and research interests represented suggests that the insights are transferable to comparable higher education contexts, especially those aiming to integrate equity-driven and sustainability-oriented approaches into researcher training and policy.

### Descriptive statistics

Participants rated the relevance of a range of research-related skills to their current roles using a 1–4 scale (very low, low, moderate, high). The most highly valued skills were critically evaluating evidence (M = 3.85), applying ethical principles (M = 3.75), building professional networks (M = 3.60), and synthesising information from diverse sources (M = 3.58), underscoring the centrality of analytical reasoning, ethics, and effective communication in their work.

Most skills scored above moderate relevance (M > 3.0), indicating broad alignment with participants’ daily responsibilities. However, some competencies—including engaging policymakers (M = 2.95), leading research teams (M = 2.88), and practising intercultural communication (M = 2.77)—were seen as less relevant, with greater variation across responses. This points to uneven applicability depending on role and suggests a need for more targeted development in leadership, policy engagement, and cross-cultural skills to fully support diverse career pathways.

### Item-level correlation and scale coherence

The item-level Pearson correlation analysis for the 8C skill set (N = 40; 28 items) reveals consistently strong within-domain correlations, particularly in clusters such as collaboration and communication (r ≈ 0.65–0.85). For instance, “Working in teams across disciplinary boundaries” and “Contributing to develop a team project” exhibited a correlation of approximately 0.80, confirming the internal coherence of these constructs.

Moderate to high cross-domain correlations (r ≈ 0.40–0.70) were also observed, suggesting that research-related competencies are perceived holistically. This pattern reinforces the construct validity of the instrument, capturing integrated professional skillsets. While several inter-item correlations exceeded 0.75, indicating a high degree of internal consistency (α = 0.96), this also flags a minor risk of redundancy—an acceptable trade-off in early-stage tool validation where content breadth is essential.

### Group comparisons by career stage

Mann–Whitney U tests comparing Research Students (n = 19) with Research Fellows or Lecturers (n = 10), Senior Researchers/Senior Lecturers (n = 5), and Professors/Directors (n = 3) showed no statistically significant differences across any of the 28 skill items. All p-values exceeded 0.10, with most above 0.25, including those for items with the largest observed group mean differences (e.g., “Building and maintaining professional networks”, p > 0.13).

This consistent non-significance suggests a stable consensus across career stages regarding the relevance of research skills. The absence of divergence in skill perception strengthens the argument for applying the 8C framework universally across roles in research training contexts, supporting its construct validity and practical utility. The 8C skill framework demonstrates both psychometric robustness and practical alignment across diverse academic roles. The lack of significant differences between Research Students and more senior academics supports the cross-stage validity of the scale and highlights its potential to inform inclusive, whole-career research capacity-building strategies.

### Exploratory factorial analysis: sample size and statistical considerations

**Sample Size and Statistical Considerations.** The current sample size (n=40) falls below conventional recommendations for exploratory factor analysis (typically 5–10 participants per variable). However, several indicators support the validity of our approach: the Kaiser-Meyer-Olkin measure (0.732) and significant Bartlett's test (χ²(378) = 1152.934, p < .001) confirm data suitability, while excellent internal consistency (α = 0.963) suggests robust underlying structure. Our design-based research approach positions this as preliminary validation to inform larger-scale studies, with parallel analysis and cross-validation techniques employed to enhance confidence in the extracted factors.

**Data Preparation and Analysis.** Four variables were removed prior to analysis due to low correlations, poor communalities, and inadequate measures of sampling adequacy. These items (designing research methodologies, analysing complex systems, generating creative solutions, and balancing competing demands) were either too abstract or poorly aligned with the 8Cs framework, and their removal improved overall reliability and factor clarity.

**Figure 2.**
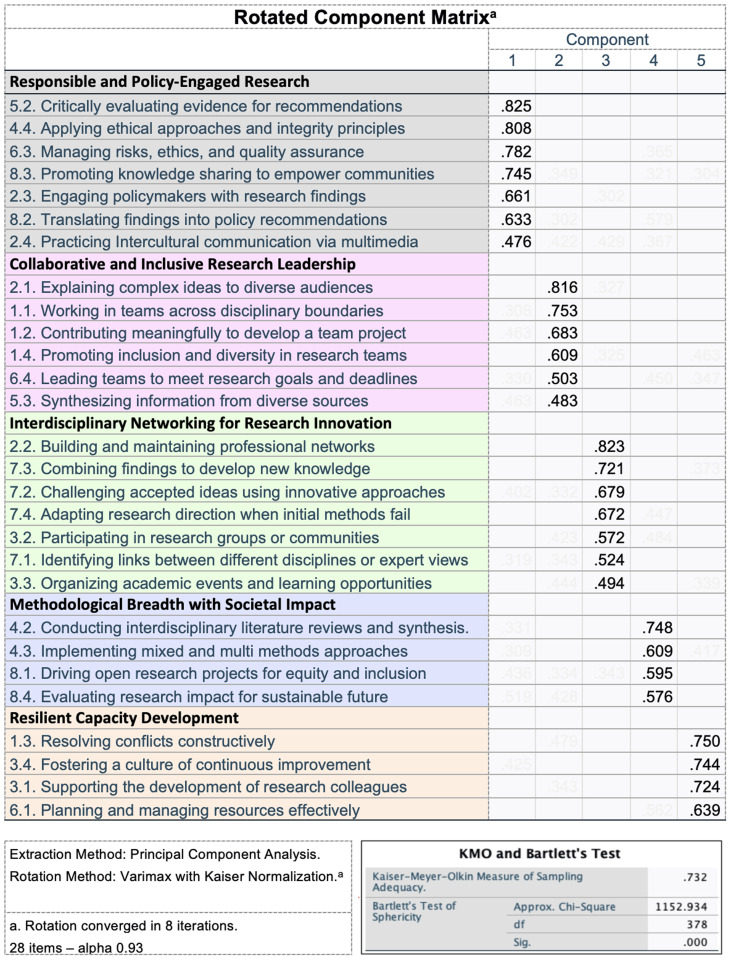
Exploratory factor analysis revealing 5 clusters integrating 8 C skills.

**Factor Extraction.** Exploratory factor analysis employed Varimax rotation ( Figure 2) to produce orthogonal factors, chosen based on preliminary checks indicating low inter-factor correlations. The rotation converged in 8 iterations, yielding a stable five-factor structure that demonstrates robust psychometric properties for measuring research competencies across diverse dimensions.

Five factors were identified through principal component extraction with varimax rotation, and thematic analysis of qualitative data was used to illustrate each cluster. This structure provides an empirically grounded framework for analysing role-based differences and informing targeted capacity-building interventions across the research career pathway.

## Mixed methods analysis

Our mixed-methods study adopts an applied analytical approach, integrating findings with targeted recommendations to support policy and practice. By linking evidence directly to recommendations, we aim to provide actionable insights. Quantitative data confirm all five factors scored above the midpoint. Factor-1 received the highest ratings, Factor-5 the lowest. This pattern suggests researchers possess strong ethical awareness but are less equipped to sustain their work in complex contexts.

The findings presented here relate to the sample in this study and should be read in that context. While the small-scale sample does not claim statistical generalisability across the sector, the patterns observed illustrate how the instrument can surface skill dynamics that are likely to be relevant in comparable settings. The wider significance lies in modelling how the upSkill.Map can be applied by other institutions to guide research training design and evaluation

### Factor 1: Responsible and Policy-Engaged Research

EFA revealed a cluster of seven interrelated skills: critical evaluation, ethical principles, risk management, community empowerment, policymaker engagement, evidence-to-policy translation, and multimodal intercultural communication. This construct integrates research integrity with strategic engagement to consolidate societal impact. While valued by both early-career and senior researchers, qualitative evidence suggests these commitments remain largely aspirational, as structural constraints hinder consistent practice.

Thematic analysis of open-ended responses ( Box 1) captured five perspectives from students, researchers, and academic leaders. A recurring theme was the *aspirational–practical gap*: the tension between strong commitments to responsible research and the real-world challenges of implementation. Many students expressed motivation to pursue societal impact, ethical integrity, and policy relevance—echoing the skills identified in this factor. For example, they called for greater industry collaboration, critical engagement with emerging ethical issues such as AI, and research that delivers tangible long-term benefits.

**Box 1. Thematic analysis linked to Factor-1 Responsible and Policy-Engaged Research**

**Table:** 

Themes	Most Representative Quote	Stakeholder
1. Aspirational vs. Practical Gap	“I have always struggled to align my work with the public good or sustainability as it’s been so ‘blue skies’.”	Leadership [L5]
2. Skills and Training Deficits	“Structured training in leadership or policy engagement is more limited… others lack the mentoring or applied contexts to do so confidently.”	Research Student [DS1]
3. Institutional Constraints	“Without external funding or pre-established relationships… it is very difficult to pursue real world social impact.”	Research Fellow [R11]
4. Ethical Imperative	“When research on inclusion does not translate into real-world impact, it contributes to marginalisation and discrimination.”	Research Student [DS6]
5. Systemic Change Needed	“Embed real-world engagement into the core of doctoral education not as an add-on, but as a fundamental part of research design.”	Research Student [DS5]

However, students frequently reported insufficient training and limited opportunities to develop the skills needed to translate research into policy or community outcomes ( *Skills and Training Deficits, Theme 2*). Across stakeholder groups, there was consensus on the importance of societal benefit and research integrity, yet frustrations emerged regarding institutional barriers such as restricted funding and rigid structures that inhibit applied engagement ( *Institutional Constraints, Theme 3*). Senior researchers emphasised the ethical imperative to ensure research contributes beyond academia ( *Ethical Imperative, Theme 4*), while calls for reform highlighted the need to embed real-world engagement more centrally within doctoral education ( *Systemic Change Needed, Theme 5*).

These insights highlight the urgent need for strategies to bridge the gap between aspiration and practice. Prioritising opportunities for students and early-career researchers to engage in real-world translation, policy dialogue, and community collaboration can help build the skills captured in Factor 1. Equally, institutional reform—addressing funding limitations and rigid structures—is essential to create an enabling environment where responsible, impactful research is systematically supported.

### Factor 2: Collaborative and Inclusive Research Leadership

EFA identified a cluster of six interconnected skills centred on the ability to communicate complex ideas to diverse audiences, contribute meaningfully within research teams, and foster inclusive environments. These interpersonal and organisational competencies are critical for enabling productive and equitable collaboration in research contexts. Factor 2 received consistently high ratings, especially from students and lecturers, indicating that inclusive practices are both recognised and valued. However, participants also reported limited time for reflection, unequal decision-making power, and a lack of recognition for collaboration in promotion systems—echoing calls for systemic change to embed and reward inclusive leadership.

Thematic analysis ( Box 2) reinforced the centrality of these competencies but revealed gaps in practice. Students placed high importance on communication and peer networks (Theme 1), yet described persistent collaborative skills gaps (Theme 2) and institutional recognition barriers (Theme 3) that undermine their ability to contribute fully. Aspirations for embedding inclusion and sustainability into research design (Theme 4) often remained unfulfilled, while wellbeing through connection (Theme 5) was seen as both a personal necessity and an institutional responsibility.

**Box 2. Thematic analysis linked to Factor-2 Collaborative and Inclusive Research Leadership**

**Table:** 

Themes	Most Representative Quote	Stakeholder
1. Communication and Peer Networks	"Communication and collaboration. These are essential not only for effective teamwork but also for translating research into meaningful outcomes and engaging with diverse stakeholders."	Research Fellow (R8)
2. Collaborative Skills Gaps	"We also need more practical training - hands-on training... Skills such as research and analytical skills, academic writing and communication, time and project management, networking and collaboration."	Research Student (DS3)
3. Institutional Recognition Barriers	"There is currently no way to account for how individual researchers and the ways they think impact their lab. I have a colleague who has contributed to nearly every research group informally, but she is not valued."	Senior Researcher (SR4)
4. Inclusive Practice Aspirations	"Embed inclusion and sustainability into the structure of research design—not as optional themes, but as core values. A successful doctorate today should not only advance knowledge but make that knowledge usable, accessible, and accountable to wider society."	Research Student (DS1)
5. Wellbeing Through Connection	"One important point we haven't discussed is mental health and well-being. Many students face stress, isolation, or uncertainty during their PhD... Institutions should prioritise well-being support, foster peer communities, and normalise help-seeking."	Research Student (DS2

Overall, these findings suggest that while collaborative leadership and inclusive communication are widely endorsed, they are unevenly developed and insufficiently supported in formal training. Informal networks partially address these gaps, but systemic changes to training structures, evaluation criteria, and institutional culture are needed to embed these competencies more consistently in research practice.

### Factor 3: Interdisciplinary Networking for Research Innovation

Exploratory Factor Analysis (EFA) identified a cluster of capabilities combining creative problem-solving, adaptability, and the sustained relationship-building needed to advance research agendas in complex and evolving contexts. These skills underpin cross-disciplinary collaboration and the ability to navigate emerging challenges. Factor 3 received moderate scores overall, with senior researchers rating these competencies more highly than students, likely reflecting experience-based confidence. Students reported restricted access to networks and difficulties engaging in intersectoral collaboration, underscoring the need for institutional strategies that democratise access to professional communities and innovation spaces.

Thematic analysis of qualitative responses ( Box 3) revealed strong proactive engagement with innovation and networking among students and early career researchers (ECRs). Activities included integrating AI and other digital tools into research workflows, building cross-disciplinary partnerships, organising academic events, and connecting with both academic and non-academic stakeholders. There was consistent demand for structured, credit-bearing opportunities in areas such as grant writing, professional exchanges, and industry or NGO collaborations, with a view to producing tangible and demonstrable outcomes.

**Box 3. Thematic analysis linked to Factor-3 Interdisciplinary Networking for Research Innovation**

**Table:** 

Theme	Most Representative Quote	Stakeholder
AI Integration and Digital Innovation	"Yes, I integrate AI tools into my research to enhance productivity, deepen analysis, organize data, and improve text quality. I use tools such as ChatGPT, Grammarly, Notion AI, Slack, Trello, Padlet, Copilot, Consensus, IRaMuTeQ, among others."	Research Fellow (R8)
Cross-Disciplinary Collaboration	"Skills: create research outputs (e.g. workflows, datasets); communicate (e.g. writing tutorials and articles); collaborate (working with biologists and computer scientists)."	Research Student (DS13)
Professional Network Building	"Skills such as research and analytical skills, academic writing and communication, time and project management, networking and collaboration, teaching and presentation skills, and adaptability and career planning are key for doctoral completers."	Research Student (DS3)
Grant Writing and Funding	"More hard skills (technologies, methodologies, techniques); a set of tangible outcomes beyond passing a viva and a thesis: publishing paper(s), grant writing, project management, budgeting, teaching duties, running conferences."	Research Fellow (R7)
Industry and Real-World Partnerships	"Universities can better support career development by embedding practical training, creating stronger networks with industry and NGOs, and recognising non-traditional pathways as valid. PhD research can have tremendous societal impact when aligned with urgent challenges."	Research Associate (R1)
Creative Problem-Solving	"I develop innovative lithium separation technologies, so Creating and Collaborating are the main 8C skills... training opportunities and support from my line manager has been crucial to learn and thrive in this new research area."	Research Associate (R3)
Event Organization and Leadership	"I also contributed to the organisation of a large-scale conference which led to organising a post-graduate conference at the institution – all of which developed leadership skills and experience, offering a useful skillset within academia and beyond."	Research Fellow (R4)
International and Global Networking	"Having the opportunity to conduct part of my doctoral programme abroad was transformative. It gave me a global perspective on education and encouraged me to engage more deeply with international debates on global issues."	Research Fellow (R8)

Senior researchers emphasised the importance of sustained interdisciplinary dialogue and regular exchanges across departments, while institutional leaders focused on the strategic cultivation of external networks and the careful management of risks associated with openness in a challenging global climate, particularly regarding equity, diversity, and inclusion (EDI).

Overall, these findings indicate that while researchers at all stages recognise the value of innovation and networking, opportunities to develop these capabilities are often self-initiated or externally driven rather than embedded in core doctoral training. Embedding structured, well-supported pathways for professionalisation—paired with formal recognition of achievements—would strengthen capacity for innovation, adaptability, and engagement across career stages.

### Factor 4: Methodological Breadth with Societal Impact

EFA identified a cluster representing methodological breadth—ranging from literature synthesis to mixed-methods application—with a strong emphasis on equity, inclusion, and sustainability. This construct links interdisciplinary inquiry with tangible societal and environmental benefits, underscoring the potential of research to achieve meaningful impacts beyond academia.

Factor 4 scored well overall, yet findings revealed a persistent gap between the value placed on these skills and their consistent application in practice. Disciplinary silos, insufficient incentives, and the complexity of evaluating impact remain significant barriers, reflecting Horizon Europe’s observation that interdisciplinarity is essential yet under-supported ( European Commission, 2023).

Thematic analysis of qualitative responses ( Box 4) revealed widespread ambition—particularly among students—to engage in interdisciplinary work that transcends traditional boundaries. However, barriers emerge at multiple levels: students encounter siloed training and limited exposure to cross-field collaboration; mid-career researchers face workload imbalance and resource constraints; and senior academics point to the absence of institutional structures that systematically enable interdisciplinary initiatives.

**Box 4. Thematic analysis linked to Factor-4 Methodological Breadth with Societal Impact**

**Table:** 

Theme	Most Representative Quote	Stakeholder
Mixed-Methods and Methodological Breadth	"Research training has supported my development of ethical and innovative research by introducing the multiple methods by which research can be participant-led with rich, multisensory, mixed-methods approaches."	Research Student (DS19)
Equity, Inclusion, and Sustainability Focus	"If I could change one thing about doctoral education, it would be to embed inclusion and sustainability into the structure of research design—not as optional themes, but as core values. A successful doctorate today should not only advance knowledge but make that knowledge usable, accessible, and accountable to wider society."	Research Student (DS1)
Cross-Disciplinary Collaboration	"Skills: create research outputs (e.g. workflows, datasets); communicate (e.g. writing tutorials and articles); collaborate (working with biologists and computer scientists) ... I occasionally use AI-based bioinformatics tools to explore datasets."	Research Student (DS13)
Real-World Impact and Societal Benefits	"It's frustrating sometimes, because I believe our research has so much potential to inform policy or support communities, but the academic system doesn't always encourage or reward that kind of engagement. The skills I've found most essential aren't just academic ones... being able to adapt, work ethically, and connect with people across disciplines feels more important than ever."	Research Student (DS5)
Overcoming Disciplinary Silos	"I think the silo of disciplines contributes to this, particularly in AI, which has an enormous engineering focus. I think it's a barrier for social innovation required to address societal challenges and global needs."	Senior Researcher (SR4)
Literature Synthesis and Knowledge Integration	"Yes, I use AI tools primarily for literature reviews and marketing research. For example, I used Scite.ai and Connected Papers to map the evidence base around green entrepreneurship education, and tools like ChatGPT for drafting survey instruments and analyzing open responses."	Research Associate (R1)
Environmental and Social Impact	"Universities can better support career development by embedding practical training, creating stronger networks with industry and NGOs, and recognising non-traditional pathways as valid. PhD research can have tremendous societal impact when aligned with urgent challenges like climate change and youth unemployment."	Research Associate (R1)
Interdisciplinary Team Experiences	"Having the opportunities to work collaboratively with other doctoral students and higher education colleagues throughout my doctoral studies was key in developing transferable skills such as teamwork, leadership and time management."	Research Fellow (R4)

Across stakeholders, there was clear recognition of the importance of embedding inclusion and sustainability into research design, strengthening cross-disciplinary collaboration, and ensuring research outputs deliver real-world benefits. Participants emphasised the value of diverse team experiences, internships, and industry or NGO partnerships to bridge disciplinary divides and enhance impact.

Overall, the findings suggest a shared commitment to interdisciplinary, socially responsible research, but also a structural deficit in the conditions needed for it to thrive. Addressing entrenched silos, funding gaps, and the lack of formalised pathways for collaboration is essential for enabling researchers to fully realise the societal and environmental potential of research competencies.

### Factor 5: Resilient Capacity Development

EFA identified a cluster of adaptive capacities essential for overcoming challenges, sustaining continuous improvement, and supporting colleagues. This construct spans time and resource management, conflict resolution, adaptability under uncertainty, and building a supportive research culture. Factor 5 was the lowest-scoring cluster, particularly among early career researchers, reflecting a gap between the acknowledged importance of resilience and the structured opportunities available to develop it. Many participants reported feeling unprepared for key skills—especially conflict resolution and resource optimisation—which are often acquired informally and late in a career, increasing the risk of burnout and attrition.

Thematic analysis ( Box 5) revealed that resilience manifests differently across career stages. Students and early-career researchers often rely on self-motivation and informal networks to navigate stress, isolation, and limited resources, describing their doctoral journey as largely self-directed. Mid-career researchers emphasised mentorship as critical to sustaining growth and meeting evolving demands, while senior academics underscored the need for adaptability in a changing higher education landscape, yet noted persistent gaps in formal training provision.

**Box 5. Thematic analysis linked to Factor-5 Resilient Capacity Development**

**Table:** 

Theme	Most Representative Quote	Stakeholder
Managing Stress and Self-Doubt	"One important point we haven't discussed is mental health and well-being. Many students face stress, isolation, or uncertainty during their PhD, especially in distance or part-time models. Institutions should prioritise well-being support, foster peer communities, and normalise help-seeking as part of healthy research culture."	Research Student (DS2)
Time Management and Resource Optimization	"Time constraints of a 3-year funded phd means we have limited options in terms fitting every together; that is ensuring we complete in time puts a mental strain on us and our focus is usually also limited to finishing up the thesis."	Research Student (DS4)
Conflict Resolution and Problem-Solving	"Personal drive and perseverance are most important because without this overcoming endless barriers is impossible... Only based on my own experience, academia is often very far removed from the real-world so impact can be a worthy aspiration but requires real-world experience to achieve."	Research Student (DS8)
Self-Motivation and Self-Direction	"My research training has been a variable experience, predominantly self-driven which has made it challenging on occasion to know which path to follow or what to prioritise. Focusing on areas I have some knowledge of, and expanding on these has been most impactful."	Research Student (DS8)
Mentorship and Peer Support	"I was fortunate to have supervisors who truly believed in my potential and consistently reminded me that I could exceed expectations through my commitment, organization, focus, and ability to communicate effectively... This kind of mentorship and inclusion in real, ongoing work developed in me a level of understanding that no formal program could have offered on its own."	Research Fellow (R8)
Adaptability Under Uncertainty	"A successful researcher needs to be flexible, resilient and open-minded... Researchers have the skills; however, we are constrained by the current UK HE environment, which is downwardly focused, contracting and in real trouble."	Leadership (L5)
Sustaining Long-Term Performance	"More ongoing support rather than little nibbles of short things. I wish I could still access vitae for example.... One that can make a living and make a difference... I wish I felt more able to get a research job as this is proving hard."	Research Associate (R2)
Building Supportive Research Culture	"Students and their supervisors are a synthesis of ideas, creativity, inspiration, positivity and sheer hard work! Generally, the more the student engages with their studies, the more the supervisor can help and support the student... Supervisors can help the student to take a more long-term view and help to reduce that awful panicky feeling when things go wrong."	Leadership (L4)

Across stakeholders, common threads include the mental strain caused by compressed timeframes, the challenge of maintaining wellbeing under competing pressures, and the need for stronger institutional systems to sustain long-term performance. Participants described resilience as being built not only through personal drive and adaptability, but also through access to supportive supervisors, peer communities, and ongoing professional development. They also linked resilience to the cultivation of a healthy research culture—one that reduces panic during setbacks, normalises help-seeking, and values collaboration alongside individual achievement.

Overall, Factor 5 emerges as a cross-cutting capability that underpins the capacity to persist, adapt, and grow amidst adversity. Strengthening resilience requires moving beyond reliance on individual coping strategies to embed institutional supports—mentorship schemes, conflict resolution training, wellbeing initiatives, and career development pathways—that enable researchers to thrive and contribute to a sustainable, inclusive research environment.

In sum, The EFA revealed a coherent five-construct framework that captures the multifaceted nature of research capability development across career stages. *Responsible and Policy-Engaged Research* underpins the ethical and strategic engagement of research with societal needs, while *Collaborative and Inclusive Research Leadership* provides the interpersonal foundation for equitable, team-based inquiry. *Interdisciplinary Networking for Research Innovation* extends these relational capacities into creative problem-solving and sustained cross-disciplinary connections. *Methodological Breadth with Societal Impact* bridges methodological diversity with a commitment to equity, inclusion, and sustainability imperatives. Finally, *Resilient Capacity Development* operates as a cross-cutting capability, enabling researchers to adapt, persist, and foster growth both personally and within their communities. Together, these constructs form an empirically grounded, career-spanning model for strengthening individual and systemic research capacity, offering a transferable framework for policy, training, and institutional strategy.

## 5. Discussion

### Paradigmatic contribution

Traditional researcher development frameworks often position ethical and justice-oriented skills as supplementary ( Fischer & Wiek, 2022). Our findings challenge that approach by demonstrating that the upSkill.Map instrument successfully embeds sustainability, responsibility, and equity as core competencies rather than add-ons.

### Integration of theoretical framework and empirical structure

While the upSkill.Map was designed around the 8Cs framework for comprehensive coverage, the exploratory factor analysis revealed five empirically-grounded clusters that provide additional insight into how competencies group in practice. Rather than replacing the 8Cs structure, these factors offer a complementary analytical lens: the 8Cs framework remains pedagogically valuable for reflection and curriculum design, ensuring systematic coverage of research capabilities, while the 5-factor structure reveals natural competency clusters for assessment and professional development.

The following five factors integrate ethics, inclusion, and social responsibility across all domains as fundamental rather than supplementary capabilities:

1. **Responsible and Policy-Engaged Research** ensuring ethical approaches, risk management, and community empowerment.
2. **Collaborative and Inclusive Research Leadership** fostering diversity, inclusion, and synthesis of diverse perspectives.
3. **Interdisciplinary Networking for Research Innovation** enabling cross-sector networking, innovation, and eco-social impact.
4. **Methodological Breadth with Societal Impact** promoting open, equitable research and evaluation of impact for a sustainable future.
5. **Resilient Capacity Development** supporting conflict resolution, adaptability, and resource management for sustained contributions in dynamic contexts.

This dual structure reflects the complexity of research competence—simultaneously requiring systematic coverage of discrete skills (8Cs) and integrated development of capability clusters (5 factors). For practitioners, the 8Cs provide scaffolding for comprehensive development, while the factors indicate priority areas for targeted intervention.

### Key empirical insights

Qualitative findings reveal that students actively engage with complex ethical issues, including responsible use of artificial intelligence, reflecting critical awareness of emerging technologies' societal impacts. However, persistent institutional barriers to equity, diversity, and inclusion remain ( Owens & Brant, 2023). Senior researchers highlight systemic challenges—funding inequities, organisational rigidity, and academic surveillance—that constrain ethical and impactful research enactment.

### Policy and framework alignment

The framework's strong emphasis on interdisciplinary and impactful research directly addresses the limitations of siloed disciplinary training. Horizon Europe's Strategic Plan 2025–2027 ( European Commission, 2023) calls for such approaches to advance societally engaged research. Aligned with these aims, upSkill.Map develops the capacity to work across fields and to tackle societal and environmental challenges holistically, in ways consistent with Responsible Research and Innovation (RRI) principles ( Nature, 2024; Owen *et al.*, 2022). This design encourages research that is not only innovative but also ethically grounded with and for society.

Embedding collaborative leadership, inclusive communication, and resilience equips researchers with interpersonal and adaptive capacities to work with diverse stakeholders, manage uncertainty, and sustain contributions in challenging environments. Such capacities are increasingly recognised as critical to fostering research cultures that value inclusivity, ethical rigor, and sustainability (RMComp, EC, 2025).

At the policy level, upSkill.Map aligns with global agendas such as the United Nations Sustainable Development Goals—particularly SDG 4 (Quality Education), SDG 10 (Reduced Inequalities), and SDG 16 (Peace, Justice, and Strong Institutions)—and the UN Global Sustainable Development Report (2023), which underscores integrating SDGs into education and researcher professionalisation. Regionally, it supports the EU Horizon Europe pillars of RRI, interdisciplinarity, and societal engagement. Nationally, it complements the UK's Vitae Researcher Development Framework ( Vitae, 2025) by explicitly foregrounding ethics, justice, and sustainability as core, not optional. In doing so, it responds to critiques of earlier RDF versions and supports evolving conceptions of researcher identity and professionalisation in UK higher education ( Mcgloin & Wynne, 2022; Taylor & Archer, 2022).

### Multi-level utility

The upSkill.Map instrument operates at individual, institutional, and sectoral levels. It supports personal reflection and career navigation (micro), informs programme design and policy refinement (meso), and aligns with national and global frameworks (macro). This multi-layered utility ensures findings extend beyond our sample, offering a transferable model for assessing researcher development needs and designing equitable, impactful programmes. The UpSkill.Map instrument can also be applied in programmes aimed at addressing research training and job market gaps in contexts where most academics hold MSc degrees below the internationally recognised PhD/DEng/DTech level, thereby contributing to global education initiatives that enhance mobility, knowledge exchange, and professional career development ( Nyemba & Carter, 2020).

### Institutional implementation context

This multi-institutional collaboration demonstrates the instrument's practical application across diverse academic contexts. Within the Faculty of Science, Technology, Engineering, and Mathematics (STEM), the instrument aligns with researcher career development priorities, including initiatives such as early career researcher hubs that consolidate training programmes ( Mostéfaoui & Gardner, 2021) and opportunities for engagement with experienced academics. Its integration can enhance programme innovation (Open University, 2020) and equip learners with transferable skills relevant to corporate, educational, and international contexts. Within the Faculty of Wellbeing, Education, and Language Studies (WELS), the instrument supports strategic goals of fostering inclusive and empowering cultures, meaningful development pathways, and systems for continuous improvement. By embedding upSkill.Map across programmes and development initiatives ( Open University, 2018; Open University, 2024a), it promotes values-led practice and strengthens capacity to adapt to evolving educational landscapes, supporting both academic and professional services staff in advancing equality, diversity, and inclusion initiatives.

### Limitations and future directions

This foundational validation study establishes the psychometric properties and theoretical coherence necessary for larger-scale implementation. Future research should prioritize multi-institutional validation across diverse higher education contexts, including European partnerships aligned with Horizon Europe priorities for responsible research and innovation. Such expansion would enable assessment of the instrument's capacity to drive systemic change in researcher development practices, moving beyond individual reflection to institutional transformation. The current findings provide the empirical foundation for testing whether this paradigmatic shift from supplementary to core ethics integration can achieve sectoral impact.

**Recommendations**

1. Embed resilience and capacity-building training early in research careers
2. Reform promotion and reward systems to recognise inclusive leadership and collaborative contributions
3. Increase institutional support for equitable access to networks and intersectoral collaboration
4. Create incentives and structural enablers for interdisciplinary work, aligned with Horizon Europe and RRI principles
5. Integrate sustainability, equity, and justice into national researcher development frameworks as non-negotiable core competencies

By embedding justice, equity, and sustainability as non-negotiable elements, upSkill.Map reframes researcher development for the 21st century, with potential to drive both individual capability and systemic transformation.

## Conclusion

The upSkill.Map instrument, grounded in the CARE–KNOW–DO ( Okada, 2025b) framework, offers an original, evidence-based approach to doctoral and early-career researcher development, aligned with EU ( ESCO, 2025; European Union, 2025) and UNESCO ( UNESCO, 2018; United Nations, 2023) priorities integrating skills development for post-Agenda 2030. By integrating the 8C framework, the instrument connects transversal research skills with discrete competences—such as analysis of priorities, research writing, and grant applications—and situates them within real-world contexts, societal needs, and evolving professional identities.

Through a design-based research (DBR) methodology that combined psychometric validation, thematic analysis, expert review, and user feedback, the study demonstrated methodological rigour and resulted in a reliable, coherent, and practically usable instrument. While this single-institution study establishes foundational validity, the instrument's potential for systemic impact requires demonstration across diverse institutional contexts. The theoretical contribution—positioning sustainability ( Piccardo *et al.*, 2022), equity ( Chankseliani, 2023), and justice ( Cole *et al.*, 2023) as foundational rather than supplementary—provides a framework for larger-scale studies that can assess whether this paradigmatic shift drives meaningful change in researcher development practices across the sector.

Findings recommend strengthening professional networking, leadership, resilience, interdisciplinary collaboration, and research translation ( Passmore *et al.*, 2020) for societal benefit. Echoing Martínez & Tejero (2025), systemic action in standards, recognition, certification, networks, and outreach is required to reinforce the professional status of research managers and support sustainable research ecosystems.

This study also highlights the potential of upSkill.Map to support researchers in self-assessing and redesigning their professional development, guided by both institutional and external opportunities and further informed by its findings, supporting both personal fulfilment and global relevance. Aligned with Perkins’ (2010) principle of “making learning whole,” UpSkill.Map illustrates how this structured, evidence-based instrument in real-world challenge settings can enrich research education and professional development. By supporting mega-knowledge production to advance research-based solutions to complex global challenges (Powell, 2024), UpSkill.Map fosters competencies that are technically sound, ethically grounded, epistemically plural, and socially responsive, contributing to more equitable and sustainable futures in higher education and beyond, for the global commons.

## Ethics declarations

The study was conducted in accordance with the Declaration of Helsinki and approved by The Open University Human Research Ethics Committee (HREC 2025-0889-4).

## Informed consent statement

Informed consent was obtained from all participants involved in the study (written form).

## AI use disclosure

This study made selective use of AI technologies to support the research and writing process. Perplexity was used to assist with APA format revisions; ChatGPT supported abstract reduction (300 words); an AI mapping tool generated a UML image; Springer AJE ProofReader provided language editing; and ATLAS AI assisted with thematic analysis. Quantitative analyses were conducted with SPSS, R, and Claude. All outputs were carefully reviewed, verified, and validated by two researchers to ensure accuracy and integrity.

## Data Availability

Data are available via Open Research Data Online (ORDO) of The Open University. The dataset was aligned with the project’s data management plan and reviewed by the Open Research Library Team. This database includes the views of 40 researchers who participated in the upSkill study: Dataset upSkill.Map [Data set]. The Open University. https://doi.org/10.21954/ou.rd.30108928.v2 Okada (2025a) The questionnaire is part of the UK METEOR studies and the CONNECT project: upSkill.Map self-reported instrument [Questionnaire]. The Open University. https://doi.org/10.21954/ou.rd.30109057.v1 Okada (2025a) Data are available under licence (CC BY-SA)
