# Developing Researchers’ Competencies through CARE–KNOW–DO and upSKILL.map, aligned with EU and UNESCO Priorities

**DOI:** 10.12688/openreseurope.21408.3

**Published:** 2026-05-29

**Authors:** Alexandra Okada, Kieron Sheehy, Klaus-Dieter Rossade, Arosha K. Bandara

**Affiliations:** 1 Global Education and Digital Transformation Network, Faculty of Wellbeing, Education and Language Studies (WELS), The Open University, Milton Keynes, MK 76AA, UK; 2 Rumpus Research Group, Faculty of Wellbeing, Education and Language Studies (WELS), The Open University, Milton Keynes, MK7 6AA, UK; 3 Faculty of Wellbeing, Education and Language Studies (WELS), The Open University, Milton Keynes, MK7 6AA, UK; 4 Faculty of Science, Technology, Engineering and Mathematics (STEM), The Open University, Milton Keynes, England, UK

**Keywords:** Researchers’ Competencies, Doctoral Education, CARE-KNOW-DO framework, EU Agenda, UNESCO SDGs

## Abstract

**Background:**

In an era of interlinked global challenges, researchers are expected to combine disciplinary excellence with socially relevant solutions. Existing competency frameworks recognise transversal skills but tend to prioritise employability and career management, treating skills as value-neutral and positioning sustainability, equity, and justice at the margins. This creates skills mismatches and leaves many researchers underprepared for work aligned with the UN-SDGs and Horizon Europe priorities.

**Methods:**

This Phase-1 study within the European METEOR project develops upSKILL.map and provides in-context pilot validation for eco-outwards research careers. Grounded in the CARE–KNOW–DO principles, the tool articulates eight competency domains (8Cs) integrating values, knowledge, and action. A phase-appropriate cohort of 40 researchers completed the 8Cs self-assessment and wrote reflective think pieces, generating a mixed-methods dataset for contextual psychometric analysis and thematic enquiry.

**Results:**

Exploratory factor analysis, reliability testing, expert review, and user feedback converge on a five-factor descriptive clustering explaining 75.7% of variance as a baseline, clustering the 8Cs into: Responsible policy-engaged research; Collaborative inclusive leadership; Interdisciplinary networked innovation; Societal impact methodologies; and Resilient capacity development. Datasets highlight an aspiration–practice gap between collaborative, transformative goals and uneven institutional support.

**Conclusion:**

Conceptually, the study advances a shift from “competency-as-performance” to “competency-as-worldview”, articulating the eco-researcher identity where technical expertise is inseparable from commitments to sustainability, equity, and justice. This represents one valued orientation among legitimate research identities; fundamental science and curiosity-driven inquiry remain equally valid. Methodologically, it provides context-bound evidence that upSKILL.map can function as a low-cost diagnostic tool combining psychometric analysis with qualitative insight. Organisationally, it outlines how doctoral schools and researcher development leads can use the five-factor clustering to identify gaps and design targeted interventions for local and global agendas. Exploratory findings are based on a single institution and a small, self-selected cohort, so further multi-institutional confirmation is needed before high-stakes or sector-wide use.

## 1. Introduction

### 1.1 The imperative for a new research paradigm

**Figure 1.**
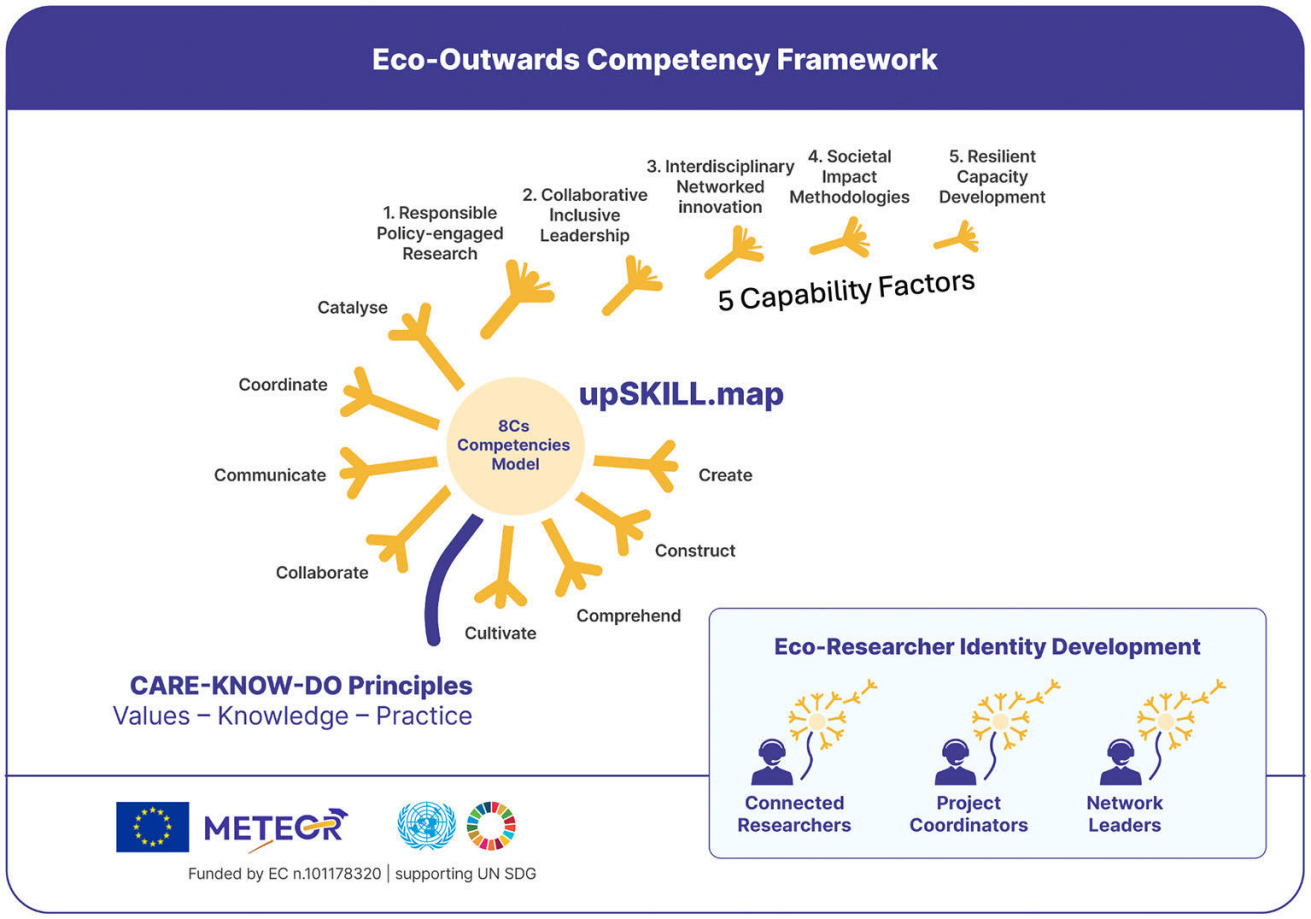
Eco-outwards competency framework operationalised by upSKILL.map: from the 8Cs competency domains to five capability factors across three researcher profiles, grounded in CARE–KNOW–DO principles. Source: Okada (2025a).

We live in an era defined by the growth of global polycrises: climate breakdown, intensified inequalities, and technological disruption ( Lawrence *et al*., 2024; Chankseliani, 2023). The researcher’s role is changing in many fields. For many research careers, producing disciplinary excellence in isolation is no longer sufficient ( Yudkevich *et al*., 2020; Baker & Powell, 2024). Researchers increasingly need transversal competences—skills with knowledge and values applicable across sectors and occupations—to drive socially relevant, sustainable, and transformative solutions ( UNESCO, 2017; United Nations, 2023; Martínez & Tejero, 2025; Okada 2025b). Figure 1 presents the Eco-Outwards Competency Framework operationalised by upSKILL.map, which addresses these gaps through CARE-KNOW-DO principles underpinning 8Cs competencies clustered into five capability factors.

According to the European Framework for Skills, Competences, Qualifications and Occupations ( ESCO, 2025), transversal competences function as foundational infrastructure: *“Transversal knowledge, skills and competences are relevant to a broad range of occupations and economic sectors. They are often referred to as basic skills, foundational competences or soft skills, the cornerstone for the personal development of a person. Transversal knowledge, skills and competences are the building blocks for developing the ‘hard’ skills and competences required to succeed on the labour market” ( ESCO, 2025).*

Yet this definition exposes a critical gap: it treats transversal competences as isolated skills, rather than as a capability learners apply purposefully in real-world contexts. A regenerative worldview integrating technical mastery, ethical commitment, and temporal responsibility to future generations is absent from current frameworks. Although the European Commission’s GreenComp framework ( Bianchi *et al*., 2022) advances sustainability competence ( Michel *et al*., 2023) by emphasizing systems thinking and values reflection, and ESCO’s recent Green Skills initiative (2025) labels transversal competences ( Taylor & Clark, 2023; Roy *et al*., 2025) for environmental behaviour, we argue that both frameworks share a fundamental limitation. Neither framework positions sustainability and equity as fundamental to researcher worldview, nor do they articulate researchers’ ethical commitments to future generations, with emphasis on futures literacy. The researcher remains positioned as a qualified professional within present-day labour markets, not as a competent transformative agent with ethical commitments across generations. This requires recognition of an essential distinction: intergenerational responsibility is ethical, not transactional.

### 1.2 Existing frameworks

Major competence architectures for researchers ( Table 1), including ResearchComp ( European Commission, 2025a), the Researcher Development Framework (RDF) ( Vitae, 2025), and the OECD Core Competency Framework ( OECD, 2019), have advanced recognition of transferable skills and professional development across research careers. These frameworks define structured domains of competence intended to support employability, career progression, and research effectiveness ( Lee *et al*., 2022; Garcia *et al*., 2017). Yet, they largely prioritise career management and performance, often treating skills as value-neutral capabilities that can be applied independently of ethical, ecological, or social commitments ( Mulder, 2014; Patel *et al*., 2020). Crucially, sustainability and equity are not positioned as foundational, non-negotiable dimensions of research practice ( Wiek *et al*., 2011).

**Table 1.** Comparative analysis of researcher competency frameworks.

Feature	ResearchComp (EU)	Vitae RDF (UK)	OECD Core Framework	upSKILL.map: Eco-researcher
Primary Focus	Employability & Career	Development and Leadership	Skills for Labor Market	Sustainable & Responsible Research
Sustainability	Peripheral (Self-org.)	Implicit/Peripheral	Absent	Foundational (Central Pillar)
Equity & Justice	Absent	Peripheral	Absent	Foundational (Central Pillar)
Theoretical Model	Competency-as-Performance	Competency-as-Performance	Human Capital	Competency-as-Worldview
Key Dimensions	Cognitive & Interpersonal	4 Domains	Cognitive & Socio-Emotional	CARE–KNOW–DO principles
Target Outcome	Competitive Workforce	Professional Excellence	Economic Productivity	Planetary & Public Good

In both the Vitae RDF and EU ResearchComp, references to sustainability focus primarily on financial viability, infrastructure continuity, and individual work–life balance rather than ecological integrity or social justice ( European Commission, 2025a, p. 27; Vitae, 2025, p. 15). The RDF refers to “strengthening organisational funding sustainability” and contributing to the financial sustainability of projects and programmes, equating sustainability with institutional continuity. Similarly, ResearchComp emphasises sustainable work practices, funding models, and resource management. This framing reflects an efficiency-oriented interpretation in which sustainability serves to maintain research productivity and institutional stability, rather than to guide ethical responsibility toward planetary and societal wellbeing.

The Vitae RDF, ResearchComp and the OECD Core Framework address purposes distinct from those of the eco-outwards framework — respectively, researcher career development and leadership, employability across European research labour markets, and cross-occupation skills comparison. The eco-outwards framework articulates one legitimate professional orientation and worldview among many. upSKILL.map is designed for researchers whose work engages with societal, policy, or sustainability challenges — a constituency currently underserved by existing frameworks — and is not a universal model for all research careers. Disciplinary excellence, curiosity-driven inquiry, and fundamental science remain equally valid research identities, each shaped by different developmental priorities that fall outside the scope of this paper.

Building on CARE–KNOW–DO ( Okada & Gray, 2023; Okada, 2025b), this paper introduces the eco-outwards research framework that underpins upSKILL.map. The framework integrates values (CARE), knowledge (KNOW), and action (DO) across eight competency domains, positioning sustainability not as an operational requirement but as an ethical and epistemic commitment to planetary stewardship, intergenerational responsibility, and social justice. While sustainability scholarship distinguishes between short-term resilience and long-term ecological stewardship ( Summers *et al*., 2017; Wilson & Wu, 2017), this temporal–ethical dimension is largely absent from dominant competence models — a gap upSKILL.map is designed to address.

This reframes researcher competence as the ethical stewardship of values, knowledge, and practice across time, shifting from a performance-based construct toward a worldview-oriented orientation in which sustainability, equity, and systems awareness function as organising principles rather than peripheral skills. Employability is not displaced but repositioned: the eco-researcher integrates technical expertise with social justice and systems-level responsibility ( Boni & Gasper, 2012; Cole *et al*., 2023; Owen *et al*., 2022), aligning researcher development with the UN Sustainable Development Goals and Horizon Europe’s responsible research and innovation agenda.

Building on these foundations, this paper introduces an empirically derived five-factor structure and diagnostic instrument (upSKILL.map), enabling individual- and programme-level identification of aspiration–practice gaps related to sustainability, equity, and regenerative futures. In doing so, it proposes eco-social responsibility — understood here as the integration of environmental sustainability and social equity — as a foundational organising principle for researcher development rather than an optional extension of existing competency models.

### 1.3 Research approach, aims, and research questions

Realising this reframing empirically and at scale requires understanding how institutional conditions enable or constrain eco-outwards competency development. This Phase-1 study focuses strategically on doctoral and early-career researchers at a single institution — The Open University, UK — as a situated, in-depth case.

This design prioritises contextual depth over breadth. Doctoral and early-career researchers represent critical leverage points where professional identity is most malleable, making them ideal subjects for studying how institutional conditions shape emerging research culture. A single-institution focus enables detailed documentation of barriers and enablers that multi-institutional surveys cannot capture.

The eco-outwards competency framework ( Table 2, Figure 1) rests on four interrelated constructs: principles, worldview, competencies, and a diagnostic instrument. **CARE–KNOW–DO** provides the epistemological foundation, integrating values (CARE), knowledge (KNOW), and practice (DO) as inseparable dimensions of researcher competence. This worldview shapes the **eco-researcher** — a professional orientation extending beyond disciplinary and institutional boundaries toward planetary stewardship and socio-environmental justice.

**Table 2.** Eco-Outwards Research Framework: *components, types, and functions.* Source: Okada (2025b).

Component	Type	Function
Eco-outwards research framework	Theoretical framework — conceptual reference	Supports researcher competence as eco-social stewardship shaping eco-researcher identity
CARE–KNOW–DO principles	Foundation — epistemological basis	Integrates values, knowledge, and practice as inseparable dimensions of researcher competence
Eco-researcher identity	Professional orientation construct — profile	Consolidates researcher orientation toward planetary stewardship and socio-environmental responsibility
8Cs competencies model	Competence model — practical reference	Defines eight core competencies-as-worldview required for eco-researcher development
Five capability factors	Capabilities — capability structure	Enables eco-researcher development across five dimensions, empirically derived higher-order clusters of the 8Cs
upSKILL.map	Diagnostic instrument — assessment tool	Operationalises the 8Cs and five factors to assess aspiration–practice gaps and support eco-researcher development

The **8Cs model** operationalises the eco-researcher identity into eight practice domains (Collaborate, Communicate, Cultivate, Construct, Comprehend, Create, Coordinate, Catalyse). As the findings in §4 show, these eight domains cluster empirically into **five capability factors**: Responsible policy-engaged research; Collaborative inclusive leadership; Interdisciplinary networked innovation; Societal impact methodologies; and Resilient capacity development. **upSKILL.map** assesses researchers’ current position across the 8Cs, surfaces aspiration–practice gaps, and supports targeted development at individual and programme level.

This paper reports a Phase-1 contextual pilot validation of the upSKILL.map instrument within the European Commission (EC)–funded METEOR project (Methodologies for Teamworking in Eco-outwards Research), using a design-based research approach. The study aims to: (1) examine the structural coherence of the 8Cs competency model; (2) identify latent competency factors prioritised by researchers and associated aspiration–practice gaps; and (3) generate diagnostic evidence on institutional conditions that support or constrain eco-outwards competencies.

This Phase-1 study is designed to generate, in preparation for Phases 2 and 3: (1) actionable institutional insights; (2) a foundation for multi-institutional networked research across institutions, sectors, and nations. Two mechanisms justify this approach: **career-stage leverage** (doctoral training embeds eco-outwards competencies at a formative stage; and **methodological replicability** (careful psychometric analysis at realistic scale, provides a model that under-resourced institutions can adapt without requiring equivalent infrastructure).

The paper addresses two research questions:

**RQ1** *(conceptual/structural)*: How coherent and robust is the 8Cs competency model as an expression of the eco-researcher identity?

**RQ2** *(empirical/priorities)*: What latent competency factors emerge from researchers’ responses, and which eco-outwards competencies and institutional barriers do they prioritise?

Rather than testing specific interventions, this Phase-1 study generates diagnostic insights institutions can use to embed sustainability, equity, and social responsibility into doctoral training and researcher development programmes. §2 details the theoretical foundations of this framework; §3 describes how it was developed and tested; §4 and 5 report and interpret the findings.

## 2. Development of the upSKILL.map instrument

### 2.1 Theoretical foundations

The upSKILL.map instrument demonstrates the CARE–KNOW–DO principles by integrating six complementary theoretical principles from diverse theories that together provide a robust and values-led foundation for doctoral education and researcher professional development ( Table 3). These principles are distinct but mutually reinforcing, addressing metacognition, competence, transformation, responsibility, sustainability, and equity.

**Table 3.** Theoretical Frameworks and their Contribution to *upSKILL.map.*

Theoretical References	Core Concepts	How It Underpins *upSKILL.map*
1. Self-Assessment and Self-Regulated Learning	Self-efficacy, goal setting, metacognition	Scaffolds reflective, self-directed growth through prioritisation and rating tools
2. Competency-Based and Developmental Models	Holistic capability, researcher identity, progression	Structures the 8Cs and CARE–KNOW–DO model as developmental pathways
3. Transformative Learning	Critical reflection, perspective shift, worldview	Supports values clarification and societal role development
4. Responsible Research and Innovation (RRI)	Inclusion, reflexivity, public accountability	Anchors Catalyse and CARE domains in anticipatory, and ethical engagement
5. Sustainability Science and Regenerative Education	Systems thinking, action competence, futures literacy, regenerative principles	Aligns development with SDGs and future-focused interdisciplinary research, and positions researchers as co-creators of sustainable futures
6. Equity-Oriented Development	Capabilities, justice, wellbeing	Promotes inclusion, supports researchers from and working within marginalised regions, and challenges deficit models

Table 3’s six references map to CARE–KNOW–DO principles: CARE anchors emotional/values-based engagement (self-assessment, transformative learning, equity); KNOW builds evidence/systems competency (RRI, sustainability science); DO drives implementation (action/equity). This structures the 8Cs domains, which Exploratory Factor Analysis (EFA) condenses into five emergent factors ( §4).

***2.1.1 Self-Assessment and self-regulated learning***

Self-Assessment Theory ( Hattie & Timperley, 2007) and Self-Regulated Learning ( Zimmerman, 2002; Zimmerman & Schunk, 2011) position learners as active agents in their development. upSKILL.map builds on these by enabling researchers to evaluate their skills, prioritise areas for growth, and reflect on progress over time. This scaffolds self-efficacy, feedback use, and goal-directed monitoring, turning reflection into actionable strategies for professional growth.

***2.1.2 Competency-based and developmental models***

Competency-Based Education ( Eraut, 1994; Mulder, 2014) views competence as the integration of knowledge, skills, values, and attitudes in real contexts. Developmental frameworks such as Vitae RDF, and EC ResearchComp highlight progression from novice to expert. The upSKILL.map instrument adopts and values these approaches but extends them beyond short-term employability or immediate task performance. It combines staged ratings with the CARE–KNOW–DO principles to support a holistic and authentic approach that integrates motivation, understanding, and practice:

• CARE: values and engagement (Communicate, Collaborate, Cultivate)
• KNOW: knowledge and analysis (Comprehend, Construct, Coordinate)
• DO: application and impact (Create, Catalyse).

By connecting competence with purpose, expertise, and practice, upSKILL.map reconceives researcher development as more than skill acquisition, emphasizing the cultivation of worldview capabilities and sustainable-futures identities within authentic, holistic contexts ( Gardner, 2011; Perkins, 2010).

***2.1.3 Transformative learning***

Transformative Learning Theory ( Chen *et al.*, 2020; Cranton, 2006; Mezirow, 1997) emphasises reflection and perspective change. upSKILL.map incorporates this by embedding indicators of justice, equity, and sustainability, encouraging researchers to question assumptions and align professional identities with wider societal commitments.

***2.1.4 Responsible Research and Innovation (RRI)***

RRI ( Stilgoe *et al.*, 2013; von Schomberg, 2013) reframes research as anticipatory, reflexive, inclusive, and responsive. upSKILL.map integrates these dimensions by encouraging open science, stakeholder engagement, and ethically informed decision-making. RRI thus provides both an ethical compass and a practical framework for aligning innovation with societal needs.

***2.1.5 Sustainability science***

Sustainability Science ( Langston, 2021; UNESCO, 2017; Wiek *et al.*, 2011) identifies competencies such as systems thinking, action competence, and future literacy. Futures literacy ( UNESCO, 2020) enables researchers to anticipate, imagine, and prepare for multiple possible futures, moving beyond prediction to cultivate adaptive capacity in uncertainty. Regenerative principles ( Sterling, 2021; van den Berg & Wals, 2022) shift the paradigm from “doing less harm” to actively restoring and revitalizing socio-ecological systems. Together, these inform the instrument’s emphasis on interdisciplinarity, scenario planning, and readiness to act for transformative change, equipping researchers to address complex, interconnected global challenges and co-create sustainable, just futures ( Piccardo *et al*., 2022).

***2.1.6 Equity-oriented models of development***

Equity-focused perspectives ( McAlpine & Amundsen, 2018; Raffaghelli *et al.*, 2020; Walker & Boni, 2006) emphasise epistemic justice, wellbeing, and the inclusion of diverse voices. upSKILL.map enacts these by providing activities that surface diverse knowledge systems valuing plural knowledge systems, (2) supporting underrepresented groups in mapping their competencies and aspirations, and (3) fostering collaborative reflection through transdisciplinary “thinking pieces,” allowing participants to co-construct knowledge, and challenge deficit models of researcher development.

Together, these six principles link individual agency to collective responsibility, connecting technical capability with ethical purpose, sustainability and equity ( Abdelghaffar & Eid, 2025) in researcher development. They foreground participatory science with and for society, supporting iterative listening, feedback and co-design across the research process, and position upSKILL.map to help eco-outwards researchers address complex, interconnected global challenges ( Langston, 2021; UNESCO, 2017; Wiek *et al*., 2011).

### 2.2 Instrument constructs

upSKILL.map translates these theoretical references into a practical developmental self-assessment instrument. It is organised around the 8Cs model ( Table 4), aligned with the CARE–KNOW–DO principles as described in §2.1.2. The model specifies eight domains of capability, each defined by four indicators (32 in total). This design produces both individual profiles and collective patterns of researchers’ capability.

**Table 4.** Mapping the 8Cs to Theoretical Concepts ( *Superscript numbers refer to Sections 2.1.1 to 2.1.6*).

8C Domain	Core Competency Constructs	Key Concept
**Collaborate** *Support interdisciplinary research* *partnerships*	Interdisciplinary teamwork, Team contribution, Conflict resolution, Inclusive diversity	Metacognition ^1^, Interdisciplinarity ^2^, Inclusion ^6^, Agency ^6^
**Communicate** *Share accessible and intercultural* *knowledge*	Science communication, Network building, Policy engagement, Intercultural dialogue	Feedback use ^1^, Reflexivity ^4^, Perspective shift ^3^
**Cultivate** *Nurture lasting, inclusive professional growth*	Mentoring support, Community participation, Event organisation, Reflective Practice	Self-efficacy ^1^, Capabilities approach ^6^, Wellbeing ^6^
**Construct** Build evidence-based, participatory solution	Research design, Literature synthesis, Methodological innovation, Ethical practice	Knowledge integration ^2^, Systems thinking ^5^, Ethics ^5^
**Comprehend** *Critically understand diverse perspectives*	Systems analysis, Evidence evaluation, Information synthesis, Creative problem-solving	Systems thinking ^5^, Critical reflection ^3^, Cognitive regulation ^1^
**Coordinate** *Lead inclusive, engaging, and committed teams*	Resource management, Priority balancing, Risk management, Team leadership	Developmental progression ^2^, Leadership ^2^, Anticipation ^4^
**Create** *Develop responsible research and* *innovation*	Interdisciplinary links, Innovative thinking, Knowledge cocreation, Adaptive research	Creativity ^3^, Boundary-crossing ^5^, Innovation competence ^2^
**Catalyse** Drive sustainable change for the common good	Open research, Policy innovation, Community empowerment, Impact evaluation	Public value ^4^, Ethics ^5^, Societal responsibility ^6^

This mapping illustrates how the instrument’s design is not arbitrary but systematically integrates metacognition, progression, transformation, responsibility, sustainability, and equity. Each 8C is grounded in one or more of the six principles; together, the 8Cs express how eco-researchers ‘care’ about societal challenges, ‘know’ systems and evidence, and ‘do’ transformative work.

### 2.3 Instrument overview

The upSKILL.map is a diagnostic and developmental instrument that operationalises the 8Cs competencies model and five capability factors to assess eco-researcher capability and identity formation. Grounded in the CARE–KNOW–DO principles, it integrates quantitative self-assessment with structured qualitative reflection to examine capability development, professional priorities, and alignment between researcher aspirations and enacted practice.

**Figure 2.**
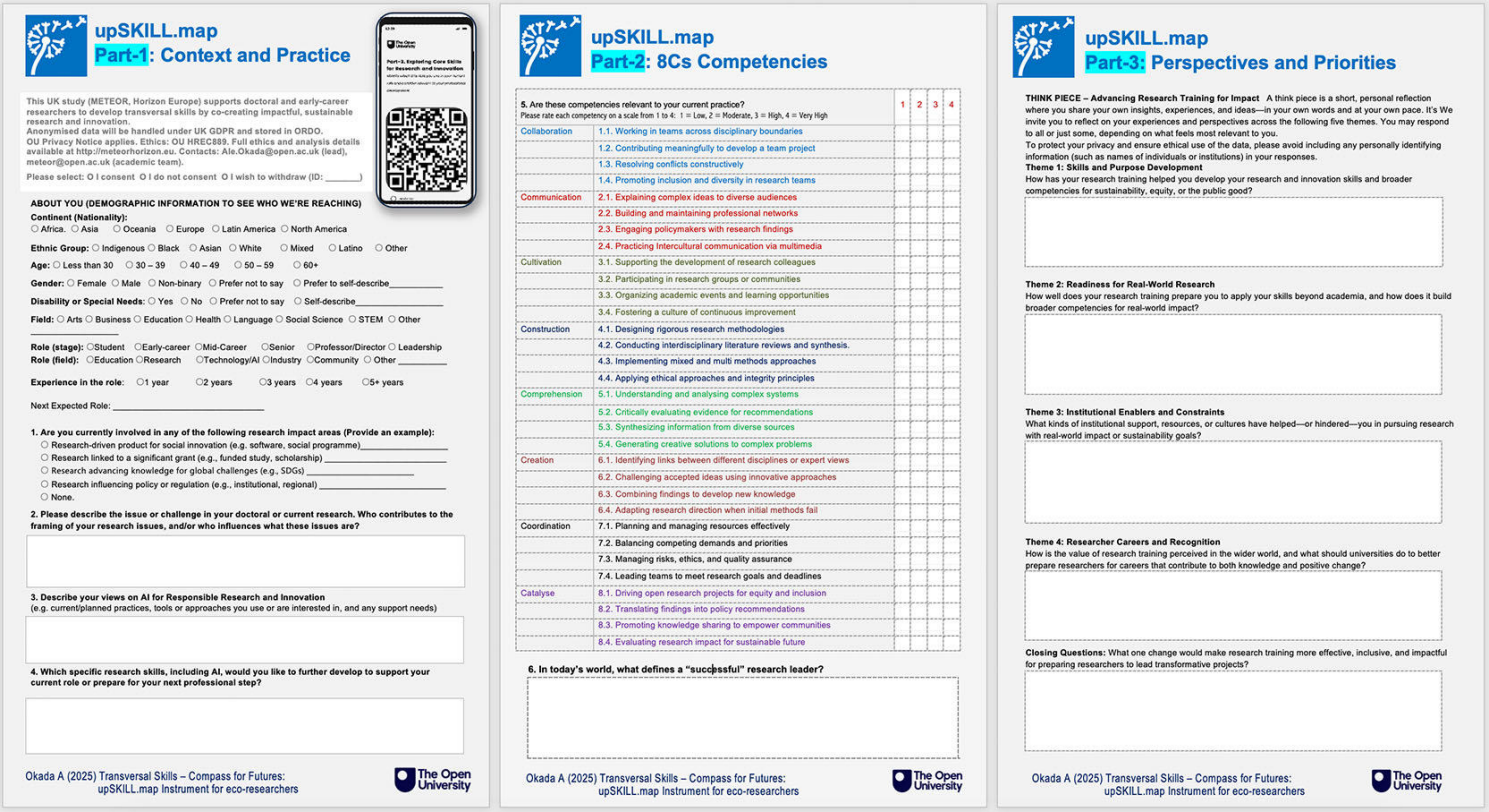
The upSKILL.map self-assessment instrument (Qualtrics interface), showing Part-1: Context and Practice, Part-2: 8Cs Competencies, and Part-3: Perspectives and Priorities. Accessible via QR code on mobile, tablet, or computer. Source: Okada, 2025a.

As shown in Figure 2, the instrument is organised into three sequential parts, intentionally scaffolded so that each part builds on the previous: Part-1 establishes the contextual and experiential foundation; Part-2 maps capability against that foundation; and Part-3 invites researchers to interpret, reflect on, and integrate both into a coherent professional orientation narrative.

***Part-1: Context and practice***

Part-1 grounds the instrument in each participant’s lived research context before any capability rating takes place. It opens with demographic data (continent, ethnicity, gender, disability, field, role stage, and experience) to support analysis of diversity, inclusion, and institutional representativeness. Participants then identify their current involvement in research impact areas — including social innovation products, major grant-funded initiatives, contributions to global challenges such as the SDGs, and policy or regulatory influence — establishing the professional terrain within which their capabilities will be assessed.

Two open questions deepen this contextualisation: participants describe the issue or challenge in their doctoral or current research and reflect on who contributes to the framing of research priorities — embedding reflexivity on power and agenda-setting from the outset. A further question invites participants to describe their views on AI for Responsible Research and Innovation, including current or planned practices, tools, and support needs. This is intentionally positioned as a contextual and cross-cutting question rather than a discrete competency item: AI literacy is conceived throughout the instrument as an enabling capability that can amplify all eight competency domains, not as a standalone competency reducible to a single indicator among the 32. A closing question asks which research skills, including AI, participants wish to develop further — connecting Part-1 directly to the capability assessment that follows.

***Part-2: 8Cs competency model***

Building on the contextual foundation established in Part-1, Part-2 presents a structured quantitative self-assessment of 32 competency indicators derived from the 8Cs model, organised across the eight competency domains (Collaborate, Communicate, Cultivate, Construct, Comprehend, Create, Coordinate, Catalyse). Participants rate each indicator on a four-point developmental scale (1 = vlow, 2 = moderate, 3 = high, 4 = very high) according to its relevance to their current practice. It is important to note that Part-2 ratings reflect participants’ perceived relevance of each indicator to their current practice, rather than an objective assessment of demonstrated competence. A closing open question — “In today’s world, what defines a successful research leader?” — bridges the structured rating into the reflective inquiry of Part-3, inviting participants to articulate their own normative vision of research excellence before reflecting on their own development.

***Part-3: Perspectives and priorities***

Part-3 consists of a qualitative think piece structured around five thematic prompts that progressively move from individual capability through institutional context to professional orientation: (1) Skills and Purpose Development — how training has shaped skills, values, and alignment with sustainability and the public good; (2) Readiness for Real-World Research — how well prepared researchers feel to apply their skills beyond academia to address societal challenges; (3) Institutional Enablers and Constraints — what kinds of support, resources, or cultures have helped or hindered research with real-world impact; (4) Researcher Careers and Recognition — how research training is perceived in the wider world and what universities should do to better prepare researchers for careers contributing to knowledge and positive change; and a Closing Question asking what one change would make research training more effective, inclusive, and impactful for transformative projects. Together these prompts function as a narrative inquiry space ( Clandinin & Connelly, 2000), capturing how capability is interpreted and enacted across career stages and institutional contexts.

The instrument is designed for completion in approximately 20–30 minutes and is administered via Qualtrics, allowing asynchronous participation on mobile devices, tablets, or computers. By combining contextual mapping, structured capability assessment, and reflective inquiry, upSKILL.map generates a coherent, multi-layered evidence base for analysing researcher capability, aspiration–practice alignment, and eco-researcher development across disciplines and institutional contexts.

### 2.4 Embedding power and agenda-setting reflexivity

Critically, upSKILL.map does not present societal challenges as pre-defined problems for researchers to “solve.” Instead, participants reflect on how research issues and priorities are developed—for example, through engagement with partners, funders, communities, local policy contexts, or global agendas.

Participants are prompted to consider the issues or challenges in their doctoral or current research, focusing on who shapes the framing of research questions and who influences what these issues become. They can illustrate this with examples such as:

• Research-driven products for social innovation (e.g., patents, software, social programmes);
• Research linked to significant grants (e.g., funded studies, scholarships);
• Research advancing knowledge for global challenges (e.g., Agenda 2030);
• Research influencing policy or regulation (e.g., institutional or regional);
• or none of the above.

This design embeds reflexivity on power and agenda-setting by describing their own research and its co-definition processes, participants develop critical awareness of how priorities are negotiated with community, policy, and practitioner stakeholders.

By integrating experiential accounts, structured self-assessment, and reflective narratives, upSKILL.map operationalizes core competencies for eco-outwards research, emphasizing researcher agency, participatory engagement, epistemic justice, equity, and shared agenda-setting.

## 3. Methodology

This study employed a multimethod approach, combining design-based research (DBR; The Design-Based Research Collective, 2003; Anderson & Shattuck, 2012) to iteratively develop the upSKILL.map self-assessment instrument, with an explanatory sequential mixed-methods design in which quantitative findings informed subsequent qualitative inquiry) for in-context pilot validation. This study constitutes Phase-1 of a multi-phase DBR programme; findings are therefore treated as exploratory and provisional, subject to confirmation through Phase-2 replication and instrument refinement.

### 3.1 Research design: Phase-1 study in a multi-phase programme

This three-phase programme positions Phase-1 findings within a larger transformation agenda: - Phase-1 (reported here): Establishes institutional model and generates insights about barriers and enablers at the Open University. - Phase-2 (currently underway): Tests whether Phase-1 insights replicate and adapt across two external METEOR academies (48+ participants, 1-year institutional integration). Methodological details in §3.2. - Phase-3 (planned): Multi-institutional comparative study tracking how research cultures transform toward eco-outwards paradigms, enabling regions and nations to track progress, identify bottlenecks, and learn from comparative implementation experiences. Detailed research questions and sample specifications in §5.5.

DBR ( Design-Based Research Collective, 2003) was chosen for its capacity to integrate theory and practice in authentic educational contexts, allowing continuous refinement through empirical feedback and stakeholder engagement. Participants received full project information and provided written informed consent prior to engaging with the upSKILL.map instrument activities, with consent obtained through a differentiated framework allowing individuals to specify permissions. Information about the project and written informed consent were embedded within the upSKILL.map instrument activities. Ethical approval was granted by The Open University Human Research Ethics Committee. Where participants expressed interest and provided specific consent, collective attribution was offered in published outputs — including the think piece article and video clip — enacting a data sovereignty commitment in which contributors are recognised as co-producers of knowledge rather than research subjects.

**Figure 3.**
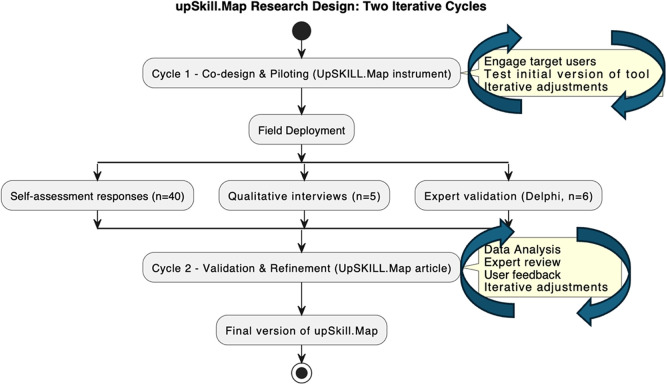
upSKILL.map Phase-1 research design: two iterative DBR cycles. Source: Okada, 2025a.

The research unfolded in two iterative cycles ( Figure 3):

• **Cycle 1: Co-Design and Piloting**. Target users (n = 8) reviewed the draft 8Cs competencies model and upSKILL.map instrument alongside the EU Research Competence model ( EC, 2025a), EU Research Manager Competence model ( EC, 2025b) and UN Sustainable Development Goals ( UN, 2023). Based on feedback regarding clarity and relevance, five key adjustments were made: converting the scale to a 4-point metric, rewording seven items, restructuring domains, expanding behavioural anchors, and repositioning “Translating” to “Catalysing”.
• **Cycle 2: Field Deployment and In-Context Validation**. The refined instrument was deployed across four doctoral programmes at The Open University (8 May–8 July 2025). Data included the upSKILL.map self-assessment with Likert-scale and reflective components (n = 40), follow-up interviews examining usability and relevance (n = 5), and a two-round Delphi-style consultation with six external experts. Experts evaluated item relevance, alignment, and completeness, followed by anonymised synthesis and consensus refinement. These three data sources including self-assessment, interviews, and expert consultation together supported content validity, reliability, and contextual applicability.

### 3.2 Participants and sampling

While the CARE–KNOW–DO principles are intended to inform researcher development across career stages, this Phase-1 study focuses on doctoral and early-career researchers as a critical leverage point for shaping future research culture. Recruitment for Cycle 2 was conducted within the OU’s doctoral community, which comprises around 621 postgraduate research students (approximately 308 studying full-time and the remainder part-time, combining research degree study with work) and roughly 100 supervisors and Early Career Researchers (ECR).

We included supervisors and early-career researchers (ECRs) to ensure perspectives from more established and experienced researchers who coordinate, design, deliver, and support doctoral training, as well as to capture their views on the developmental needs of doctoral students and ECRs. Researchers were invited on a voluntary basis through emails circulated by the doctoral schools and the heads of faculty, who supported dissemination via internal mailing lists. These invitations outlined the study’s focus on doctoral education and researchers’ professional development for addressing global challenges and provided information about METEOR project, without additional incentives. Initial direct invitations yielded 10 participants (<2%). A further 30 participants were recruited voluntarily through purposive and snowball sampling across four doctoral programmes in the OU, supported by our research group and supervisor nominations from the OU’s Open Societal Challenges programme, enabling the study to foreground the voices of those willing to engage in discussion of these issues. Demographic data ( Table 5) (career stage, ethnicity, gender, disability, first language, knowledge field and faculty) were collected to examine inclusivity patterns, assess contextual representativeness, and enable intersectional checks.

**Table 5.** Participant Demographics *(n = 40).*

Category	Subcategory	n	%
**Current Career Stage/Role**	Research Student	19	47.5
	Research Associate	3	7.5
	Research Fellow/Lecturer	8	20.0
	Senior Researcher/Senior Lecturer	5	12.5
	Professor/Director	3	7.5
	Other academic role	1	2.5
	Director	1	2.5
**Nationality**	Europe	22	55.0
	Africa	11	27.5
	Other continents (combined)	7	17.5
**Gender**	Female	26	65.0
	Male	13	32.5
	Non-binary	1	2.5
**Age Group**	40–49	13	32.5
	50–59	10	25.0
	30–39	7	17.5
	Other/not stated	10	25.0
**Ethnicity**	White/Caucasian	22	55.0
	African/Black	9	22.5
	Asian	4	10.0
	Mixed/multiple ethnicities	3	7.5
	Other/not stated	2	5.0
**Disability/Accessibility Needs**	Yes	7	17.5
**Disciplinary Area**	Wellbeing, education, language	15	37.5
	STEM	8	20.0
	Arts/social sciences	7	17.5
	Business/marketing	6	15.0
	Other/not stated	4	10.0
**Career Aspirations**	Academic progression	17	43.0
	Professional/industry	12	30.0
	Senior research management	7	17.5
	Other	4	10.0

Direct email invitations via four doctoral schools and faculty heads yielded 10 participants (<2% response rate)—a finding consistent with an aspiration–practice gap in engagement with reflective professional development, though not sufficient on its own to establish this relationship.

Subsequent snowball recruitment added 23 additional participants from across all four faculties, a 230% increase with zero incentives, rather than clustering in a single department. This cross-faculty activation suggests that eco-outwards commitment may be latent and peer-mobilizable rather than individually-driven, supporting the Phase-2 focus on institutional structures and community formation.

The study intentionally centres researchers interested in societal challenges, sustainability, global development and futures literacy themes—a constituency whose perspectives often remain marginalised in mainstream researcher-development frameworks. Within this self-selected group, the sample exhibits balanced representation across three profiles: eco-outwards research practitioners – eco-researcher (n ≈ 13), already actively engaged in global challenges; developing researchers (n ≈ 14), expressing eco-outwards commitments but reporting limited institutional support; and curious seekers (n ≈ 13), new to responsible innovation and sustainability-oriented research but motivated to develop these competencies.

This roughly equal distribution across experience levels strengthens analytical depth within the target population, while the voluntary, self-selected nature of participation limits generalisation to the wider researcher population. The findings therefore represent the perspectives and priorities of researchers who actively chose to engage with this agenda. As lead developer of upSKILL.map, the first author’s insider position was managed through independent Delphi review, dual coding, and participant feedback loops embedded across both DBR cycles.

The study was approved by The Open University’s Human Research Ethics Committee (HREC 2025–0889-4). Collecting self-assessment data in doctoral settings raises ethical concerns about deficit labelling and power relations between students, supervisors, and institutions. To minimise these risks, upSKILL.map was implemented as a semi-structured reflective activity—including a think piece for in-depth personal reflection—with all responses anonymised prior to analysis. Participants received clear information by email, including a brief explanatory summary and project website link, emphasising confidentiality, voluntary participation, and the self-reflective/developmental purpose (non-evaluative/non-scored) of the instrument. upSKILL.map was explicitly framed as a voluntary piloting activity, supported by informational materials and embedded consent procedures. All participants received copies of their individual data and a newsletter with the global study analysis. In addition, supplementary recorded interviews (n = 5) were conducted with participants who provided explicit informed consent for research and for attribution—with authorship acknowledged—in a video clip for open educational resources (OER).

### 3.3 Data analysis and open science commitments

Data analysis proceeded in two phases, combining quantitative and qualitative strands in an explanatory-sequential design.

Quantitative Analysis: Descriptive statistics and EFA were used to identify latent clusters of research competencies. The quantitative analysis provided an empirically grounded, inductive view of the competency landscape without imposing a priori structures. Sampling adequacy and factorability were checked with results indicating that the data were suitable for EFA despite the modest sample size (See §4.1).

Qualitative analysis: All 40 think pieces were analysed using a deductive–inductive thematic approach. These were independently coded by two researchers against a codebook derived from the CARE–KNOW–DO principles and the 8Cs model (deductive), with discrepancies iteratively refined through discussion of negative cases and emergent themes (inductive), documenting decisions in an analytical audit trail. Following Braun and Clarke’s (2019) reflexive thematic analysis approach, we did not calculate a κ coefficient or percentage agreement, as our focus was on rich, interpretive engagement with the data rather than quantifying coder similarity; disagreements were resolved through negotiated consensus, supporting trustworthiness and transparency. This is consistent with methodological guidance that cautions against conflating reliability with rigour in interpretive research: high κ can be achieved by narrowing codes to the point of losing conceptual richness, whereas low κ may reflect appropriate sensitivity to complexity ( Braun & Clarke, 2019). AI-assisted linguistic analysis was used as a supplementary epistemic check — not to replace interpretive judgement — to surface outliers and confirm the stability of key themes across participants, interviews, and expert consultations, which were coded using the same framework (see §4.2).

Integration and meta-inference: Integration followed an explanatory-sequential logic: quantitative patterns framed the qualitative interpretations, which were then used to re-examine the findings. Joint displays aligned factor scores, self-prioritised competencies and coded narratives, so that points of agreement, complement, and disagreement could be assessed directly rather than assumed.

**Figure 4.**
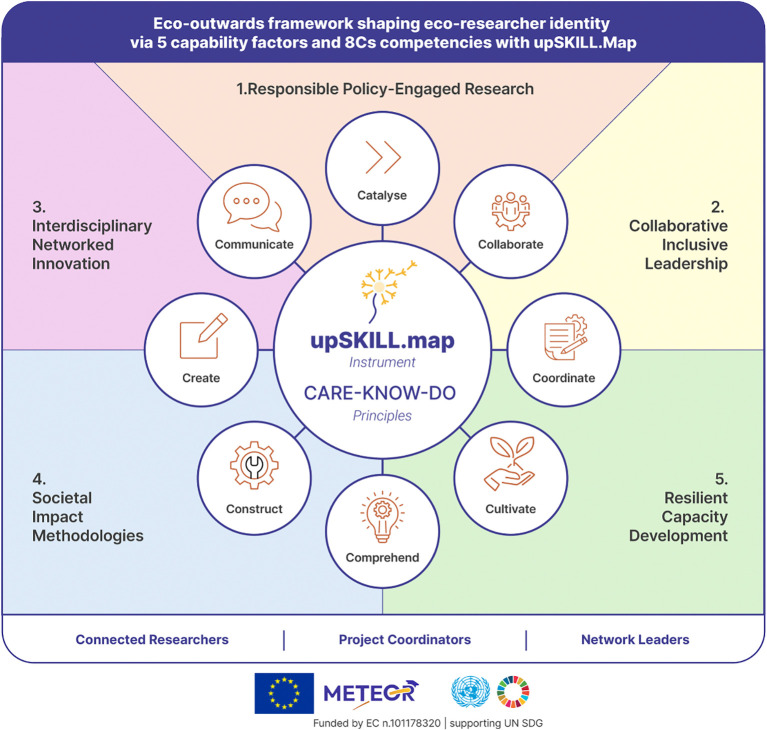
Eight competency domains (8Cs), based on CARE–KNOW–DO principles, clustering into five capability factors of eco-outwards researcher development. Source: Okada, 2025a.

To contextualise statistical findings, as detailed in the instrument design ( §2.3), participants were asked to indicate their involvement in research addressing global challenges—specifically, their contributions to the SDGs, advancement of knowledge toward critical problems, policy influence, and other significant pathways. Analysis of these responses, presented in Table 6 (Mechanisms of Research Impact), reveals how the 8Cs competency domains map onto authentic research practice. The five-factor structure ( Table 8; Figure 4) showed broadly consistent item relevance across different career stages in this cohort. Given the heterogeneity of the sample, noticeable between-stage variation might have been expected; its absence suggests the clustering reflects a pattern that is stable enough in Phase-1 to warrant confirmatory testing at scale. Together, these quantitative, qualitative, and integrative analytical strands supported the meta-inferences that underpin the institutional recommendations in §5.5.

**Table 6.** Mechanisms of research impact.

Impact Mechanism	% (n = 40)	Doctoral students’ and ECRs’ Example Projects/Themes	No. Students (out of 23)
(1) Advancing knowledge to address global challenges	22/40 = 55.0%	Disaster risk reduction; decolonising death & grief studies; OpenSTEM Africa; genetics; climate change education; sustainability	13
(2) Social innovation products	21/40 = 52.5%	AI apps for migrants; dialogic teaching; digital/accessible platforms; teacher development; inclusive STEM	10
(3) Shaping policy or regulation	17/40 = 42.5%	Workplace reproductive rights; DfE/Welsh policy; democracy; transport accessibility; AI/education regulation	7
(4) Major grant-funded initiatives	15/40 = 37.5%	CONNECT 2030 (EU); GCRF/British Academy; UKRI Future Leaders; Fleming Fund; Horizon Europe	8
(5) Other	7/40 = 17.5%	Ethics in digital design; green entrepreneurship; curriculum coding/ computational thinking; teaching professional development	3
(6) None of the above	8/40 = 20.0%	(No current involvement in above mechanisms)	7

In line with Open Research Europe’s commitment to open science ( Barnes & Weller, 2017) and to advance participatory research principles embedded in our DBR approach, the full upSKILL.map instrument (items, guidance notes, and coding approach), supplementary materials, open educational resources, videos, and dataset were made available via the UK METEOR ORDO website for institutional adaptation across contexts. During the review process, preliminary findings were shared with participants through research briefs to support collaborative feedback and refinement, ensuring the development process remained iterative and co-designed.

### 3.4 Trustworthiness and limitations

Rigour was ensured through three complementary strategies. First, triangulation combined participant reflections, expert review from the Delphi consultation ( Linstone & Turoff, 2002; Dalkey, 1969), and theoretical foundations mapped across the two DBR cycles, cross-validating findings and reducing single-source bias. Second, iterative DBR cycles of piloting and refinement supported credibility and confirmability throughout instrument development ( Lincoln & Guba, 1985). Third, grounding upSKILL.map in international frameworks—the EU Research Competence Framework ( RMComp; EC, 2025a) and the UN Sustainable Development Goals—strengthened both conceptual validity and applicability across diverse research contexts.

This Phase-1 study has four limitations, each acknowledged and mitigated.

Sample size. The sample (n = 40) is modest relative to large-scale validation studies; however, design-based research prioritises contextual depth over breadth ( Anderson & Shattuck, 2012; detailed in §3.2). The 28-item instrument retains a participant-to-item ratio of approximately 1.4:1, below the conventional thresholds for confirmatory factor analysis ( Tabachnick & Fidell, 2013; Fabrigar *et al*., 1999). The analytic implications of this ratio are addressed in §4.1.3, and Phase-2 multi-institutional replication (target n > 200) is designed to remedy the sample-size constraint.

These indicators are broadly consistent with the conditions under which de Winter *et al*. (2009) showed that EFA can yield reliable results for N below 50, corroborating evidence from independent sources is discussed in §4.2, and; full psychometric indicators are reported in Table 7. Accordingly, all findings should be treated as exploratory and institution-bound, not intended to support generalisation beyond the Open University context until multi-site replication is complete in Phases 2 and 3.

Single-institution focus. The study is grounded in the Open University’s organisational culture and disciplinary ecology, which limits cross-institutional transferability at this stage. However, the sample achieved disciplinary diversity across the university spectrum, providing insights into how different research traditions understand and prioritise eco-outwards competencies. This single-institution baseline serves as a reference model for Phase-2 multi-site replication across a minimum of three European partner institutions and Phase-3 sector-wide deployment, at which point confirmatory factor analysis (CFA) and cross-cultural measurement invariance testing will be conducted.

Supervisor network bias. All non-doctoral researchers in the sample were supervisors or recruited via supervisor networks, potentially biasing perspectives toward institutional leadership views. This is acknowledged and shapes interpretation of findings about institutional barriers and enablers; Phase-2 recruitment will explicitly target non-supervisory research staff to address this imbalance.

Social desirability bias. Self-reported data carries an inherent risk of socially desirable responding. This was mitigated through: (a) voluntary participation with no institutional incentives; and (b) open-ended think piece questions that generated rich qualitative evidence revealing institutional constraints alongside individual aspirations, reducing the risk that responses reflect only idealised self-presentation.

The factor loadings and ratings reported here reflect how participants positioned themselves in relation to each competency indicator — that is, the degree to which they perceived each item as relevant to their current practice. These findings reflect perceived relevance grounded in reflective experience and forward-looking aspiration, rather than individual performance. The distinction between perceived relevance and demonstrated competence will be addressed through expanded measurement approaches in the next phase of instrument development.

Taken together, the breadth of disciplinary experience in Phase-1, the coherent psychometric properties of the instrument, and the transparent multi-phase validation trajectory ( Boateng *et al*., 2018) provide a robust foundation for testing generalisability at scale. The limitations identified here are not disqualifying; they define the scope of Phase-1 claims and the agenda for Phases 2 and 3.

## 4. Findings

This section reports what the Phase-1 data can and cannot show. The quantitative analyses (n = 40) yield a tentative five-factor structure and item-level priority patterns; the qualitative analyses surface how a self-selected cohort of Open University researchers interprets and enacts eco-outwards competencies. Findings are therefore descriptive of this cohort and instrument-internal rather than generalisable, and the theoretical implications are taken up separately in §5.

### 4.1 RQ1: Structural context and coherence of the 8Cs model

Quantitative and qualitative data were analysed in an explanatory-sequential design ( Creswell & Plano Clark, 2018). EFA first identified the underlying factor structure of the 28-item instrument; qualitative interview data (n = 5) and expert consultation (n = 6, Delphi-style) subsequently triangulated and refined the factor labels and interpretations. This approach ensured that quantitative statistical patterns were grounded in lived researcher experience and aligned with international researcher-development standards.

***4.1.1 Context: Research on global challenges***

Participants were asked within the upSKILL.map instrument (see §2.3: Instrument Structure and Data Availability section for the complete questionnaire instrument) to indicate their current involvement in research addressing global challenges. This contextual item, administered alongside the 32 8Cs skill-rating items (Part 2), served to map how the competency model relates to authentic research practice. Specifically, participants selected from a set of research impact activities aligned with sustainability and societal benefit:

• Contributing to the Sustainable Development Goals (SDGS),
• Advancing knowledge to address critical global challenges,
• Influencing policy or regulation,
• Pursuing major grant-funded initiatives,
• Engaging in other significant research pathways
• No current involvement in the above

This contextual question investigates the paradigm shift described in the Introduction: it grounds the 8C model in evidence of how researchers engage with impact, equity, and sustainability in real-world practice. Engagement with impact was reported across a range of contexts. While all participants were based within a single UK university, the settings in which they carried out their research were global, spanning Sub-Saharan Africa, Europe, Latin America, and Asia. Examples included inclusive literacy strategies in Africa, participatory climate heritage projects in Europe, assistive AI tools for migrants in Latin America, and genomic data-sharing for public health in Asia. The distribution of responses reveals the diversity of mechanisms through which our sample demonstrates eco-outwards research engagement.

The 40 participants represented a diverse range of disciplinary areas within the institution. They identified six main mechanisms of research impact ( Table 6): advancing knowledge to address global challenges (55%), creating social innovation products (52.5%), shaping policy and regulation (42.5%), contributing to major grant-funded initiatives (37.5%), and, to a lesser extent, other areas (17.5%) or none of the above (20%). More than half described innovating beyond traditional research outputs, and over a third were engaged in externally funded projects.

Although situated within one institution, the diversity of participants’ fields, regions of research practice, and thematic commitments provides insight into how researchers navigate impact at the intersection of academic rigour and social relevance. Cross-cutting themes included digital transformation, inclusive development, co-creation with communities, and open innovation for sustainable change. Most projects were aligned with Quality Assurance (QAA, 2020), the SDGs ( UNESCO, 2017)—particularly SDG 4 (Quality Education), SDG 10 (Reduced Inequalities), and SDG 17 (Partnerships for the Goals)—underscoring the strong outward-facing orientation of the sample.

***4.1.2 Overview: Descriptive statistics***

Descriptive statistics characterised perceived relevance of each 8Cs competency item and informed subsequent psychometric checks (item screening, correlations, EFA, see Appendix 1–3).

Following screening, four poorly performing items were removed (4.1 Designing rigorous research methodologies; 5.1 Understanding/analysing complex systems; 7.2 Balancing competing demands/priorities; 5.4 Generating creative solutions to complex problems). In the retained 28-item solution, **communalities ranged from 0.24–0.82 (M = 0.52, SD = 0.16)**, indicating moderate–strong factor representation while preserving specificity—ideal for Phase-1 contextual analysis.

Participants rated the relevance of a range of research-related competencies to their current roles using a 1–4 scale (very low, low, moderate, high). The most highly valued competencies were explaining complex ideas to diverse audiences (M = 3.58), building professional networks (M = 3.60), applying ethical principles (M = 3.62), and critically evaluating evidence (M = 3.65). These underscore the centrality of evidence evaluation, ethics, and professional networking in participants’ work. Most competencies scored above moderate relevance (M > 3.0), indicating broad alignment with daily responsibilities. However, some—including engaging policymakers (M = 2.95), leading research teams (M = 2.88), and practising intercultural communication (M = 2.78)—were seen as less relevant, with greater variation across responses. This points to uneven applicability depending on role and suggests a need for more targeted development in leadership, policy engagement, and cross-cultural competencies to fully support diverse career pathways.

In terms of item-level correlation and scale coherence, the item-level Pearson correlation analysis for the 8Cs competency set (N = 40; 28 items) revealed consistently strong within-domain correlations, particularly in clusters such as collaboration and communication (r ≈ 0.65–0.85). For instance, “Working in teams across disciplinary boundaries” and “Contributing to develop a team project” exhibited a correlation of approximately 0.80, consistent with the internal coherence of these constructs.

Moderate to high cross-domain correlations (r ≈ 0.40–0.70) were also observed, suggesting that research-related competencies are perceived holistically. This pattern provides preliminary evidence supporting the internal coherence of the instrument in capturing integrated professional skillsets. While several inter-item correlations exceeded 0.75, indicating a high degree of internal consistency (α = 0.963), this also flags a minor risk of redundancy—an acceptable trade-off in early-stage instrument development where content breadth is essential.

Mann–Whitney U tests comparing Research Students with Research Fellows/Lecturers, Senior Researchers/Senior Lecturers, and Professors/Directors revealed no statistically significant differences across any of the 28 competency items (all p > 0.05), with one near-borderline result noted for competency_1 in the Senior Researcher/Senior Lecturer comparison (p = 0.075) (See Appendix 4). This pattern of non-significance is consistent with broadly shared perceptions of research competency relevance across career stages ( Brown *et al*., 2024), though small subgroup sizes limit statistical power and warrant interpretive caution. Nonetheless, the absence of systematic divergence across groups supports the cross-stage applicability of the 8Cs framework, providing a consistent foundation for the exploratory factor analysis reported below.

***4.1.3 Findings: Exploratory factorial analysis and technical evaluation***

Although the sample size (n = 40) is below conventional recommendations for exploratory factor analysis, its use is a deliberate methodological choice for Phase-1, context-bound validation within a design-based research framework, which prioritises theoretical coherence and contextual sensitivity over statistical generalisation.

Psychometric adequacy was first assessed to confirm the suitability of the dataset for exploratory factor analysis. As shown in Table 7, the correlation matrix was suitable for exploratory data reduction: the Kaiser–Meyer–Olkin measure indicated acceptable sampling adequacy (KMO = 0.732), Bartlett’s test of sphericity was significant (χ ^2^(378) = 1,152.93, p < .001), internal consistency was high (Cronbach’s α = 0.963), and total variance explained was substantial (75.69%), a level consistent with a well-structured instrument-development pilot.

**Table 7.** Psychometric Indicators (n = 40).

Metric	Value	Interpretation
Kaiser–Meyer–Olkin (KMO)	0.732	Acceptable sampling adequacy
Bartlett’s Test of Sphericity	χ ^2^(378) = 1152.93, p < .001	Correlation matrix factorable
Cronbach’s α (28 items)	0.963	Excellent internal consistency
Total Variance Explained	75.69%	Phase-1 descriptive data reduction (PCA), extraction-method-dependent
Participant-to-item ratio	1.4:1	Acceptable for exploratory, context-bound validation
Mean items per factor	5.0	Adequate factor representation

The factor representation (mean 5.0 items per factor) was adequate for interpretation. The participant-to-item ratio (1.4:1) is below conventional thresholds for confirmatory validation ( Tabachnick & Fidell, 2013; Fabrigar *et al*., 1999) and is the reason the solution is treated as descriptive rather than confirmatory (§4.1.3). This framing — separating adequately supported diagnostic elements from those requiring larger-sample confirmation — is offered as a methodological template for Phase-1 pilots and small-cohort studies where confirmatory thresholds cannot be met but exploratory clustering is still informative.

Principal Component Analysis (PCA) with Varimax rotation and Kaiser normalisation ( Tabachnick & Fidell, 2013; Kaiser, 1974) was used as the primary extraction method, consistent with the Phase-1 aim of variance-maximising descriptive data reduction during instrument development ( Gorsuch, 1983; Velicer & Jackson, 1990; Costello & Osborne, 2005). Following the four-item screening reported in §4.1.2, the Kaiser (eigenvalue > 1) criterion returned a five-component solution that converged in eight iterations ( Table 8), accounting for 75.69% of total variance:

• Component 1 (Responsible policy-engaged research): 19.83%
• Component 2 (Collaborative inclusive leadership): 16.23%
• Component 3 (Interdisciplinary networked innovation): 14.47%
• Component 4 (Societal impact methodologies): 13.47%
• Component 5 (Resilient capacity development): 11.69%

**Table 8.** Exploratory factor analysis revealing 5 clusters integrating 8C skills.

Rotated Component Matrix ^a^					
	Component				
	1	2	3	4	5
**1: Responsible Policy-Engaged Research**					
@5.2.Critically evaluating evidence for recommendations	.825				
@4.4.Applying ethical approaches and integrity principles	.808				
@7.3.Managing risks ethics and quality assurance	.782				
@8.3.Promoting knowledge sharing to empower communities	.745				
@2.3.Engaging policymakers with research findings	.661				
@8.2.Translating findings into policy recommendations	.633			.579	
@2.4.Practicing intercultural communication via multimedia	.476				
**2: Collaborative Inclusive Leadership**					
@2.1.Explaining complex ideas to diverse audiences		.816			
@1.1.Working in teams across disciplinary boundaries		.753			
@1.2.Contributing meaningfully to develop a team project		.683			
@1.4.Promoting inclusion and diversity in research teams		.609			
@7.4.Leading teams to meet research goals and deadlines		.503			
@5.3.Synthesizing information from diverse sources		.483			
**3: Interdisciplinary Networked Innovation**					
@2.2.Building and maintaining professional networks			.823		
@6.3.Combining findings to develop new knowledge			.721		
@6.2.Challenging accepted ideas using innovative approaches			.679		
@6.4.Adapting research direction when initial methods fail			.672		
@3.2.Participating in research groups or communities			.572	.484	
@6.1.Identifying links between different disciplines or expert views			.524		
@3.3.Organizing academic events and learning opportunities			.494		
**4: Societal Impact Methodologies**					
@4.2.Conducting interdisciplinary literature reviews and synthesis				.748	
@4.3.Implementing mixed and multimethods approaches				.609	
@8.1.Driving open research projects for equity and inclusion				.595	
@8.4.Evaluating research impact for a sustainable future	.519			.576	
**5: Resilient Capacity Development**					
@1.3.Resolving conflicts constructively		.479			.750
@3.4.Fostering a culture of continuous improvement					.744
@3.1.Supporting the development of research colleagues					.724
@7.1.Planning and managing resources effectively				.562	.639

Extraction Method: Principal Component Analysis.

Rotation Method: Varimax with Kaiser Normalization.

^a^ Rotation converged in 8 iterations.

Component loadings ranged from 0.476 to 0.825, with all items loading above 0.45 on their primary component and cross-loadings remaining below this threshold, supporting differentiation and interpretability of the clustering.

Because the Kaiser rule is known to over-extract at small sample sizes ( Zwick & Velicer, 1986), three convergent diagnostics were applied to interrogate the component count. The **scree plot** (Appendix 1) showed a pronounced elbow after Component 1, with eigenvalues declining smoothly through Components 2–7 without a secondary inflection; by the Cattell (1966) criterion this supports a one- to two-component solution. **Parallel analysis** ( Horn, 1965; Glorfeld, 1995), comparing observed eigenvalues against the 95th percentile of 1,000 Monte Carlo simulations of random data, found that only Component 1 exceeded the random-data threshold (actual eigenvalue = 14.698 vs. random 95th percentile = 3.330); Components 2–5 did not exceed their corresponding thresholds. A **common-factor robustness check** using Principal Axis Factoring (PAF) with Oblimin rotation converged with the PCA on four of five components (Responsible Policy-Engaged Research, Interdisciplinary Networked Innovation, Societal Impact Methodologies, and Resilient Capacity Development replicated with stable item composition), while Collaborative Inclusive Leadership showed method-dependence, its items redistributing across clusters under oblique rotation. The corresponding fifth PAF factor returned a rotation sum of squared loadings of 1.77 — an order of magnitude smaller than the remaining four (6.7–10.1).

Taken together, these diagnostics indicate that only Component 1 is robustly supported as an independent latent dimension at n = 40, a pattern consistent with a general-factor-plus-specific-facets organisation ( Reise *et al*., 2010; Rodriguez *et al*., 2016) in which a strong eco-outwards orientation accounts for approximately half of item variance, with four specialised subsidiary clusters and one weaker, method-sensitive dimension. Despite this statistical caution, the five-component PCA solution was retained as the primary Phase-1 descriptive result on three interpretive grounds:

a) it aligns with the theoretically-derived 8Cs structure ( §2.2);
b) it produces results consistent with the independent qualitative analysis — both the Delphi-style expert consultation (n = 6) and the thematic analysis of researcher think pieces (n = 40) produced five interpretively coherent clusters matching the PCA components ( §4.2), providing triangulation support that statistical diagnostics alone cannot establish; and
c) it provides the organising structure for the institutional-planning framework in §5.5 and Table 10.

The five-component structure is therefore presented as a theoretically-informed descriptive clustering and a baseline specification for Phase-2 confirmatory refinement, not as a validated latent-factor model. Phase-2 will test this baseline against competing structural specifications (single-factor, bifactor, hierarchical) with a pre-registered multi-institutional sample (n > 200; see §5.4). This positioning is consistent with the DBR programme’s iterative logic, in which Phase-1 pilot clustering surfaces candidate structures that Phase-2 confirmatory testing subsequently refines or restructures at scale.

### 4.2 RQ2: Prioritised eco-outwards competency factors

The five-factor structure identified through exploratory factor analysis was used to organise the qualitative phase: factor scores informed the selection of illustrative cases, guided the development of the coding frame, and shaped the clustering of themes around responsible research, collaboration, networking, methodological breadth, and resilience. Quantitative patterns showed that all five factors scored above the midpoint, with Factor 1 (responsible and policy-engaged research) highest and Factor 5 (resilient capacity development) lowest — suggesting strong ethical awareness but weaker support for sustaining work in complex contexts. Together, quantitative and qualitative strands revealed an aspiration–practice gap and highlighted resilience as a priority area for development. Qualitative narratives were then used to interpret and name each factor through a CARE–KNOW–DO lens: examining how researchers emotionally engage with challenges (CARE), understand systemic barriers (KNOW), and enact change (DO) within institutional contexts. This iterative movement between quantitative structure and qualitative depth supported the interpretability of both the instrument and its theoretical framing at Phase-1, pending Phase-2 confirmatory testing.

### Factor 1: Responsible Policy-Engaged Research

EFA grouped a cluster of seven interrelated skills: critical evaluation, ethical principles, risk management, community empowerment, policymaker engagement, evidence-to-policy translation, and multimodal intercultural communication. With regard to research connected to policy (see Table 6). Three skills clustered together, indicating that policymakers, communities, and researchers must be engaged throughout the research process through knowledge exchange, research agenda co-creation, and research-informed recommendations. Importantly, “evidence-to-policy translation” is not conceptualised as a linear, post-research activity, but as an iterative and negotiated process embedded in contexts of policy design, use, and implementation.

Thematic analysis of open-ended responses ( Box 1) captured six perspectives from students, researchers, and academic leaders. A recurring theme was the *aspirational–practical gap*: the tension between strong commitments to responsible research and the real-world challenges of implementation. Many students expressed motivation to pursue societal impact, ethical integrity, and policy relevance—echoing the skills identified in this factor. For example, they called for greater industry collaboration, critical engagement with emerging ethical issues such as AI, and research that delivers tangible long-term benefits.

Box 1. **Thematic analysis linked to Factor-1 Responsible Policy-Engaged Research.**

**Table:** 

Themes	Most Representative Quote	Stakeholder
1. Aspirational vs. Practical Gap	“I have always struggled to align my work with the public good or sustainability as it’s been so ‘blue skies’.”	Leadership [L5]
2. Skills and Training Deficits	“Structured training in leadership or policy engagement is more limited … others lack the mentoring or applied contexts to do so confidently.”	Research Student [DS1]
3. Institutional Constraints	“Without external funding or pre-established relationships … it is very difficult to pursue real world social impact.”	Research Fellow [R11]
4. Ethical Imperative	“When research on inclusion does not translate into real-world impact, it contributes to marginalisation and discrimination.”	Research Student [DS6]
5. Systemic Change Needed	“Embed real-world engagement into the core of doctoral education not as an add-on, but as a fundamental part of research design.”	Research Student [DS5]
6. Continuous policy engagement and participatory approaches	My role demands deep comprehension of sustainable development frameworks, green economy trends, and start-up dynamics. I continuously engage in reading, research synthesis, and environmental scanning including policy frameworks and participatory regulation	Research Associate [DS4]

For example, statements such as “I have always struggled to align my work with the public good” were coded as tensions between aspiration and application, contributing to the theme “Aspirational vs practical gap” within Factor 1.

However, students frequently reported insufficient training and limited opportunities to develop the skills needed to translate research into policy or community outcomes ( *Skills and Training Deficits, Theme 2*). Across stakeholder groups, there was consensus on the importance of societal benefit and research integrity, yet frustrations emerged regarding institutional barriers such as restricted funding and rigid structures that inhibit applied engagement ( *Institutional Constraints, Theme 3*). Senior researchers emphasised the ethical imperative to ensure research contributes beyond academia ( *Ethical Imperative, Theme 4*), while calls for reform highlighted the need to embed real-world engagement more centrally within doctoral education ( *Systemic Change Needed, Theme 5*), supported by participants’ accounts of continuous policy engagement and participatory approaches with policymakers and communities (Theme 6).

Convergence between the quantitative and qualitative strands was strong for this factor. The three highest-loading items in Factor 1 — critical evaluation (.825), ethical principles (.808), and risk management (.782) — aligned with the three most recurrent qualitative themes: ethical imperative (Theme 4), aspirational–practical gap (Theme 1), and institutional constraints (Theme 3), each surfacing across all three career-stage groups. One divergence is worth noting: while the EFA positions intercultural communication as the lowest-loading item in this factor (.476), qualitative narratives rarely named it as a development priority, suggesting it may not belong in this cluster and warrants item-level review in Phase-2.

### Factor 2: Collaborative Inclusive Leadership

EFA identified a cluster of six interconnected skills centred on the ability to communicate complex ideas to diverse audiences, contribute meaningfully within research teams, and foster inclusive environments. These interpersonal and organisational competencies are critical for enabling productive and equitable collaboration in research contexts. Factor 2 received consistently high ratings, especially from students and lecturers, indicating that inclusive practices are both recognised and valued. However, participants also reported limited time for reflection, unequal decision-making power, and a lack of recognition for collaboration in promotion systems—echoing calls for systemic change to embed and reward inclusive leadership.

Thematic analysis ( Box 2) reinforced the centrality of these competencies but revealed gaps in practice. Students placed high importance on communication and peer networks, as both qualitative and quantitative data revealed (Theme 1), yet described persistent collaborative skills gaps (Theme 2) and institutional recognition barriers (Theme 3) that undermine their ability to contribute fully. Crucially, the high ranking of communication skills does not imply that the research itself lacks relevance or societal demand. On the contrary, participants explicitly framed societal impact as a non-negotiable component of modern scholarship. Aspirations for embedding inclusion and sustainability into research design (Theme 4) often remained unfulfilled, while wellbeing through connection (Theme 5) was seen as both a personal necessity and an institutional responsibility. The barrier is not a lack of meaningful research to share, but rather an opportunity-based gap where institutional structures have not yet caught up to researchers’ aspirations to be “accountable to wider society.

Box 2. **Thematic analysis linked to Factor-2 Collaborative Inclusive Leadership.**

**Table:** 

Themes	Most Representative Quote	Stakeholder
1. Communication and Peer Networks	“Communication and collaboration. These are essential not only for effective teamwork but also for translating research into meaningful outcomes and engaging with diverse stakeholders.”	Research Fellow (R8)
2. Collaborative Skills Gaps	“We also need more practical training - hands-on training... Skills such as research and analytical skills, academic writing and communication, time and project management, networking and collaboration.”	Research Student (DS3)
3. Institutional Recognition Barriers	“There is currently no way to account for how individual researchers and the ways they think impact their lab. I have a colleague who has contributed to nearly every research group informally, but she is not valued.”	Senior Researcher (SR4)
4. Inclusive Practice Aspirations	“Embed inclusion and sustainability into the structure of research design—not as optional themes, but as core values. A successful doctorate today should not only advance knowledge but make that knowledge usable, accessible, and accountable to wider society.”	Research Student (DS1)
5. Wellbeing Through Connection	“One important point we haven’t discussed is mental health and well-being. Many students face stress, isolation, or uncertainty during their PhD... Institutions should prioritise well-being support, foster peer communities, and normalise help-seeking.”	Research Student (DS2)

Participants possess the motivation to bridge research-practice gaps but lack the institutional space and time to develop these competencies confidently. While informal networks offer some support, the current framework often leaves students without the mentoring or applied contexts needed to translate complex ideas for a global job market.

Convergence between the quantitative and qualitative strands was strong for this factor. The highest-loading item — explaining complex ideas to diverse audiences (.816) — aligned directly with Theme 1 (Communication and Peer Networks), reinforcing that communication is both statistically central to this factor and experientially prioritised by researchers at every career stage. The remaining high-loading items — working in teams across disciplinary boundaries (.753) and contributing meaningfully to team projects (.683) — converged with Theme 2 (Collaborative Skills Gaps) and Theme 3 (Institutional Recognition Barriers), where participants described motivation to collaborate but insufficient formal structures to support it. One divergence is worth noting: wellbeing (Theme 5) emerged as a recurring qualitative concern — particularly among doctoral students describing stress, isolation, and the need for peer community — yet no item in the current 28-item scale directly captures researcher wellbeing or mental health support. This suggests wellbeing is an emergent participant priority that sits adjacent to collaborative leadership in lived experience but is not yet represented in the instrument’s item set and warrants consideration in Phase-2 item development.

### Factor 3: Interdisciplinary Networked Innovation

Exploratory Factor Analysis (EFA) identified a cluster of capabilities combining creative problem-solving, adaptability, and the sustained relationship-building needed to advance research agendas in complex and evolving contexts. These skills underpin cross-disciplinary collaboration and the ability to navigate emerging challenges. Factor 3 received moderate scores overall, with senior researchers rating these competencies more highly than students, likely reflecting experience-based confidence. Students reported restricted access to networks and difficulties engaging in intersectoral collaboration, underscoring the need for institutional strategies that democratise access to professional communities and innovation spaces.

Thematic analysis of qualitative responses ( Box 3) revealed strong proactive engagement with innovation and networking among students and early career researchers (ECRs). Activities included integrating AI and other digital tools into research workflows, building cross-disciplinary partnerships, organising academic events, and connecting with both academic and non-academic stakeholders. There was consistent demand for structured, credit-bearing opportunities in areas such as grant writing, professional exchanges, and industry or NGO collaborations, with a view to producing tangible and demonstrable outcomes.

Box 3. **Thematic analysis linked to Factor-3 Interdisciplinary networked innovation.**

**Table:** 

Theme	Most Representative Quote	Stakeholder
AI Integration and Digital Innovation	“Yes, I integrate AI tools into my research to enhance productivity, deepen analysis, organize data, and improve text quality. I use tools such as ChatGPT, Grammarly, Notion AI, Slack, Trello, Padlet, Copilot, Consensus, IRaMuTeQ, among others.”	Research Fellow (R8)
Cross-Disciplinary Collaboration	“Skills: create research outputs (e.g. workflows, datasets); communicate (e.g. writing tutorials and articles); collaborate (working with biologists and computer scientists).”	Research Student (DS13)
Professional Network Building	“Skills such as research and analytical skills, academic writing and communication, time and project management, networking and collaboration, teaching and presentation skills, and adaptability and career planning are key for doctoral completers.”	Research Student (DS3)
Grant Writing and Funding	“More hard skills (technologies, methodologies, techniques); a set of tangible outcomes beyond passing a viva and a thesis: publishing paper(s), grant writing, project management, budgeting, teaching duties, running conferences.”	Research Fellow (R7)
Industry and Real-World Partnerships	“Universities can better support career development by embedding practical training, creating stronger networks with industry and NGOs, and recognising non-traditional pathways as valid. PhD research can have tremendous societal impact when aligned with urgent challenges.”	Research Associate (R1)
Creative Problem-Solving	“I develop innovative lithium separation technologies, so Creating and Collaborating are the main 8C skills... training opportunities and support from my line manager has been crucial to learn and thrive in this new research area.”	Research Associate (R3)
Event Organization and Leadership	“I also contributed to the organisation of a large-scale conference which led to organising a post-graduate conference at the institution – all of which developed leadership skills and experience, offering a useful skillset within academia and beyond.”	Research Fellow (R4)
International and Global Networking	“Having the opportunity to conduct part of my doctoral programme abroad was transformative. It gave me a global perspective on education and encouraged me to engage more deeply with international debates on global issues.”	Research Fellow (R8)

Senior researchers emphasised the importance of sustained interdisciplinary dialogue and regular exchanges across departments, while institutional leaders focused on the strategic cultivation of external networks and the careful management of risks associated with openness in a challenging global climate, particularly regarding equity, diversity, and inclusion (EDI).

Convergence between the quantitative and qualitative strands was strong for this factor. The three highest-loading items — building and maintaining professional networks (.823), combining findings to develop new knowledge (.721), and challenging accepted ideas using innovative approaches (.679) — aligned with the three most prominent qualitative themes: Professional Network Building, Cross-Disciplinary Collaboration, and Creative Problem-Solving, each reported consistently across doctoral students, early-career researchers, and research fellows.

A notable emergent finding from the contextual research data is that AI tool use appeared as a cross-cutting capability across career stages — cited by doctoral students, research associates, and research fellows in relation to networking, knowledge synthesis, and creative problem-solving. Significantly, AI was not assessed as a standalone competency within the 8Cs instrument, reflecting its design intention: AI literacy is conceived as an enabling capability that cuts across and amplifies all eight competency domains rather than constituting a discrete skill. This finding suggests that a dedicated cross-cutting AI dimension — framed as augmentation of eco-outwards practice rather than a ninth competency — warrants consideration in Phase-2 instrument development and curriculum design.

Overall, these findings indicate that while researchers at all stages recognise the value of innovation and networking, opportunities to develop these capabilities ( Nussbaum, 2011) are often self-initiated or externally driven rather than embedded in core doctoral training. Embedding structured, well-supported pathways for professionalisation—paired with formal recognition of achievements—would strengthen capacity for innovation, adaptability, and engagement across career stages.

### Factor 4: Societal Impact Methodologies

EFA identified a cluster representing methodological breadth—ranging from interdisciplinary literature synthesis to mixed and multimethods application—with a strong emphasis on equity, inclusion, and sustainability. Rather than positioning impact as a post hoc outcome, this construct situates research within societal, environmental, and policy contexts, linking methodological choices to co-creation, participation, and reflexive evaluation of impact. In doing so, it connects interdisciplinary inquiry with tangible societal and environmental benefits, underscoring the potential of research to achieve meaningful impacts beyond academia.

Factor 4 scored well overall; however, findings revealed a persistent gap between the value placed on these skills and their consistent application in practice. Disciplinary silos, insufficient incentives, and the complexity of evaluating socially embedded impact remain significant barriers, reflecting Horizon Europe’s observation that interdisciplinarity is essential yet under-supported ( European Commission, 2023).

Thematic analysis of qualitative responses ( Box 4) revealed widespread ambition—particularly among students—to engage in interdisciplinary and societally engaged research that transcends traditional boundaries. Participants emphasised the importance of participant-led methods, cross-sector collaboration, and open research practices as means of working with communities and stakeholders across the research lifecycle, rather than informing them only at later stages. However, barriers emerge at multiple levels: students encounter siloed training and limited exposure to cross-field collaboration; mid-career researchers face workload imbalance and resource constraints; and senior academics point to the absence of institutional structures that systematically enable interdisciplinary and societally embedded initiatives.

Box 4. **Thematic analysis linked to Factor-4 Societal Impact Methodologies.**

**Table:** 

Theme	Most Representative Quote	Stakeholder
Mixed-Methods and Methodological Breadth	“Research training has supported my development of ethical and innovative research by introducing the multiple methods by which research can be participant-led with rich, multisensory, mixed-methods approaches.”	Research Student (DS19)
Equity, Inclusion, and Sustainability Focus	“If I could change one thing about doctoral education, it would be to embed inclusion and sustainability into the structure of research design—not as optional themes, but as core values. A successful doctorate today should not only advance knowledge but make that knowledge usable, accessible, and accountable to wider society.”	Research Student (DS1)
Cross-Disciplinary Collaboration	“Skills: create research outputs (e.g. workflows, datasets); communicate (e.g. writing tutorials and articles); collaborate (working with biologists and computer scientists) ... I occasionally use AI-based bioinformatics tools to explore datasets.”	Research Student (DS13)
Real-World Impact and Societal Benefits	“It’s frustrating sometimes, because I believe our research has so much potential to inform policy or support communities, but the academic system doesn’t always encourage or reward that kind of engagement. The skills I’ve found most essential aren’t just academic ones... being able to adapt, work ethically, and connect with people across disciplines feels more important than ever.”	Research Student (DS5)
Overcoming Disciplinary Silos	“I think the silo of disciplines contributes to this, particularly in AI, which has an enormous engineering focus. I think it’s a barrier for social innovation required to address societal challenges and global needs.”	Senior Researcher (SR4)
Literature Synthesis and Knowledge Integration	“Yes, I use AI tools primarily for literature reviews and marketing research. For example, I used Scite.ai and Connected Papers to map the evidence base around green entrepreneurship education, and tools like ChatGPT for drafting survey instruments and analyzing open responses.”	Research Associate (R1)
Environmental and Social Impact	“Universities can better support career development by embedding practical training, creating stronger networks with industry and NGOs, and recognising non-traditional pathways as valid. PhD research can have tremendous societal impact when aligned with urgent challenges like climate change and youth unemployment.”	Research Associate (R1)
Interdisciplinary Team Experiences	“Having the opportunities to work collaboratively with other doctoral students and higher education colleagues throughout my doctoral studies was key in developing transferable skills such as teamwork, leadership and time management.”	Research Fellow (R4)

Across stakeholders, there was clear recognition of the importance of embedding inclusion and sustainability into research design, strengthening cross-disciplinary collaboration, and ensuring research outputs deliver real-world benefits. Participants emphasised the value of diverse team experiences, internships, and industry or NGO partnerships to bridge disciplinary divides and enhance impact. Convergence between the quantitative and qualitative strands was strong for this factor. The three highest-loading items — conducting interdisciplinary literature reviews and synthesis (.748), implementing mixed and multimethods approaches (.609), and driving open research projects for equity and inclusion (.595) — aligned with the three most prominent qualitative themes in Box 5: Mixed-Methods and Methodological Breadth, Equity, Inclusion and Sustainability Focus, and Cross-Disciplinary Collaboration, each surfacing across doctoral students, mid-career researchers, and senior academics.

A cross-factor permeability finding is worth noting: environmental and social impact themes appeared prominently in Box 4 narratives — with participants framing impact as inseparable from methodological choices — yet the EFA loads impact evaluation (.576) primarily onto Factor 1 (Responsible Policy-Engaged Research) rather than Factor 4.

Overall, the findings suggest a shared commitment to interdisciplinary, socially responsible research, but also a structural deficit in the conditions needed for it to thrive. Addressing entrenched silos, funding gaps, and the lack of formalised pathways for collaboration is identified by participants as a key condition for enabling researchers to fully realise the societal and environmental potential of research competencies.

### Factor 5: Resilient Capacity Development

EFA identified a cluster of adaptive capacities essential for overcoming challenges, sustaining continuous improvement, and supporting colleagues. This construct spans time and resource management, conflict resolution, adaptability under uncertainty, and building a supportive research culture. Factor 5 was the lowest-scoring cluster, particularly among early career researchers, reflecting a gap between the acknowledged importance of resilience and the structured opportunities available to develop it. Many participants reported feeling unprepared for key skills—especially conflict resolution and resource optimisation—which are often acquired informally and late in a career, increasing the risk of burnout and attrition.

Thematic analysis ( Box 5) revealed that resilience manifests differently across career stages. Students and early-career researchers often rely on self-motivation and informal networks to navigate stress, isolation, and limited resources, describing their doctoral journey as largely self-directed. Mid-career researchers emphasised mentorship as critical to sustaining growth and meeting evolving demands, while senior academics underscored the need for adaptability in a changing higher education landscape, yet noted persistent gaps in formal training provision.

Box 5. **Thematic analysis linked to Factor-5 Resilient Capacity Development.**

**Table:** 

Theme	Most Representative Quote	Stakeholder
Managing Stress and Self-Doubt	“One important point we haven’t discussed is mental health and well-being. Many students face stress, isolation, or uncertainty during their PhD, especially in distance or part-time models. Institutions should prioritise well-being support, foster peer communities, and normalise help-seeking as part of healthy research culture.”	Research Student (DS2)
Time Management and Resource Optimization	“Time constraints of a 3-year funded phd means we have limited options in terms fitting every together; that is ensuring we complete in time puts a mental strain on us and our focus is usually also limited to finishing up the thesis.”	Research Student (DS4)
Conflict Resolution and Problem-Solving	“Personal drive and perseverance are most important because without this overcoming endless barriers is impossible... Only based on my own experience, academia is often very far removed from the real-world so impact can be a worthy aspiration but requires real-world experience to achieve.”	Research Student (DS8)
Self-Motivation and Self-Direction	“My research training has been a variable experience, predominantly self-driven which has made it challenging on occasion to know which path to follow or what to prioritise. Focusing on areas I have some knowledge of, and expanding on these has been most impactful.”	Research Student (DS8)
Mentorship and Peer Support	“I was fortunate to have supervisors who truly believed in my potential and consistently reminded me that I could exceed expectations through my commitment, organization, focus, and ability to communicate effectively... This kind of mentorship and inclusion in real, ongoing work developed in me a level of understanding that no formal program could have offered on its own.”	Research Fellow (R8)
Adaptability Under Uncertainty	“A successful researcher needs to be flexible, resilient and open-minded... Researchers have the skills; however, we are constrained by the current UK HE environment, which is downwardly focused, contracting and in real trouble.”	Leadership (L5)
Sustaining Long-Term Performance	“More ongoing support rather than little nibbles of short things. I wish I could still access vitae for example... One that can make a living and make a difference... I wish I felt more able to get a research job as this is proving hard.”	Research Associate (R2)
Building Supportive Research Culture	“Students and their supervisors are a synthesis of ideas, creativity, inspiration, positivity and sheer hard work! Generally, the more the student engages with their studies, the more the supervisor can help and support the student... Supervisors can help the student to take a more long-term view and help to reduce that awful panicky feeling when things go wrong.”	Leadership (L4)

Across stakeholders, common threads include the mental strain caused by compressed timeframes, the challenge of maintaining wellbeing under competing pressures, and the need for stronger institutional systems to sustain long-term performance. Participants described resilience as being built not only through personal drive and adaptability, but also through access to supportive supervisors, peer communities, and ongoing professional development. They also linked resilience to the cultivation of a healthy research culture—one that reduces panic during setbacks, normalises help-seeking, and values collaboration alongside individual achievement.

Convergence between the quantitative and qualitative strands for this factor was strong but took an unexpected form. Factor 5 was the lowest-scoring cluster quantitatively — with early-career researchers in particular rating these competencies as less relevant to their current roles — yet it generated the most emotionally charged qualitative content in the dataset. Box 5 contains the only themes in this study explicitly addressing mental health, burnout risk, time pressure, and isolation, with doctoral students describing their experience as ‘predominantly self-driven’ and expressing acute awareness of the absence of formal resilience support.

This quantitative-qualitative divergence is one of the study’s most substantive integration findings: researchers may systematically underrate resilience competencies on a relevance scale precisely because they experience them as personal struggles rather than recognised professional skills, effectively masking a structural provision gap that quantitative self-assessment alone cannot surface. Phase-2 should test whether reframing resilience items as institutional conditions rather than individual capabilities yields more differentiated responses across institutional context.

## 5. Discussion

The conceptual and methodological contribution of Phase-1 is not to generalise but to make Phase-2/3 generalisable. This conceptual and structural groundwork — an instrument with initial psychometric coherence, a calibrated competency language, and an identified set of institutional barriers — is what makes rigorous large-scale replication possible; without it, a multi-site study would lack the theoretical and measurement foundation needed to generate interpretable, comparable findings.

At the conceptual level (RQ1), our findings provide initial evidence supporting the coherence and context-specific robustness of the 8Cs model, warranting multi-site confirmation as a roadmap to enhance the eco-researcher identity. Empirically (RQ2), the five-factor structure and associated narratives reveal that researchers prioritise responsible, collaborative, and impact-oriented competencies but encounter uneven institutional support—particularly around resilience (Factor 5) and policy-engaged research (Factor 1).

These Phase-1 findings are consistent with a shift from competency-as-performance to competency-as-worldview a framing that warrants confirmation through multi-site replication, enabling individuals’ and institutions’ awareness as key fundamental factors for positioning ethics, sustainability, and justice as core research competencies rather than supplementary values ( Fischer & Wiek, 2022; Mezirow, 1997; Roy *et al*., 2025). This framing invites reconsideration of traditional frameworks that treat responsibility as individual choice ( Lee *et al*., 2022; Eraut, 1994) rather than structural professional requirement ( Owen *et al*., 2022; Boni & Gasper, 2012).

### 5.1 Integration of theoretical and empirical framework

The 8Cs model provides a conceptual map for reflection, curriculum design, and assessment, while the five Phase-1 factors offer a complementary lens for understanding how competencies cluster in practice:

1. **Responsible Policy-Engaged Research** ensuring ethical approaches, risk management, and community empowerment. This factor maps to the eco-outwards framework through the researcher’s commitment to responsible innovation ( Stilgoe *et al*., 2013; Owen *et al*., 2022) and anticipatory governance ( Dedeurwaerdere, 2024). Phase-2 validation should examine whether high scores correlate with observed engagement in responsible-research practices.
2. **Collaborative Inclusive Leadership** fostering diversity, inclusion, and synthesis of diverse perspectives. Grounded in epistemic justice and pluralism ( Walker & Boni, 2006; Owens & Brant, 2023), this factor reflects how eco-researchers engage with marginalised voices and challenge deficit models of expertise.
3. **Interdisciplinary Networked Innovation** enabling cross-sector networking, innovation, and eco-social impact. This connects to systems thinking ( Wiek *et al*., 2011; Horcea-Milcu *et al*., 2024) and transdisciplinary collaboration ( Dedeurwaerdere, 2024) essential for addressing interconnected global challenges.
4. **Societal Impact Methodologies** promoting open, equitable research and evaluation of impact for a sustainable future. Aligned with RRI principles ( Stilgoe *et al*., 2013) and the new UNESCO emphasis on research-education integration for the SDGs ( UNESCO, 2017).
5. **Resilient Capacity Development** supporting conflict resolution, adaptability, and resource management for sustained contributions in dynamic contexts.

These five factors—responsible research, collaborative leadership, interdisciplinary networking, methodological breadth, and resilience—together constitute a coherent architecture for the eco-researcher role by integrating the CARE–KNOW–DO principles: commitment (CARE) to sustainability and justice, systems-level understanding (KNOW), and capacity to catalyse change (DO). The dual structure captures both comprehensive competency coverage (8Cs) and integrated, priority competency clusters (five factors), enabling targeted professional development while keeping eco-outwards commitments inseparable from practice.

The 8Cs offer scaffolding for comprehensive curriculum design and reflective development, whereas the factors highlight priority areas for intervention. Critically, all five factors embody the eco-outwards orientation in that none separates technical skill from ethical purpose ( Cole *et al*., 2023; McAlpine & Amundsen, 2018). Together, these constructs form an empirically grounded, career-spanning model for strengthening individual and systemic research capacity, offering a transferable framework for policy, training, and institutional strategy.

**Figure 5.**
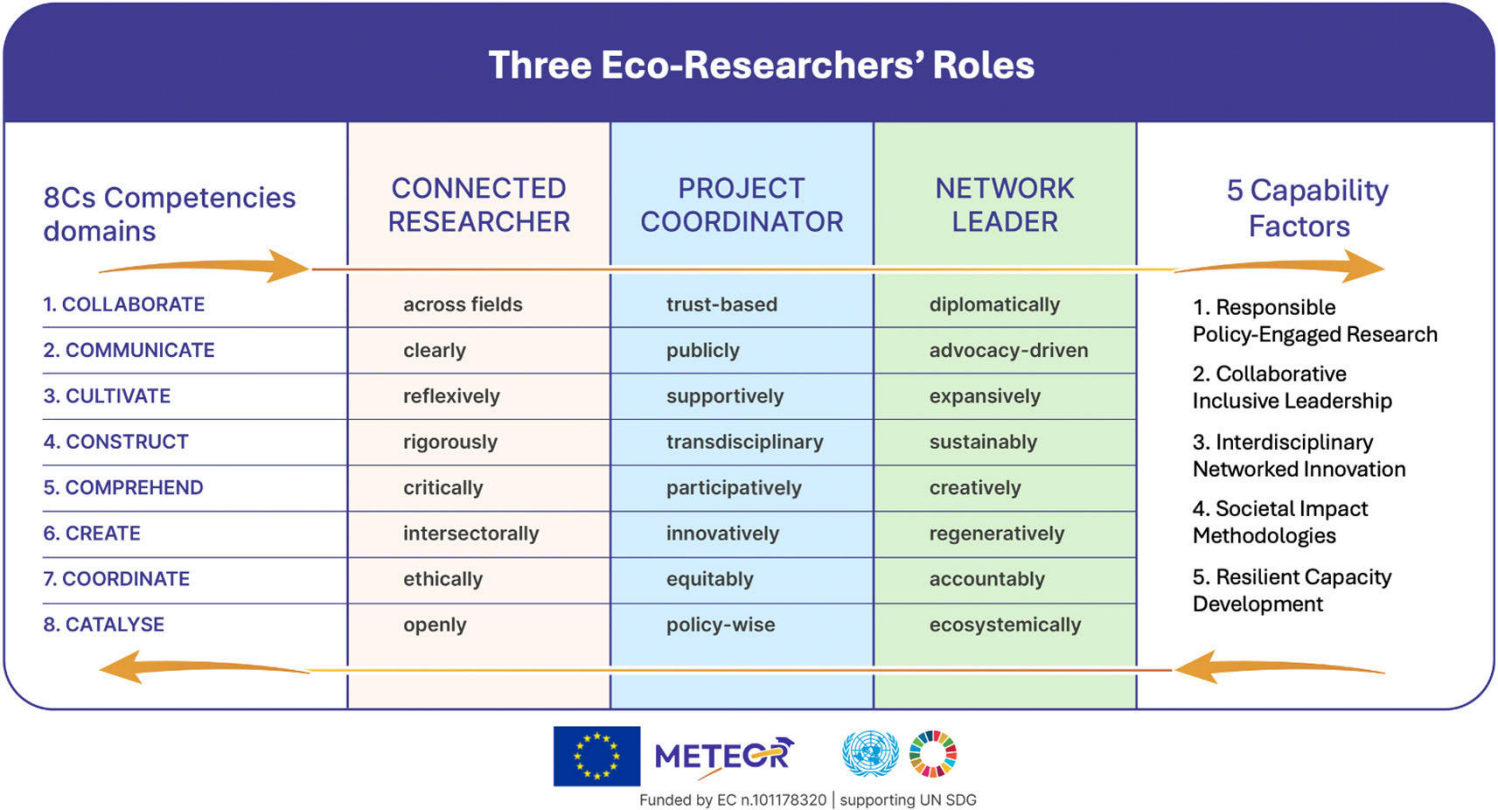
The 8Cs competency model mapped across three progressive eco-researcher roles — Connected Researcher, Project Coordinator, and Network Leader. Source: Okada, 2025a.

Figure 5 illustrates how eco-researcher identity develops progressively across three roles, with each 8Cs competency becoming more outward-facing, systemic, and regenerative in its mode of enactment — from individual disciplinary practice toward ecosystemic, policy-embedded action. Each competency is paired with its associated capability factor (F1–F5); for example, the COLLABORATE domain shifts from *across fields* (Connected Researcher), to *trust-based* (Project Coordinator), to *diplomatically* (Network Leader).

Conceptually and empirically, this figure provides a synthetic visual of the eco-outwards competence architecture across roles, linking competency-as-worldview to the 8Cs, CARE–KNOW–DO principles, and multiple roles enacted by individuals, teams and networks.

***5.1.1 Theoretical implications for regenerative research***

This is not a weakness of the instrument but a substantive finding: it suggests that researchers experience societal impact as simultaneously a methodological commitment and an ethical-policy responsibility, cutting across both factors rather than residing neatly in one. This cross-factor permeability is consistent with the CARE–KNOW–DO model, in which doing (methodology) and caring (ethical responsibility) are conceived as inseparable, and warrants examination in Phase-2 through confirmatory factor analysis of whether a bifactor or hierarchical structure better represents this overlap.

### 5.2 Key empirical insights

In this Phase-1 analysis, all five factors are widely valued by researchers, yet aspiration–practice gaps arise predominantly from institutional barriers (funding constraints, time poverty, siloed promotion, weak cross-sector partnerships)—not individual deficits ( Boxes 1– 5). These aspiration–practice gaps map onto the three eco-outwards roles in Figure 5, where institutional conditions shape how far researchers can move from connected practice to project and network leadership. This robustness emerges from Design-Based Research (DBR) capturing researchers as societal actors (citizens/professionals/future eco-outwards), whose lived experiences illuminate development trajectories. Three key patterns emerged:

Resilience is not purely individual: Participants articulated how personal resilience is strengthened by institutional support-mentoring relationships, wellbeing initiatives, and formal conflict-resolution structures. Their narratives suggest these systemic supports enable individual capacity-building rather than placing the burden solely on the individual.

Policy engagement is structured, not spontaneous: Researchers described policy-engaged work as requiring deliberate institutional conditions: co-creation relationships with stakeholders, sustained partnership frameworks, and formal recognition mechanisms within evaluation systems. Without these structures, policy engagement remains episodic rather than systematic.

Interdisciplinary collaboration requires systemic conditions: Participants identified three concrete enablers: dedicated time allocated to cross-faculty work, adequate resources for collaborative projects, and organizational cultures that legitimize interdisciplinary practice. In their absence, researchers revert to disciplinary silos despite strong motivation to collaborate.

Individual motivation alone is insufficient: This underscores that eco-outwards researchers need coordinated institutional, policy, and sectoral support to thrive ( Renick *et al*., 2024; Martínez & Tejero, 2025).

We conceptualise the eco-researcher identity drawing together three interrelated strands. First, beyond technical expertise, it foregrounds ethical–ecological responsibility, treating research as accountable to planetary boundaries and social justice rather than to narrow performance metrics. Second, it cultivates place-based and more-than-human awareness, recognising that knowledge is situated in specific communities, ecosystems and histories, and that research must respect human and non-human interdependence. Third, it embraces transdisciplinary engagement, working across disciplines and sectors with communities, policymakers and practitioners to co-define problems and co-produce solutions.

It embeds futures and systems thinking, linking present decisions to intergenerational impacts through anticipatory governance and futures literacy. Eco-researchers adopt an action-orientation beyond the academy, seeing publication as one step within wider cycles of collaboration, advocacy and institutional change. Finally, this orientation is grounded in regenerative lifelong learning, in which continual reflection, equity-oriented development and resilience are cultivated to restore and transform socio-ecological systems rather than merely minimise harm.

This study illuminates what eco-outwards research looks like in practice. An institutional response rate of less than 2%, contrasted with 230% growth driven by peer activation, is consistent with an aspiration-practice gap. Although the low response rate constrains population-level generalisation, three converging features lend analytical weight to the findings. Within narrative inquiry, a self-selected sample of practitioners already engaged with the phenomenon under study is often *more* epistemically valuable than a large random sample, precisely because participants can articulate tensions, barriers, and aspirations from inside lived experience. The persistent non-response despite repeated institutional reminders is itself a naturalistic signal about structural conditions surrounding researcher engagement — not merely a methodological inconvenience. Finally, the internal consistency between participants’ reported aspirations, perceived barriers, and observable practice patterns suggests these are genuine, ecologically valid responses rather than artefacts of instrument design or researcher expectation. Together, these three features justify treating the findings as analytically meaningful within their declared methodological frame, even while acknowledging the limits of their generalisability.

The study proposes five defining features of the eco-outwards researcher, framing this identity as a competency-as-worldview that must be enacted, not merely held:

• Ethical-ecological responsibility
• Place-based and more-than-human awareness
• Transdisciplinary engagement
• Futures and systems thinking
• Action-orientation beyond the academy

A synthesis of these strands-Eco-researchers’ Capability Domains, Defining Features, and Emerging Trends-presented in Table 9, integrates EFA, CARE-KNOW-DO, and professional identity developement in a way that extends beyond the empirical findings. This synthesis constitutes the paper’s primary theoretical contribution: a five-feature model of the eco-researcher grounded in empirical evidence.

**Table 9.** Eco-Researchers from Identity to Practice.

Eco-Outwards Defining Features (Emerging Trend)	Enabling Capability Domain	Enactment in Research Practice
Ethical–ecological responsibility ( **Regenerative Education**)	Responsible, policy-engaged research	Integrates ethical reasoning, reflexivity, and engagement with policy and public stakeholders to anticipate and address research impacts
Place-based and more-than-human awareness ( **Place-Based & Plural Epistemic Education**)	Collaborative, inclusive leadership	Cultivates attentive, co-creative relationships with diverse stakeholders, including global majority and less represented groups, and local environments, fostering recognition of multiple ways of knowing
Transdisciplinary engagement ( **Inter- & Transdisciplinary Innovation Learning**)	Interdisciplinary networking for innovation	Enables cross-sector and cross-discipline collaboration to co-design solutions for complex societal challenges, drawing on diverse knowledge systems and practitioner expertise beyond academia
Futures and systems thinking ( **Futures Literacy & Human-Planet Led AI to enhance Systems Thinking**)	Methodologies for social impact	Uses participatory, mixed, and creative approaches to understand systemic interconnections, anticipate long-term consequences, and leverage AI-informed insights
Action-orientation beyond the academy ( **Action Research & Societal Impact Learning**)	Resilient capacity development	Strengthens emotional resilience, adaptability, and supportive cultures, enabling sustained action and practical knowledge translation

This interpretation is consistent with the paper’s central argument that aspiration-practice gaps are institutional rather than individual in origin — here, even the measurement instrument risks reproducing the individualisation of resilience that the paper critiques.

These features are sustained through regenerative lifelong learning, a cross-cutting orientation that shapes the eco-researcher over time and connects individual reflection to systemic, intergenerational responsibility. They are analytically derived from engagement with five practice-oriented capability domains, which provide a practical language for reflection and development.

### 5.3 Policy and framework alignment

The sustainability and scaling of eco-outwards researcher development depend on coordinated alignment across funders, policymakers, employers, and professional networks. Despite strong international commitments, including the Sustainable Development Goals and Horizon Europe, structural barriers persist, such as short-term contracts, narrow evaluation metrics, and limited investment in researcher development.

Phase-1 identifies three leverage points for systemic change: funding and evaluation structures; recruitment and progression systems; and professional networks — each addressed in §5.4.

The upSKILL.map framework aligns with major international and national policy agendas, including the Sustainable Development Goals (SDGs 4, 10, and 16), Horizon Europe Strategic Plan 2025–2027, Responsible Research and Innovation (RRI), and the Global Sustainable Development Report (2023). These frameworks emphasise interdisciplinarity, societal engagement, and the integration of sustainability competencies across sectors. upSKILL.map complements the Vitae Researcher Development Framework by explicitly foregrounding ethics, justice, sustainability, and societal responsibility, supporting the advancement of researcher identity towards more inclusive, future-oriented roles.

In Phase-1, upSKILL.map functioned as a diagnostic and reflective instrument rather than an intervention, enabling researchers to articulate their developmental needs and eco-outwards perspectives. Within a self-selected institutional cohort, participants revealed shared commitments to sustainability and societal engagement, indicating the emergence of an eco-outwards research community. These findings highlight the potential of competency-based frameworks to support systemic transformation when embedded within aligned institutional and policy environments.

### 5.4 Phase-2 Research agenda: From diagnosis to systemic transformation

Importantly, this agenda explicitly reframes societal engagement not as a dissemination activity at the end of research, but as a practice-embedded, participatory process spanning research design, training, partnerships, and career development across sectors. Building on Phase-1 diagnostic insights, Phase-2 research advances three substantive questions that position this work within emerging fields:

**Curriculum and developmental pathways:** Multi-institutional studies should test whether embedding eco-outwards competencies in early researcher training, using CARE–KNOW–DO principles, correlates with sustained engagement in responsible and collaborative research across career stages—including non-academic pathways in government, industry, NGOs, and civil society.

**Competency evolution and career outcomes:** Phase-3 will extend Phase-1 and Phase-2 findings through longitudinal, multi-institutional research (target: 10+ institutions, n = 300–500 researchers tracked over 3+ years). This larger scale enables Confirmatory Factor Analysis (CFA) and concurrent mixed-methods inquiry to examine: (1) How do researchers’ five-factor eco-outwards profiles evolve over time? (2) How do these trajectories relate to career destinations within and beyond academia? (3) How does upSKILL.map use correlate with measurable increases in collaborative, responsible, and regenerative research practices? (4) How do institutional barriers and enablers (identified in Phase-1) constrain or facilitate eco-outwards competency development at different scales?

**Systemic enablement:** Phase-2 research should examine how aligning institutional promotion criteria, mentoring systems, and cross-sector partnerships with five-factor competencies affects research culture and researcher orientation. Evidence from early adopters—institutions embedding the framework into the Researcher Development Concordat—could illuminate barriers and enablers for scaling eco-researcher communities.

Together, these investigations position upSKILL.map not only as a reflective diagnostic tool but as a mechanism for systemic transformation, advancing doctoral education’s contribution to global sustainability and equity agendas. The following questions situate this research within wider emerging fields and debates, advancing how doctoral education and researcher development respond to polycrisis-era agendas across institutional, national, and international levels:

Futures literacy and anticipatory competence: Can upSKILL.map be integrated into futures literacy curricula to strengthen researchers’ capacity for systems thinking, temporal responsibility, and engagement with long-term sustainability scenarios? This will be examined in Phase-2 through embedded curriculum pilots at two external METEOR academies, with longitudinal tracking to assess competency change over time. Factor 3’s clustering of adaptive thinking, cross-disciplinary synthesis, and network-building offers the Phase-1 entry point for this enquiry.

### 5.5 Institutional pathways for transformation

The upSKILL.map instrument operationalises the 8Cs competency model across three interconnected levels: individual development (micro), programme and institutional structures (meso), and alignment with national and global frameworks (macro). This multi-level applicability enables sustainability, equity, and societal responsibility to be embedded as core components of researcher development rather than supplementary enhancements.

The five-factor structure provides a practical foundation for assessing researcher development needs, informing curriculum design, strengthening supervision practices, and aligning institutional strategy with global research and policy priorities, pending Phase-2 confirmation. This structured approach supports the eco-researcher and contributes to strengthening research capacity and global knowledge exchange.

Institutional transformation requires coordinated action across multiple stakeholder groups, including programme leaders, institutional leadership, funders, employers, and professional associations ( Table 10). Embedding eco-outwards competencies across curriculum, mentoring, recruitment, evaluation, and partnership systems enables interdisciplinary collaboration, inclusive leadership, and policy-engaged research.

**Table 10.** Fostering Eco-Outwards Identity Across Stakeholders (Multi-level Actions & Outcomes).

Stakeholder Group	Key Actions	Expected Outcomes
Programme Leaders & Curriculum Designers	Embed 8Cs model and CARE–KNOW–DO principles; integrate eco-outwards competencies into training and assessment	Researchers develop systems thinking, ethical responsibility, collaboration, public engagement
Research Project Coordinators	Foster peer mentoring, collaborative projects; use upSKILL.map to identify needs; recognize team outputs	Inclusive communication, epistemic justice, shared leadership
Research Network Leaders	Establish structured partnerships with government, NGOs, civil society; allocate resources for policy-informed activities	Stronger researcher capacity, sustained policy engagement
Organizational Heads & Directors	Promote cross-faculty groups, mentorship, inclusive cultures; revise promotion pathways	Interdisciplinary collaboration, well-being support, recognition of eco-outwards leadership
Funders & Policymakers	Prioritize researcher development funding; integrate five-factor assessment; support cross-institutional networks	Enable long-term, policy-engaged research
Employers & Industry	Align recruitment/evaluation with eco-outwards competencies; provide mentoring & cross-sector development	Strengthen transferable skills and leadership
Professional Associations & Networks	Explore five-factor framework; foster communities of practice; reward societal impact	Cultivate collaborative, sustainable, and policy-oriented research culture

These insights highlight the urgent need for strategies to bridge the gap between aspiration and practice by embedding policy and community engagement across all phases of research. Prioritising opportunities for students and early-career researchers to engage in real-world issues from the outset—through co-design, sustained collaboration, and iterative negotiation of research use—can help build the skills captured in Factor 1. Equally, institutional reform, including addressing funding limitations and rigid structures, is essential to cocreate an enabling environment where responsible, impactful research is systematically supported.

Overall, Factor 5 emerges as a cross-cutting capability that underpins the capacity to persist, adapt, and grow amidst adversity. Strengthening resilience requires moving beyond reliance on individual coping strategies to embed institutional supports—mentorship schemes, conflict resolution training, wellbeing initiatives, and career development pathways—that enable researchers to thrive and contribute to a sustainable, inclusive research environment.

***5.5.1 Enacting Eco-Outwards Orientation in Practice***

Evidence from the METEOR-UK initiative at The Open University suggests the practical feasibility of embedding eco-outwards competencies within diverse institutional contexts. The Phase-1 five-factor clustering has informed redesigned induction pathways, including academy, incubator, and accelerator opportunities. In particular, Responsible policy-engaged research and Collaborative inclusive leadership support structured induction and mentoring activities; Interdisciplinary networked innovation informs cross-faculty review and collaboration processes; and Resilient capacity development underpins supervisor development initiatives, positioning supervisors as mentors supporting eco-outwards researcher.

Within the Faculty of Science, Technology, Engineering and Mathematics (STEM), the framework aligns with researcher career development priorities, including early-career researcher support structures ( Mostéfaoui & Gardner, 2021). Its application strengthens programme innovation by supporting the advancement of transferable competencies relevant across academic, industry, educational, and international research contexts ( Open University, 2024a, 2025).

Within the Faculty of Wellbeing, Education and Language Studies (WELS), the framework supports strategic priorities focused on inclusive research cultures, meaningful career development pathways, and continuous institutional improvement ( Open University, 2018, 2024a, 2024b). It contributes to values-led researcher development and strengthens institutional capacity to respond to evolving societal, educational, and sustainability challenges. Complementary institutional initiatives, such as the Open Societal Challenges programme, further support postgraduate researchers in contributing to impact-oriented projects addressing sustainability, wellbeing, and social inequalities ( Open University, 2023; Dana *et al*., 2021).

Together, these institutional applications indicate how curriculum integration, structural alignment, stakeholder coordination, and career development pathways can operationalise eco-outwards researcher identity. Embedding justice, equity, and sustainability as core components of researcher development supports evidence-informed institutional transformation and strengthens the capacity of research systems to address complex global challenges ( Cole *et al*., 2023; McAlpine & Amundsen, 2018). The aspiration–practice gaps documented across all five factors — and the institutional conditions participants identified as barriers — provide the evidential basis for four practice-oriented recommendations.

***R1. Curriculum and assessment integration***

Phase-1 findings suggest that embedding the 8Cs model and five-factor structure within doctoral curriculum design, supervision, and programme evaluation strengthens structured competency development. The upSKILL.map instrument supports this by identifying competency gaps, enabling tailored capacity-building, and informing annual review processes aligned with societal engagement and sustainability priorities.

***R2. Structural and cultural transformation***

Phase-1 evidence suggests that promotion and reward systems that recognise collaborative contributions, inclusive leadership, and policy-engaged research are associated with stronger eco-outwards competency development. Institutional cultures that support eco-outwards research mentoring, interdisciplinary collaboration, and equitable participation enable the advancement of eco-researcher identities ( Martínez & Tejero, 2025; Renick *et al*., 2024; Lawrence *et al*., 2024; Horcea-Milcu *et al*., 2024).

***R3. Stakeholder alignment and coordinated investment***

Phase-1 findings point to coordinated engagement across stakeholder groups, including programme leaders, institutional leadership, funders, employers, and professional networks as a priority condition for sustainable transformation. Strengthening institutional support for mentoring, researcher development, and cross-sector collaboration is appears to be an important enabler of sustained societal engagement and advancing eco-outwards research practice.

***R4. Career pathway diversification and employability***

Phase-1 competency profiles suggest doctoral education is strengthened when it prepares researchers for diverse career pathways across academia, policy, industry, and civil society. Developing transferable competencies—including systems thinking, collaborative leadership, and stakeholder engagement—enhances employability and supports sustainable and responsible innovation ( European Commission, 2023).

## 6. Conclusion

The claims below are theoretical syntheses informed by Phase-1 evidence; empirical generalisations await Phase-2 confirmation.

This study argues that sustainability, equity, and justice can be treated as integral to the eco-researcher, shaping professional practice rather than serving as supplementary considerations. Researchers’ engagement with SDG-aligned impact mechanisms and qualitative narratives illustrates that these principles function as foundational commitments guiding ethical, socially responsive, and regenerative research. The five preliminary capability factors—Responsible policy-engaged research, Collaborative inclusive leadership, Interdisciplinary networked innovation, Societal Impact Methodologies, and Resilient Capacity Development—capture the coherent structure of eco-outwards competencies, highlighting the interconnected nature of research capabilities that extend beyond technical expertise. Qualitative evidence further reveals an aspiration–practice gap, in which researchers value these commitments but encounter institutional and systemic barriers, positioning the eco-outwards research framework as a conceptually and empirically grounded orientation with real-world implications.

While the eco-researcher emerged from a self-selected cohort, it represents one legitimate professional orientation among diverse research pathways, including fundamental, applied, and curiosity-driven inquiry. By demonstrating that competency functions as a worldview, this study challenges conventional frameworks that treat ethics, sustainability, and societal engagement as optional extensions of technical skill ( Owen *et al*., 2022; Cole *et al*., 2023). This perspective addresses longstanding critiques of linear knowledge translation and the ‘valley of death’ between discovery and implementation ( Lenfant, 2003; Butler, 2008; Greenhalgh & Wieringa, 2011), as well as narrow, thesis-focused doctoral apprenticeship models that often leave graduates underprepared for interdisciplinary, policy-engaged, and societal roles ( Spronken-Smith, 2018). The eco-researcher emerges as a professional orientation capable of navigating polycrises in regenerative education, green economies, nature-based solutions, and equitable digital transformation, while remaining aligned with broader academic and disciplinary goals.

The upSKILL.map instrument operationalizes this framework as a bridge between individual capability development and systemic transformation. Beyond technical skill acquisition, it supports problem definition, co-production of knowledge, and evaluation of societal and sustainability impacts. By surfacing gaps across competency domains, upSKILL.map allows training centers, academic leaders, and mentors to redesign pathways that cultivate eco-outwards capabilities and close research–practice gaps ( Taylor & Archer, 2022). Its structured mechanisms, including portfolios/digital badges ( Abdelghaffar & Eid, 2025), provide transparent, transferable evidence for career progression, formative feedback, and institutional benchmarking.

This study contributes to global efforts to reposition sustainability, equity, and justice as core dimensions of researcher development ( Cole *et al*., 2023; Lähteenmäki-Uutela *et al*., 2024), in alignment with the United Nations Sustainable Development Goals ( United Nations, 2023), Horizon Europe’s Responsible Research and Innovation agenda ( European Commission, 2023, 2025a, 2025b), and emerging models of transformative and regenerative research systems ( Horcea-Milcu *et al*., 2024; Dedeurwaerdere, 2024). The eco-researcher identity exemplifies orientations already embodied by scholars in polycrisis contexts. This orientation aligns with higher education imperatives to cultivate ethical imagination, civic responsibility, and collaborative problem-solving capacities necessary for addressing complex global challenges ( Reimers, 2020; UNESCO, 2020). It further reflects regenerative learning paradigms that restore socio-ecological systems, foster integrative and ethical forms of intelligence, and enable understanding-oriented knowledge transfer across real-world contexts ( Sterling, 2021; van den Berg & Wals, 2022; Gardner, 2011; Perkins, 2010). Phase-2 and Phase-3 will extend this work through multi-institutional replication, longitudinal tracking, and confirmatory factor analysis to test generalisability beyond the present cohort.

By articulating and preliminarily characterising eco-outwards capabilities within a self-selected Open University cohort, this study offers an analytically transferable framework that, with multi-institutional adaptation, can inform research culture across higher education. The eco-outwards research framework, enacted through the upSKILL.map instrument, links individual development to systemic transformation. In doing so, the study advances a shift from competency as individual performance toward capability as a worldview — fostering relational, responsible, and future-oriented research practices that align research systems with planetary stewardship and the common good.

## Ethics declarations

The study was conducted in accordance with the Declaration of Helsinki and approved by The Open University Human Research Ethics Committee (HREC 2025–0889-4).

## Informed consent statement

Informed consent was obtained from all participants involved in the study (written form).

## AI use disclosure

This study selectively employed AI technologies to support research and writing. **Perplexity** assisted with APA formatting and language consistency, while **ChatGPT** helped condense the abstract to 300 words. A UML diagram was generated using an AI image tool, and **Springer AJE ProofReader** provided additional language editing. **NVivo** and **ATLAS AI** supported verification of thematic analyses. Quantitative analyses were conducted using **SPSS** and **R**, with **Claude** providing coding assistance. All results were carefully reviewed to ensure accuracy and integrity. The full manuscript underwent review by four internal team members and two external partners.

## Data Availability

The data are available via **Open Research Data Online (ORDO)** at The Open University. All datasets were prepared according to the project’s data management plan and reviewed by the Open Research Library Team. The database captures responses from 40 researchers who participated in the **upSKILL** study.
•
**Project Repository:** METEOR Open Repository Online Data. The Open University.
https://ordo.open.ac.uk/projects/METEOR/244817
•
**Dataset:** upSKILL.map Phase-1 dataset. The Open University.
https://doi.org/10.21954/ou.rd.30108928
•
**Instrument:** upSKILL.map self-reported instrument [Questionnaire]. The Open University.
https://doi.org/10.21954/ou.rd.30109057
•
**Qualitative Analysis:** Thematic Codebook.
https://doi.org/10.21954/ou.rd.31557559
•
**Quantitative Analysis:** Appendix 1 – upSKILL.map data.
https://doi.org/10.21954/ou.rd.31558528
•
**Video Abstract:** Overview of the eco-outwards research framework, upSKILL.map.
https://www.youtube.com/watch?v=Cl91GhlrHmU All materials are available under a Creative Commons Attribution–ShareAlike (CC BY-SA) licence. Data transparency commitments have been balanced against participant protection obligations within the bounds of participants’ informed consent, particularly given the identifiability risk inherent in a small sample with intersecting demographic characteristics — a tension increasingly recognised in open science guidance for qualitative and mixed-methods research ( Weller, 2023; Barnes & Weller, 2017; Van den Eynden *et al*., 2011). In keeping with the participatory ethics underpinning this study, participants were offered collective attribution in published outputs including the think piece and video clip — recognising their role as co-producers of knowledge and enacting the data sovereignty commitments that governed all data collection, sharing, and retention decisions in this study.
