# Supplementary material for: Developing Researchers’ Competencies through CARE–KNOW–DO and upSKILL.map, aligned with EU and UNESCO Priorities
Source: Open Res Eur. 2026 Jul 16;5:333. Originally published 2025 Oct 31. [Version 4] doi: 10.12688/openreseurope.21408.4 (PMC13198694; doi:10.12688/openreseurope.21408.4)
Supplement: Peer review history

# Reviewer report: Reviewer response for version 4

**DOI:** 10.21956/openreseurope.26620.r77494

**Published:** 2026-08-06

**Authors:** Moses Adeleke ADEOYE

**Affiliations:** 1 Al-Hikmah University, Ilorin, Nigeria

**Approved.**

If applicable, is the statistical analysis and its interpretation appropriate?

Partly

Is the study design appropriate and is the work technically sound?

Partly

Is the work clearly and accurately presented and does it engage with the current literature?

Partly

Are the conclusions drawn adequately supported by the results?

Partly

Are sufficient details of methods and analysis provided to allow replication by others?

Partly

Are all the source data and materials underlying the results available?

Partly

Reviewer Expertise:

NA

I confirm that I have read this submission and believe that I have an appropriate level of expertise to confirm that it is of an acceptable scientific standard.

# Reviewer report: Reviewer response for version 4

**DOI:** 10.21956/openreseurope.26620.r77496

**Published:** 2026-07-20

**Authors:** Amelie E Trinidad

**Affiliations:** 1 Davao del Sur State College, Matti, Digos City, Davao del Sur, Philippines

I have no further comments on this revised manuscript. The authors have adequately addressed the concerns and suggestions raised in my previous review, and the revisions have improved the clarity and overall quality of the manuscript. I appreciate the authors' careful consideration of the review comments and have no additional recommendations at this stage.

If applicable, is the statistical analysis and its interpretation appropriate?

Partly

Is the study design appropriate and is the work technically sound?

Partly

Is the work clearly and accurately presented and does it engage with the current literature?

Partly

Are the conclusions drawn adequately supported by the results?

Partly

Are sufficient details of methods and analysis provided to allow replication by others?

Yes

Are all the source data and materials underlying the results available?

Partly

Reviewer Expertise:

Educational Management; Higher Education; Doctoral Education; Teacher Education; Research Methodology (Qualitative, Quantitative, and Mixed Methods); Research Competency Development; Faculty and Research Capacity Building; Curriculum Development; Educational Leadership; Program Evaluation; Quality Assurance and Accreditation; Artificial Intelligence in Education.

I confirm that I have read this submission and believe that I have an appropriate level of expertise to confirm that it is of an acceptable scientific standard.

# Reviewer report: Reviewer response for version 3

**DOI:** 10.21956/openreseurope.25732.r75182

**Published:** 2026-06-11

**Authors:** Mona Holmqvist

**Affiliations:** 1 Lund University, Malmö, Skåne County, Sweden

**Manuscript Title**: Developing Researchers'’ Competences through CARE-KNOW-DO and upSKILL.map, aligned with EU and UNESCO Priorities

**Overall recommendations**: Approved with minor revisions

 The revised version of the article has been improved, but the comments I had in my previous review have been addressed only partly. At pages 7 and 9, the authors refer to the “aspiration-practice gap”, and at p. 15, they refer to “advance participatory research principles” referring to the participants in the conducted study. However, my comments in the previous review addressed the question of “who defines the challenges” studied by the researchers. If PhD students and supervisors are supposed to advance participatory research principles, the tool should map the competencies developed for engaging members in the contexts where data are collected. The competence to develop skills that involve respondents in all phases of the research process, from design to dissemination, should be captured. The tool examines PhD students' and supervisors' self-assessed views on collaboration, focusing primarily on collaboration between researchers rather than their competences to collaborate with respondents involved in the research, which is not problematized or discussed.  Relational competences are needed for collaborative work with the respondents participating in the PhD students and their supervisors’ research. Several theoretical frameworks have contributed to the upSkill.map, however, perspectives such as improvement science and collaborative practice-oriented research are not mentioned, although they could be used to understand what competences future researchers need to conduct participatory research with high societal impact.

**Is the work clearly and accurately presented and does it engage with the current literature?**

 Yes

**Is the study design appropriate and is the work technically sound?**

 Yes

**Are sufficient details of methods and analysis provided to allow replication by others?**

 Yes

**Are all the source data and materials underlying the results available?**

 Yes

**If applicable, is the statistical analysis and its interpretation appropriate?**

 I do not have the competence to review this part, however the sample of 40 participants are small.

**Are the conclusions drawn adequately supported by the results?**

 Yes

If applicable, is the statistical analysis and its interpretation appropriate?

I cannot comment. A qualified statistician is required.

Is the study design appropriate and is the work technically sound?

Yes

Is the work clearly and accurately presented and does it engage with the current literature?

Yes

Are the conclusions drawn adequately supported by the results?

Yes

Are sufficient details of methods and analysis provided to allow replication by others?

Yes

Are all the source data and materials underlying the results available?

Yes

Reviewer Expertise:

As a Professor of Educational Science my research focuses on the intersection of learning, teaching, inclusion, and educational improvement. A central feature of my work is the development and study of practice-based research conducted in close collaboration with teachers, schools, and educational organizations. My research is characterized by long-term partnerships with practitioners and a commitment to generating knowledge that contributes simultaneously to educational theory and the improvement of professional practice. I have conducted research on doctoral education and supervision, focusing how doctoral candidates develop as researchers, how supervisory relationships evolve during different phases of doctoral studies, and which forms of supervision are perceived as most valuable by doctoral students.

I confirm that I have read this submission and believe that I have an appropriate level of expertise to confirm that it is of an acceptable scientific standard.

## References

[1] : Research–Practice Partnerships in Education. *Educational Researcher* .2016;45(1) : 10.3102/0013189X1663175048-54 10.3102/0013189X16631750

[2] : EdD Students’ Sensemaking of Improvement Science as a Tool for Change in Education. *Journal of Research on Leadership Education* .2025;20(1) : 10.1177/194277512312210323-31 10.1177/19427751231221032

# Response

**Published:** 2026-06-11

**Authors:** Alexandra Okada

**Affiliations:** Education, The Open University Faculty of Wellbeing Education and Language Studies, Milton Keynes, England, UK

We thank Dr Holmqvist for her continued, constructive engagement with the manuscript and are pleased that she now recommends approval, with all evaluation criteria assessed as "Yes." We respond to her two remaining points below.

**Who defines the challenges, and participatory engagement with respondents.**

 This concern is taken up directly in Section 2.3 and Section 2.4. Part-1 of upSKILL.map asks participants to reflect on who contributes to the framing of research priorities, embedding reflexivity on power and agenda-setting from the outset rather than treating challenges as pre-defined problems to be "solved."

 Section 2.4 ("Embedding power and agenda-setting reflexivity") makes this explicit: participants describe how research issues are co-defined through engagement with communities, funders, policy contexts, and practitioners, and consider who influences what these issues become.

 Relational and participatory engagement with the contexts in which data are generated is also represented in the competency structure — in the Construct domain (evidence-based, participatory solutions), the Catalyse domain (community empowerment, and open research for equity and inclusion, items 8.1 and 8.3), and most directly in Factor 4 (Societal Impact Methodologies), where the qualitative evidence (Box 4) foregrounds participant-led methods and working with communities and stakeholders across the research lifecycle rather than informing them only at later stages.

 We agree that the measurement of relational competence — the specific capacity to involve respondents and community partners across all phases, from design to dissemination — is currently captured at the level of perceived relevance rather than as a discrete, behaviourally defined competence. As set out in Section 3.4 and Section 5.4, refining the instrument to capture this more granularly through behaviourally anchored items is a defined Phase-2 development priority. We have therefore framed this as an acknowledged direction for instrument development.

**Improvement science and collaborative practice-oriented research.**

 We are grateful for these suggestions and for the Coburn and Penuel (2016) and Bonney et al. (2025) references. We agree they offer a valuable lens on the competences required for participatory, high-impact research, and that they complement the participatory and co-design commitments already grounded in the manuscript through Responsible Research and Innovation (Stilgoe et al., 2013; von Schomberg, 2013), epistemic-justice and capabilities perspectives (Walker & Boni, 2006), and narrative inquiry (Clandinin & Connelly, 2000). Research–practice partnerships and improvement science are a natural theoretical extension for the Phase-2 work, in which relational and participatory competences will be operationalised, and we will draw on this literature as that phase develops.

 We thank the reviewer again for a careful and generative review.

# Reviewer report: Reviewer response for version 2

**DOI:** 10.21956/openreseurope.25047.r72186

**Published:** 2026-05-23

**Authors:** Carlos Enrique GEORGE-REYES

**Affiliations:** 1 Bolivarian University of Ecuador, Duran, Guayas, Ecuador

**Brief description and relevance of the article**

 The manuscript presents the development and Phase 1 contextual validation of upSKILL.map, a diagnostic and developmental self-assessment instrument designed to support researchers’ competencies through the CARE-KNOW-DO framework. The article is aligned with EU and UNESCO priorities and seeks to move beyond traditional competency frameworks focused mainly on employability or career management by foregrounding sustainability, equity, social responsibility, and researcher identity.

 The article is relevant to researcher development, doctoral education, responsible research and innovation, and sustainability-oriented higher education. Its main contribution lies in proposing a value-anchored model of researcher competencies and in offering preliminary empirical evidence for the coherence of the 8Cs framework and its five emerging capability factors. The manuscript is timely because institutions increasingly need tools to help researchers connect disciplinary expertise with societal impact, ethical responsibility, and global challenges.

**Overall assessment**

 Overall, I consider this a valuable and well-motivated manuscript. The article is conceptually ambitious, clearly connected to current debates on researcher development, and useful for institutions interested in strengthening doctoral training and early-career researcher support. The manuscript is generally well structured, the research problem is clearly presented, and the combination of quantitative and qualitative evidence provides a coherent basis for the authors’ argument.

 The article is suitable for publication. However, I recommend minor revisions to further improve readability, methodological precision, and consistency between the scope of the evidence and the claims made in the conclusions.

**Major comments**

1. **Clarify the scope and limits of generalisability**

The manuscript appropriately acknowledges that this is a Phase 1 study based on a single institution and a modest, self-selected sample. This limitation is important and should remain visible throughout the manuscript, particularly in the abstract, discussion, and conclusion. Some statements about institutional or global applicability could be further moderated to avoid implying that the model has already been validated beyond the specific context studied. The authors may consider consistently using terms such as “preliminary evidence,” “context-bound validation,” or “exploratory findings” when discussing the factor structure and the potential institutional uses of upSKILL.map.

1. **Reduce conceptual density and repetition**

The theoretical framing is one of the strengths of the manuscript, especially the distinction between competency-as-performance and competency-as-worldview. Nevertheless, some sections of the introduction and conceptual framework are dense and occasionally repetitive, particularly when explaining sustainability, equity, intergenerational responsibility, and the eco-researcher identity. I recommend streamlining selected paragraphs to improve readability while preserving the conceptual depth of the argument. This would help readers follow the progression from existing frameworks to the specific contribution of upSKILL.map more easily.

1. **Strengthen the presentation of the methodological sequence**

The use of design-based research, exploratory factor analysis, qualitative think-pieces, expert review, and user feedback is appropriate for a Phase 1 study. The manuscript provides useful methodological details, but the overall sequence could be made even clearer for readers. I suggest adding or refining a concise methodological flow that shows how the initial 32 items were developed, how four items were removed, how the final 28-item structure was analysed, and how the qualitative findings supported or refined the interpretation of the five factors. This would strengthen the transparency and replicability of the study.

1. **Maintain caution in the interpretation of the exploratory factor analysis**

The statistical analysis is generally well reported, and the authors provide justification for conducting exploratory factor analysis with a small sample. However, because the participant-to-item ratio remains limited, the factor structure should be presented explicitly as exploratory and provisional. The manuscript would benefit from a brief statement in the findings section clarifying that confirmatory factor analysis, measurement invariance testing, and larger multi-institutional samples are needed before the instrument can be used for broader validation or high-stakes decision-making.

1. **Deepen the integration between quantitative and qualitative findings**

The mixed-methods design is a valuable aspect of the study. To strengthen the integration of evidence, the authors could include a short joint display or summary table linking the five capability factors with selected qualitative themes from the think-pieces and interviews. This would help readers understand how the numerical factor structure aligns with participants’ reflections on institutional support, policy engagement, collaboration, leadership, and societal impact. Such a table would also make the practical implications for doctoral schools and researcher development units more explicit.

1. **Specify practical implications for implementation**

The manuscript argues that upSKILL.map can support doctoral schools and researcher development leads in identifying gaps and designing targeted interventions. This is a promising contribution. I recommend adding more practical guidance on how the tool should be used responsibly. For example, the authors could clarify whether it is intended for formative self-reflection, programme-level diagnosis, mentoring conversations, curriculum design, or institutional planning. It would also be useful to explicitly state that the tool should not be used as an individual performance evaluation instrument at this stage.

**Minor comments**

1. Please ensure consistency in the spelling and formatting of the instrument name throughout the manuscript, for example, upSKILL.map versus upSkill.Map.
2. Some terms should be standardised across the manuscript, including “eco-outwards,” “eco-researcher,” “8Cs,” “five capability factors,” and “competency-as-worldview.”
3. Several long paragraphs could be shortened to improve readability, especially in the introduction and theoretical framework.
4. Tables and figures are useful, but some visual elements could be improved for clarity and accessibility. Please ensure that all labels are readable and that each figure clearly supports the surrounding argument.
5. The manuscript would benefit from a final copy-editing pass to correct minor typographical or formatting issues, such as spacing, punctuation, duplicated periods, and occasional inconsistencies in capitalization.
6. Please review all citations and references for consistency, especially recent framework documents and institutional sources, to ensure that every in-text citation is included in the reference list and formatted consistently.
7. The discussion could more clearly distinguish between findings directly supported by the Phase 1 data and recommendations for Phases 2 and 3.

**Final recommendation**

 I recommend approval of the manuscript, subject to minor revisions. The article makes a meaningful contribution to the field of researcher development by proposing a values-based competency framework and a practical diagnostic tool aligned with sustainability, equity, and responsible research priorities. With modest revisions to improve clarity, methodological caution, and editorial consistency, the manuscript will offer a strong contribution to discussions on doctoral education, eco-social responsibility, and the future of research careers.

If applicable, is the statistical analysis and its interpretation appropriate?

Yes

Is the study design appropriate and is the work technically sound?

Yes

Is the work clearly and accurately presented and does it engage with the current literature?

Yes

Are the conclusions drawn adequately supported by the results?

Yes

Are sufficient details of methods and analysis provided to allow replication by others?

Partly

Are all the source data and materials underlying the results available?

Yes

Reviewer Expertise:

As a researcher, my work focuses on educational innovation, emerging digital literacies, artificial intelligence literacy, computational thinking, the educational impact of emerging technologies, teacher professional development, and disruptive pedagogical approaches such as Maker pedagogy. These research areas allow me to assess the manuscript from a perspective grounded in current debates on technology, education, and professional learning.

I confirm that I have read this submission and believe that I have an appropriate level of expertise to confirm that it is of an acceptable scientific standard.

# Response

**Published:** 2026-05-26

**Authors:** Alexandra Okada

**Affiliations:** Education, The Open University Faculty of Wellbeing Education and Language Studies, Milton Keynes, England, UK

Dear Carlos,

 Thank you for the constructive and thoughtful feedback provided on our manuscript. We have carefully reviewed all comments and implemented the recommended revisions to strengthen the clarity, coherence, and consistency of the paper. The following changes have been addressed:

**Instrument-name consistency**

 We reviewed the manuscript thoroughly and standardized the spelling and formatting of the instrument name throughout the text. All instances now consistently use the same format.

**Term standardization**

 Key terms have been standardized across the manuscript to ensure conceptual consistency and readability. This includes terms such as “eco-outwards,” “eco-researcher,” “8Cs,” “five capability factors,” and “competency-as-worldview.”

**Long paragraphs**

 Some lengthy paragraphs, particularly within the Introduction and Theoretical Framework sections, were revised and divided into shorter paragraphs to improve readability, structure, and flow of ideas.

**Tables and figures clarity**

 Tables and figures were reviewed to improve clarity and accessibility. Labels and formatting were refined to enhance readability, and visual elements were checked to ensure each figure directly supports the surrounding discussion and argumentation.

**Final copy-editing pass**

 A full copy-editing review was conducted to correct minor typographical and formatting issues, including spacing, punctuation, duplicated periods, tables' and figures'  links as well inconsistencies in capitalization.

**Citations and references**

 All citations and references were reviewed for consistency and completeness. Recent framework documents and institutional sources were checked carefully to ensure alignment between in-text citations and the reference list, with formatting standardized throughout.

**Phase 1 versus Phases 2 and 3 boundary in the Discussion**

 The Discussion section was revised to provide a clearer distinction between findings directly supported by Phase 1 data and the forward-looking recommendations proposed for Phases 2 and 3.

 We appreciate the valuable feedback, which has significantly contributed to improving the quality and clarity of the manuscript.

# Reviewer report: Reviewer response for version 2

**DOI:** 10.21956/openreseurope.25047.r72107

**Published:** 2026-04-21

**Authors:** Amelie E Trinidad

**Affiliations:** 1 Davao del Sur State College, Matti, Digos City, Davao del Sur, Philippines

**Review Report**

**Manuscript Title:** Developing Researchers’ Competencies through CARE–KNOW–DO and upSkill.Map, aligned with EU and UNESCO Priorities

**Authors:** Okada A., Sheehy K., Dieter Rossade K., & Bandara A.K.

**Overall Recommendation:** **Approved with Minor Revisions**

**A. Clarity and Engagement with Literature - Yes**

 The manuscript has significantly improved in terms of clarity and engagement with the literature. The introduction is now more concise and better structured, and the inclusion of a comparative table effectively clarifies how upSkill.Map advances existing competency frameworks (e.g., ResearchComp, Vitae RDF, OECD frameworks). This emphasis on structured competency development aligns with systematic evidence regarding the training of research skills in modern learning environments (Garay-Argandoña et al., 2021).. The integration of additional literature on research–practice gaps further strengthens the conceptual grounding of the study.

 However, some sections remain conceptually dense, with minor repetition in emphasizing sustainability, equity, and “competency-as-worldview.”

**Suggested Revision:**

 Further streamline selected sections of the introduction to improve readability and reduce conceptual redundancy while maintaining the strength of the argument.

**B. Study Design and Technical Soundness - Yes**

 The authors have adequately addressed previous concerns regarding the study design. The justification for conducting EFA with a small sample size (n = 40) is now well-supported by relevant literature, and the inclusion of parallel analysis using Monte Carlo simulations strengthens the robustness of factor retention decisions. The DBR process has also been clearly elaborated, with detailed descriptions of iterative cycles and decision points.

**Suggested Revision:**

 Reinforce within the findings section that the factor structure remains exploratory and context-bound to ensure cautious interpretation of results.

**C. Replicability of Methods - Yes**

 This aspect has been substantially improved. The manuscript now provides the full set of instrument items, clearly identifies removed items along with statistical criteria, and offers a transparent and detailed description of the qualitative analysis process, including coding procedures, dual coding, and the audit trail. The justification for not using intercoder reliability metrics is also appropriately grounded in methodological literature.

**Suggested Revision:**

 Consider presenting the qualitative coding process in a concise table or schematic diagram to enhance clarity and accessibility.

**D. Availability of Source Data - Yes**

 The authors have maintained strong compliance with open science practices by providing datasets, instruments, and supplementary materials through ORDO. The clarification regarding ethical considerations and qualitative data availability is appropriate and enhances transparency.

**E. Statistical Analysis and Interpretation - Yes**

 The statistical analysis is now robust and well-reported. The manuscript includes complete psychometric details such as factor loadings, communalities, eigenvalues, variance explained, KMO, Bartlett’s test, and Cronbach’s alpha. The addition of parallel analysis further strengthens the validity of the factor structure.

**Suggested Revision:**

 Slightly streamline the presentation of statistical results to reduce repetition between tables and narrative explanations.

**F. Support of Conclusions by Results - Yes**

 The conclusions are now more appropriately aligned with the results and are clearly framed as preliminary, context-bound, and part of a multi-phase research program. The distinction between empirical findings and forward-looking recommendations has improved.

**Suggested Revision:**

 Further moderate a few statements implying broader institutional or global applicability to ensure consistency with the study’s scope and sample limitations.

**Additional Suggestions**

 (Optional but beneficial to the manuscript)

- The inclusion of the schematic model and implementation guidance is commendable; minor refinement for clarity and usability may further enhance practical application.
- Continue improving manuscript flow through minor reductions in dense sections and consistent use of clear subheadings.

**Overall Recommendation: Approve with Minor Revisions**

 The revised manuscript demonstrates substantial improvement and successfully addresses the major concerns raised in the first review. It now presents a methodologically sound, conceptually coherent, and practically relevant contribution to researcher development literature. The remaining issues are minor and relate primarily to clarity, conciseness, and cautious interpretation.

**Evaluation**

**Is the work clearly and accurately presented and does it engage with the current literature?**

 Yes

**Is the study design appropriate and is the work technically sound?**

 Yes

**Are sufficient details of methods and analysis provided to allow replication by others?**

 Yes

**Are all the source data and materials underlying the results available?**

 Yes

**If applicable, is the statistical analysis and its interpretation appropriate?**

 Yes

**Are the conclusions drawn adequately supported by the results?**

 Yes

If applicable, is the statistical analysis and its interpretation appropriate?

Partly

Is the study design appropriate and is the work technically sound?

Partly

Is the work clearly and accurately presented and does it engage with the current literature?

Partly

Are the conclusions drawn adequately supported by the results?

Partly

Are sufficient details of methods and analysis provided to allow replication by others?

Yes

Are all the source data and materials underlying the results available?

Partly

Reviewer Expertise:

My areas of expertise include educational research, researcher development, competency frameworks, mixed-methods research, and instrument development/validation. I am qualified to assess the conceptual framework, study design, statistical analysis (including factor analysis), qualitative methods, and overall methodological rigor. I am not an expert in domain-specific content outside the scope of researcher competencies (e.g., specific EU or UNESCO policy implementation), so my review focuses primarily on methodological and analytical aspects.

I confirm that I have read this submission and believe that I have an appropriate level of expertise to confirm that it is of an acceptable scientific standard.

## References

[1] : Research competences in university students in virtual learning environments. *Cypriot Journal of Educational Sciences* .2021;16(4) : 10.18844/cjes.v16i4.60311721-1736 10.18844/cjes.v16i4.6031

# Response

**Published:** 2026-04-28

**Authors:** Alexandra Okada

**Affiliations:** Education, The Open University Faculty of Wellbeing Education and Language Studies, Milton Keynes, England, UK

We are grateful to the reviewer for the rigorous and constructive feedback. 

 Length and repetition. sections  1.1–1.2 have been tightened and overlapping passages in sections  3–5 consolidated. “Identity” usage has dropped from 58 to 20 occurrences; redundant summary paragraphs have been removed.

 Framework deficiency. section 1.2 now acknowledges that the Vitae RDF, ResearchComp, and OECD Core Framework address purposes distinct from those of the eco-outwards framework. upSKILL.map is positioned as serving researchers engaged with societal, policy, or sustainability challenges — a constituency currently underserved by existing frameworks — not as a universal model. Disciplinary excellence, curiosity-driven inquiry, and fundamental science are explicitly recognised as equally valid research identities.

 Sample size and psychometric claims. section 3.4 and section 4.1.3 state that n = 40 is below conventional thresholds and that the factor structure is descriptive and tentative, generating hypotheses for Phase-2 confirmatory testing (target n > 200, multi-institutional, pre-registered).

 EFA/PCA distinction. EFA is retained as the methodological framing. PCA with Varimax rotation is specified as the extraction method appropriate for descriptive dimension reduction at this pilot stage (Tabachnick & Fidell, 2013; Costello & Osborne, 2005). Principal Axis Factoring (PAF) with Oblimin rotation has been added as a common-factor robustness check, reported in section 4.1.3: four of five components replicate stably; one (Collaborative Inclusive Leadership) shows method-dependence under oblique rotation. Common-factor EFA is reserved for Phase-2.

 Relevance versus competence. We accept this distinction. Clarifying sentences in section 2.3 and section 3.4 state that Part-2 captures perceived relevance, not demonstrated competence. Phase-2 will pilot behaviourally-anchored items.

 Self-selection. Within narrative inquiry, a self-selected sample of practitioners already engaged with the phenomenon is often more epistemically valuable than a large random sample (Clandinin & Connelly, 2000). Section 5.2 acknowledges the limits on generalisation while identifying three converging features that lend analytical weight to the findings.

 Exploratory framing. The Abstract Results sentence now reads “converge on a five-factor descriptive clustering… as a baseline,” and its closing sentence specifies “further multi-institutional confirmation.” section 6 opens with “The claims below are theoretical syntheses informed by Phase-1 evidence; empirical generalisations await Phase-2 confirmation,” describes the five factors as “preliminary,” and closes with “offers a testable framework that, with multi-institutional replication, could inform research culture.”

 Restricted claims and propositional recommendations. Sector-wide statements across sections  5–6 are qualified to the Phase-1 cohort. R1–R4 in section 5.5 now open with “Phase-1 findings suggest…,” “Phase-1 evidence suggests…,” “Phase-1 findings point to…,” and “Phase-1 competency profiles suggest…” — testable propositions rather than declarative claims.

 Open science. All repository DOIs have been re-verified in the Data Availability section. SPSS syntax will be deposited prior to publication.

 Theory–evidence separation. Theoretical arguments are flagged with “we argue” or “we propose”; empirical demonstrations are bounded to the Phase-1 cohort; hypothetical claims for Phase-2 testing are explicitly marked in section 5.4 and R1–R4.

 We thank the reviewer for this careful and generous review

# Reviewer report: Reviewer response for version 2

**DOI:** 10.21956/openreseurope.25047.r72184

**Published:** 2026-04-17

**Authors:** Moses Adeleke ADEOYE

**Affiliations:** 1 Al-Hikmah University, Ilorin, Nigeria

This manuscript addresses an important and timely issue: how researcher development frameworks might better prepare doctoral and early-career researchers for contemporary societal, environmental, and policy challenges.

 The authors propose a new competency model (CARE–KNOW–DO), operationalised through the upSKILL.map self-assessment instrument, and report a Phase-1 validation study conducted at a UK university. The paper is ambitious, policy-relevant, and conceptually engaged with current debates on sustainability, responsible research and innovation, and researcher employability. It also reflects a commendable commitment to open science and practical application. However, the manuscript currently combines a worthwhile conceptual agenda with methodological overreach, excessive length, blurred distinctions between theory and evidence, and stronger empirical claims than the data can sustain. The study should be understood as a promising exploratory pilot, not as a validated framework ready for broad implementation. The manuscript would benefit substantially from clearer structure, tighter writing, more cautious interpretation, and stronger methodological transparency.

 The manuscript repeats key claims multiple times, especially regarding:

 eco-outwards identity

 sustainability/equity as foundational

 aspiration–practice gap

 CARE–KNOW–DO principles

 This obscures the empirical contribution.

 The manuscript sometimes assumes rather than demonstrates that current frameworks are deficient.

 The paper should better acknowledge that:

 Disciplinary excellence remains central to many research careers

 Curiosity-driven/basic science is legitimate

 Not all researchers need policy-facing competencies

 Sample size too small for strong psychometric claims

 n = 40 participants

 28 retained items

 five-factor solution

 This is weak for stable factor extraction.

 The manuscript refers to Exploratory Factor Analysis but uses Principal Component Analysis. These are not identical methods. Participants appear to rate relevance/importance rather than demonstrated competence. Participants were likely predisposed toward sustainability and social impact agendas.

 Reframe the study as a pilot exploratory validation only.

 Clarify the statistical method precisely (PCA vs common factor EFA).

 Avoid claiming validated competency measurement from self-report relevance ratings.

 State limits of self-selection more prominently.

 The manuscript references open materials and data repositories, which is positive. However, it is not fully clear whether all materials required to reproduce results are directly accessible.

 Must Improve

 Provide a direct repository DOI/URL.

 Upload an anonymised quantitative dataset.

 Provide codebook/data dictionary.

 Provide analysis syntax/scripts.

 Clarify access conditions for qualitative excerpts/data.

 Rewrite the conclusion to emphasise exploratory status.

 Restrict claims to this sample/context.

 Present implementation suggestions as hypotheses for future study.

 The manuscript must clearly state that findings represent preliminary exploratory evidence, not established validation. Specify whether PCA or common-factor EFA was conducted and justify the choice. Claims beyond this institution/sample should be reduced substantially. Provide data, items, coding framework, scripts, and repository links. Theoretical arguments about sustainability-oriented research should be distinguished from what the study actually demonstrates empirically.

 This manuscript contains a promising and relevant contribution but currently overstates what can be concluded from a small exploratory pilot study. With substantial revision—especially clearer methodological framing, stronger transparency, reduced rhetorical overreach, and more cautious conclusions—it could become a valuable contribution to researcher development literature.

If applicable, is the statistical analysis and its interpretation appropriate?

Partly

Is the study design appropriate and is the work technically sound?

Partly

Is the work clearly and accurately presented and does it engage with the current literature?

Partly

Are the conclusions drawn adequately supported by the results?

Partly

Are sufficient details of methods and analysis provided to allow replication by others?

Partly

Are all the source data and materials underlying the results available?

Partly

Reviewer Expertise:

Educational Leadership, Research Methodology

I confirm that I have read this submission and believe that I have an appropriate level of expertise to state that I do not consider it to be of an acceptable scientific standard, for reasons outlined above.

# Response

**Published:** 2026-04-28

**Authors:** Alexandra Okada

**Affiliations:** Education, The Open University Faculty of Wellbeing Education and Language Studies, Milton Keynes, England, UK

**Response to Reviewer 3 (Moses Adeleke ADEOYE)** *Manuscript: Developing Researchers' Competencies through CARE–KNOW–DO and upSKILL.map (Open Research Europe 2026, 5:333)* We are very grateful to the reviewer for the rigorous and constructive feedback. **Length and repetition.** sections  1.1–1.2 have been tightened and overlapping passages in sections  3–5 consolidated. “Identity” usage has dropped from 58 to 20 occurrences; redundant summary paragraphs have been removed. **Framework deficiency.** section 1.2 now acknowledges that the Vitae RDF, ResearchComp, and OECD Core Framework address purposes distinct from those of the eco-outwards framework. upSKILL.map is positioned as serving researchers engaged with societal, policy, or sustainability challenges — a constituency currently underserved by existing frameworks — not as a universal model. Disciplinary excellence, curiosity-driven inquiry, and fundamental science are explicitly recognised as equally valid research identities. **Sample size and psychometric claims.** section 3.4 and section 4.1.3 state that n = 40 is below conventional thresholds and that the factor structure is descriptive and tentative, generating hypotheses for Phase-2 confirmatory testing (target n > 200, multi-institutional, pre-registered). **EFA/PCA distinction.** EFA is retained as the methodological framing. PCA with Varimax rotation is specified as the extraction method appropriate for descriptive dimension reduction at this pilot stage (Tabachnick & Fidell, 2013; Costello & Osborne, 2005). Principal Axis Factoring (PAF) with Oblimin rotation has been added as a common-factor robustness check, reported in section 4.1.3: four of five components replicate stably; one (Collaborative Inclusive Leadership) shows method-dependence under oblique rotation. Common-factor EFA is reserved for Phase-2. **Relevance versus competence.** We accept this distinction. Clarifying sentences in section 2.3 and section 3.4 state that Part-2 captures perceived relevance, not demonstrated competence. Phase-2 will pilot behaviourally-anchored items. **Self-selection.** Within narrative inquiry, a self-selected sample of practitioners already engaged with the phenomenon is often more epistemically valuable than a large random sample (Clandinin & Connelly, 2000). section 5.2 acknowledges the limits on generalisation while identifying three converging features that lend analytical weight to the findings. **Exploratory framing.** The Abstract Results sentence now reads *“converge on a five-factor descriptive clustering… as a baseline,”* and its closing sentence specifies *“further multi-institutional confirmation.”* section 6 opens with *“The claims below are theoretical syntheses informed by Phase-1 evidence; empirical generalisations await Phase-2 confirmation,”* describes the five factors as *“preliminary,”* and closes with *“offers a testable framework that, with multi-institutional replication, could inform research culture.”* **Restricted claims and propositional recommendations.** Sector-wide statements across sections  5–6 are qualified to the Phase-1 cohort. R1–R4 in section 5.5 now open with *“Phase-1 findings suggest…,” “Phase-1 evidence suggests…,” “Phase-1 findings point to…,”* and *“Phase-1 competency profiles suggest…”* — testable propositions rather than declarative claims. **Open science.** All repository DOIs have been re-verified in the Data Availability section. SPSS syntax will be deposited prior to publication. **Theory–evidence separation.** Theoretical arguments are flagged with *“we argue”* or *“we propose”*; empirical demonstrations are bounded to the Phase-1 cohort; hypothetical claims for Phase-2 testing are explicitly marked in section 5.4 and R1–R4.

# Response

**Published:** 2026-06-04

**Authors:** Moses Adeleke Adeoye

**Affiliations:** Not Applicable, Nigeria

Accept the response from the author

# Reviewer report: Reviewer response for version 1

**DOI:** 10.21956/openreseurope.23156.r63341

**Published:** 2025-12-10

**Authors:** Mona Holmqvist

**Affiliations:** 1 Lund University, Malmö, Skåne County, Sweden

This article explores how researchers can contribute to sustainable, socially relevant solutions. The theory–practice gap is addressed, and the authors argue that future researchers are not adequately trained to address contemporary challenges. To improve this, an instrument (UpSkill.Map) has been developed and tested. In total, 28 skills based on the framework CARE-KNOW-DO have been investigated. The DO dimension is addressing applying skills through practice and societal engagement. The results show that there are challenges across several areas, including interdisciplinary engagement, impact translation, addressing urgent developmental needs, and training PhD students in these areas. The article is well written and covers an important topic, addressing what research is about and how it is enacted.

 Furthermore, the instrument is evaluated both qualitatively and quantitatively. The participants belong to the same institution, and many of them are in the fields of wellbeing, education, and language (35.7%), which may affect the results. However, the authors address this, but not the intense interest this group has in the area studied, which might yield different results for participants in other fields. The quantitative analysis found that the most important skills were not in bridging the research gap, but rather in communicating the researcher’s results. It is not problematized that we have difficulties disseminating research results, which might point to a limited need for the research as such. The questions about how researchers deliver results that support handling contemporary challenges, in relation to who defines the challenges, are not addressed.

 Furthermore, at p. 11, “translate research into policy” points to a view of implementation as something to be negotiated, and thereby gives the impression that it is not negotiated in the context where the results are planned to be used. This might be elaborated on further. At p. 13, Box 1, the challenge of aligning research with society is also mentioned. The authors suggest that there should be more training for researchers and PhD students to communicate research results outside academia; however, the question of how the research is situated and addresses societal challenges is not discussed. Societally engaged research grounded with and for society could have been addressed more thoroughly. This is also lacking in the recommendations at p 19, where all five suggestions address the academic environment but not the societal challenges and how to make an impact in practice. The authors also discuss translating research (p. 19) rather than practice- or society-embedded research.

 Nevertheless, the instrument UpSkill. Map could be more focused on how to address and negotiate the research focus with society and the challenges encountered in practice when planning research instead of afterwards. This was not tested in the study; however, it could be discussed as a further development, as the qualitative analysis points to such difficulties. Maybe the authors might benefit of reading the special issue “Real impact”: Challenges and opportunities in bridging the gap between research and practice – Making a difference in industry, policy, and society (Ref 1)

If applicable, is the statistical analysis and its interpretation appropriate?

I cannot comment. A qualified statistician is required.

Is the study design appropriate and is the work technically sound?

Yes

Is the work clearly and accurately presented and does it engage with the current literature?

Yes

Are the conclusions drawn adequately supported by the results?

Yes

Are sufficient details of methods and analysis provided to allow replication by others?

Yes

Are all the source data and materials underlying the results available?

Yes

Reviewer Expertise:

Higher education, research leadership, doctoral supervision and courses, teaching and learning.

I confirm that I have read this submission and believe that I have an appropriate level of expertise to confirm that it is of an acceptable scientific standard, however I have significant reservations, as outlined above.

## References

[1] : “Real impact”: Challenges and opportunities in bridging the gap between research and practice – Making a difference in industry, policy, and society. *International Journal of Information Management* .2024;78: 10.1016/j.ijinfomgt.2023.102750 10.1016/j.ijinfomgt.2023.102750

# Reviewer report: Reviewer response for version 1

**DOI:** 10.21956/openreseurope.23156.r63819

**Published:** 2025-11-25

**Authors:** Amelie E Trinidad

**Affiliations:** 1 Davao del Sur State College, Matti, Digos City, Davao del Sur, Philippines

**Review Report**

**Manuscript Title:** *Developing Researchers’ Competencies through CARE–KNOW–DO and upSkill.Map, aligned with EU and UNESCO Priorities*

**Authors:** Okada A., Sheehy K., Dieter Rossade K., & Bandara A.K.

**Overall Recommendation:** **Approved with Reservations** 

**A. Clarity and Engagement with Literature - Partly**

 The manuscript is conceptually well-grounded and draws on a broad range of up-to-date literature on researcher development, responsible research and innovation (RRI), sustainability, and equity. The theoretical integration is commendable. However, the introduction is lengthy and contains some repetition. A more concise articulation of the study’s unique contribution relative to existing frameworks (e.g., RMComp, Open Science Skills Framework) would strengthen the argument. More explicit clarification of the instrument’s novel added value is encouraged.

**Suggested Revision:**

 Condense the introduction and clearly articulate how *upSkill.Map* advances or complements existing competency frameworks.

**B. Study Design and Technical Soundness - Partly**

 The use of design-based research (DBR) is appropriate for iterative instrument development, and the combination of quantitative and qualitative strategies enhances methodological depth. However, several issues arise in the quantitative phase:

- The EFA is based on **n = 40**, a very small sample for stable factor extraction.
- No supplemental procedures (e.g., parallel analysis, MAP test, bootstrapping) are reported to support factor retention.
- The DBR cycles would benefit from more detailed operational description.

**Suggested Revisions:**

- Provide methodological justification for conducting EFA with a small sample, supported by relevant literature.
- Report at least a parallel analysis or justify its omission.
- Elaborate on the DBR process by specifying the decisions and iterative refinements across cycles.

**C. Replicability of Methods - Partly**

 While the general analytical approach is described, several essential details are missing:

- The full set of original instrument items is not provided.
- Items removed during EFA are not explicitly identified.
- The qualitative analysis lacks transparency regarding coding process, number of coders, intercoder reliability, and theme development.

**Suggested Revisions:**

- Provide the complete original item list in an appendix or supplemental file.
- Identify the removed items and indicate the statistical criteria for removal (e.g., low factor loadings, low communalities).
- Expand the qualitative methods section by clarifying coder roles, coding procedures, agreement metrics, and the analytical audit trail.

**D. Availability of Source Data - Yes**

 The authors provide complete datasets and associated materials through ORDO. This is commendable and fully aligned with ORE’s open-science requirements. A brief clarification regarding whether qualitative excerpts are available or subject to ethical restrictions would enhance transparency.

**E. Statistical Analysis and Interpretation - Partly**

 The statistical approach is appropriate, but the reporting lacks necessary detail for methodological robustness:

- Full EFA results (factor loadings, communalities, eigenvalues, variance explained) are not included.
- The limitations of the small sample size are not sufficiently acknowledged.

**Suggested Revisions:**

- Include full EFA tables and diagnostic outputs.
- Explicitly discuss the potential instability of the factor solution due to sample size.
- Conduct (or explain the absence of) additional checks such as parallel analysis.

**F. Support of Conclusions by Results - Partly**

 The conclusions generally align with the findings and the conceptual grounding of the study. However, some claims imply broader institutional or sector-level implications that may not be fully supported by this preliminary dataset

 .

**Suggested Revision:**

 Reframe the conclusions to avoid overstating generalizability and clearly differentiate empirical findings from aspirational or theoretical recommendations.

**Additional Suggestions**

*(Optional but beneficial to the manuscript)*

- Add a schematic model illustrating relationships among the CARE–KNOW–DO components, the 8Cs, and the derived factors.
- Provide a concise implementation guide to help institutions adopt *upSkill.Map*.
- Streamline the manuscript through improved sectioning and clear subheadings.

**Overall Recommendation: Approve with Minor Revisions**

 This study represents a valuable and timely contribution to researcher development and aligns well with European and UNESCO-linked priorities. Addressing the methodological and reporting issues noted above will substantially enhance the manuscript’s clarity, rigour, and broader impact.

If applicable, is the statistical analysis and its interpretation appropriate?

Partly

Is the study design appropriate and is the work technically sound?

Partly

Is the work clearly and accurately presented and does it engage with the current literature?

Partly

Are the conclusions drawn adequately supported by the results?

Partly

Are sufficient details of methods and analysis provided to allow replication by others?

Yes

Are all the source data and materials underlying the results available?

Partly

Reviewer Expertise:

My areas of expertise include educational research, researcher development, competency frameworks, mixed-methods research, and instrument development/validation. I am qualified to assess the conceptual framework, study design, statistical analysis (including factor analysis), qualitative methods, and overall methodological rigor. I am not an expert in domain-specific content outside the scope of researcher competencies (e.g., specific EU or UNESCO policy implementation), so my review focuses primarily on methodological and analytical aspects.

I confirm that I have read this submission and believe that I have an appropriate level of expertise to confirm that it is of an acceptable scientific standard, however I have significant reservations, as outlined above.

# Response

**Published:** 2026-02-10

**Authors:** Alexandra Okada

**Affiliations:** Education, The Open University Faculty of Wellbeing Education and Language Studies, Milton Keynes, England, UK

REQUEST CHANGES:

 Condense the introduction and clearly articulate how *upskill. Map* advances or complements existing competency frameworks.

 A. Clarity and Engagement with Literature

 ✓ Addressed: New introduction with clear articulation of how upSkill.Map advances existing frameworks

 ✓ Added comparative Table 1 showing distinctions between ResearchComp, Vitae RDF, OECD Framework, and upSkill.Map o

 ✓ Enhanced theoretical contribution explanation (Section 1.3) Provide methodological justification for conducting EFA with a small sample, supported by relevant literature. Report at least a parallel analysis or justify its omission. Elaborate on the DBR process by specifying the decisions and iterative refinements across cycles.

 B. Study Design and Technical Soundness

 ✓ Addressed: Expanded methodological justification for small sample EFA (n=40)

 ✓ Added detailed justification with relevant literature references

 ✓ Included parallel analysis results with 1,000 Monte Carlo simulations

 ✓ Added explicit acknowledgment of sample size limitations

 ✓ DBR process expanded with explanations Provide the complete original item list in an appendix or supplemental file. Identify the removed items and indicate the statistical criteria for removal (e.g., low factor loadings, low communalities). Expand the qualitative methods section by clarifying coder roles, coding procedures, agreement metrics, and the analytical audit trail.  

 C. Replicability of Methods  

 ✓ Addressed: Provided complete original 32-item instrument list in appendix

 ✓ Identified four removed items with specific statistical criteria (communalities <0.30, cross-loadings >0.47)

 ✓ Expanded qualitative methods section with coder roles, coding procedures, audit trail documentation The authors provide complete datasets and associated materials through ORDO. This is commendable and fully aligned with ORE’s open-science requirements. A brief clarification regarding whether qualitative excerpts are available or subject to ethical restrictions would enhance transparency.

 D. Source Data Availability

 ✓ Confirmed: Complete datasets available via ORDO

 ✓ Clarified that qualitative excerpts are available in the database Full EFA results (factor loadings, communalities, eigenvalues, variance explained) are not included.    

 E. Statistical Analysis and Interpretation

 ✓ Addressed: Included full EFA tables and diagnostic outputs in appendix

 ✓ Explicitly discussed potential instability due to small sample size

 ✓ Added parallel analysis justification

 ✓ Provided detailed variance explanation (75.69% total; individual factors: 19.83%, 16.23%, 14.47%, 13.47%, 11.69%)  

 The conclusions generally align with the findings and the conceptual grounding of the study. However, some claims imply broader institutional or sector-level implications that may not be fully supported by this preliminary dataset

   F. Support of Conclusions

- ✓ Addressed: Reframed conclusions to avoid overstating generalizability
- ✓ Differentiated empirical findings from aspirational recommendations
- ✓ Clarified preliminary validation stage of research

Additional Enhancements Made (Beyond Reviewer Questions)

- ✓ Added schematic model illustration relationships among CARE–KNOW–DO, 8Cs, and derived factors
- ✓ Provided implementation guide for institutional adoption
- ✓ Improved sectioning and clearer subheadings throughout
- ✓ Enhanced DBR process description with specific details on iterative refinements across cycles.
